# Cardiovascular risk assessment enhanced by automated machine learning in a multi-phase study

**DOI:** 10.1038/s41598-025-24189-z

**Published:** 2025-10-20

**Authors:** Igor Bibi, Daniel Schaffert, Philipp Blanke, Lorenz Illian, Federico Lenzing, Niklas Martin, Jan Leipe, Winfried März, Ksenija Stach, Victor Olsavszky

**Affiliations:** 1 Department of Dermatology, Venereology and Allergy, Medical Faculty Mannheim, Center of Excellence in Dermatology, University Medical Center, Heidelberg University, Theodor-Kutzer-Ufer 1-3, 68167 Mannheim, Germany; 2 SYNLAB Academy, SYNLAB Holding Deutschland GmbH, P5, 7, Mannheim, Germany; 3 Fifth Department of Medicine (Nephrology/Endocrinology/Rheumatology), Medical Faculty Mannheim, University Medical Center, Heidelberg University, Theodor-Kutzer-Ufer 1-3, 68167 Mannheim, Germany; 4 University Medical Center, Heidelberg University, Theodor-Kutzer-Ufer 1-3, 68167 Mannheim, Germany; 5 Division of Rheumatology, Department of Internal Medicine I, University Medical Centre Schleswig- Holstein, Kiel, Germany; 6 Department of Endocrinology and Diabetology, Klinikum Ernst von Bergmann, HMU-Health and Medical University, Potsdam, Germany

**Keywords:** Automated machine learning, Cardiovascular diseases, Predictive modeling, Risk stratification, Clinical datasets, Machine learning, Predictive medicine, Outcomes research

## Abstract

Cardiovascular diseases (CVDs) are the leading cause of death worldwide, and current predictors such as lipoprotein (a) [Lp(a)] and risk scores have limitations. Automated machine learning (AutoML) offers the potential to improve CVD risk prediction by processing large datasets and developing tailored models without the need for extensive data science expertise. Using clinical datasets from the LURIC (*n* = 3316) and UMC/M (*n* = 423) studies, we built AutoML models to predict Lp(a), specific CVDs and CVD-related mortality in three phases. Phase 1 identified key CVD determinants such as age, Lp(a), troponin T, BMI and cholesterol with good accuracy (AUC 0.6249 to 0.9101). Phase 2 validated models in the UMC/M dataset and showed robust performance (AUC 0.7224 to 0.8417), with SHAP analysis highlighting predictors like statin therapy, age and NTproBNP. Phase 3 focused on cardiovascular mortality prediction, achieving high AUC values (0.74 to 0.85) and showed data drift, highlighting the need for model adjustment.

**Supplementary Information:**

The online version contains supplementary material available at 10.1038/s41598-025-24189-z.

## Introduction

Cardiovascular diseases (CVDs) are the leading cause of death worldwide, accounting for an estimated 31% of global deaths^1^, or more than 18.6 million annually^2^. The number of people affected continues to rise, with 523 million cases recorded in 2020^3^. This growing burden reflects the increasing incidence of CVDs and the challenges associated with their prevention and management. The interplay between behavioral risk factors and social determinants complicates global control efforts^4^, and despite medical advances, CVDs remain a significant obstacle to achieving sustainable health worldwide^2^.

Therefore, preventive medicine plays a central role in improving cardiovascular outcomes. In clinical practice, clinicians use various tools, including risk assessment scores such as PROCAM^5^, PREVENT^6^, QRISK3^7^ and SCORE2^8^, to help manage comorbidities. However, these scores are limited in their ability to accurately predict CVD events and mortality^9^, and only 20–30% of patients benefit from existing therapies, with some events still occurring despite treatment^10^. This suggests the presence of additional risk factors beyond traditional ones such as age, hypertension, cholesterol, diabetes and smoking^11^.

Lipoprotein (a) [Lp(a)] is a low-density lipoprotein (LDL) cholesterol-like particle attached to apolipoprotein (a), and it is the only known monogenic risk factor for coronary heart disease^12^. Elevated levels are linearly associated with a higher risk of atherosclerotic disease^13^ and affect an estimated 1.4 billion people worldwide^14^. They are also largely genetically determined, which makes them resistant to lifestyle modification^15^. Current therapies are limited: statins may increase Lp(a) levels^16^, while proprotein convertase subtilisin/kexin type 9 (PCSK9) inhibitors only modestly lower them by 25–30%^17^. Novel approaches, such as antisense oligonucleotides and small interfering RNA (e.g. pelacarsen and olpasiran), show promise, but are still being investigated^18^. Despite these advances, Lp(a) remains underdiagnosed and difficult to manage, highlighting the need for further research into CVD prevention and biomarkers^19^.

In this complex landscape, there has been growing momentum behind recommendations to refine cardiovascular risk stratification, yet adding Lp(a) to existing scores has only achieved marginal improvement^13,20^. One challenge is the increasing complexity of medical databases^21^, which makes it difficult to extract clinically relevant insights. Machine learning (ML) has emerged as a potential solution^22^, having already been applied to healthcare systems^23^, disease analysis^24,25^ and predictive modelling for the detection^26^, treatment^27^ and research of Lp(a)-related conditions^28–31^. Although ML can process large datasets efficiently, its adoption in clinical practice remains limited due to the need for data science expertise^32^ and the complexity of implementation^33^. Automated machine learning (AutoML), a subdiscipline of ML, aims to address these barriers by reducing costs, improving health management and supporting clinical research^34^. By enabling non-programmers to build predictive models^35^, AutoML has the potential to improve risk assessment and generate locally tailored models that reflect the demographics and risk dynamics of individual healthcare settings^36^.

The versatility of ML, and AutoML in particular, is evident in its preliminary applications in areas such as CVD prevention in diabetic patients^37^ and their potential role in reshaping the clinical trial landscape. This investigation represents a pioneering effort to understand the impact of Lp(a) and other risk factors through the lens of AutoML within two clinical data sets, the Ludwigshafen Risk and Cardiovascular Health (LURIC) data set^38^ and a local lipidology outpatient clinic dataset, comprising 3,739 participants. Through a comprehensive study framework, including a tripartite structure and methodological progression, we demonstrate the ability of AutoML to identify critical determinants of cardiovascular outcomes, develop robust predictive models for mortality, and validate its predictive capabilities on external datasets. The main research questions of this study are as follows: (Phase 1) What are the key determinants of elevated Lp(a) levels and specific CVDs when analyzed independently in two clinical datasets using AutoML? (Phase 2) Can AutoML models trained on one dataset accurately detect specific CVD events when applied to patients in an external clinical dataset? (Phase 3) Can AutoML models reliably predict 10-year cardiovascular mortality outcomes using different feature sets?

## Methods

### Primary datasets

The first study dataset from the LURIC study spanned data collection from 1997 to 2000, with subsequent follow-ups continuing until 2010, focusing on environmental and genetic risk factors, functional genomics and pharmacogenomics related to cardiovascular health^39^. The LURIC study recruited 3,316 patients from the Rhine-Neckar region of Germany, all of whom underwent coronary angiography. Patients with symptoms of angina pectoris or a positive cardiac stress test were included, while those with other cardiac conditions such as heart failure, valvular heart disease or acute non-cardiac conditions were excluded from recruitment. One year after enrolment, physical examinations and laboratory tests, including genetic tests, were performed. In 2010, follow-up data with information on patient mortality, including date and cause of death, were added to the dataset. This primary dataset comprised 3,058 patient parameters, called features, which were then exported as SPSS files, prepped and consolidated into a single spreadsheet for further integration into our AutoML platform.

For the second dataset, we collected patient records from our lipidology outpatient clinic between 2017 and 2020. Patients diagnosed with familial hypercholesterolemia or CVD and those with elevated CVD risk scores (e.g., PROCAM^40^ score greater than 10%) were recruited. At enrolment, patients underwent clinical and laboratory lipid profiling examinations and immunological testing for lipid biomarkers. In addition, cardiac computed tomography angiography (cCTA) was performed to assess the status of coronary plaques in enrolled patients. Eligible patients received pharmacological therapy, including HMG-CoA reductase inhibitors, ezetimibe and PSCK9 inhibitors. After one year, participants were re-evaluated to assess changes in laboratory findings, coronary artery status and plaque diameter following therapeutic intervention. This second dataset was subsequently enriched with additional clinical data from patient visits, such as cardiac echography and carotid artery duplex scan results, as well as additional laboratory data. Moreover, high-risk patients underwent genetic testing to assess ApoB, LDL and PCSK9 receptor status. Data from 423 patients with 267 features were collected for the second study. This dataset is henceforth referred to as the University Medical Centre Mannheim (UMC/M) dataset and, similar to the LURIC study data, was consolidated and prepped into a single Excel spreadsheet for ease of access and integration into our analysis.

### Preparation of primary datasets and creation of secondary datasets

Prior to deploying the data on the AutoML platform, we enriched the existing UMC/M dataset with additional features. In this process, certain numerical variables were transformed into categorical data to facilitate a more comprehensive analysis (Supplementary Table S1). For example, we categorized body mass index values into different categories, including normal weight, underweight, overweight, and obese. Similarly, LDL levels were categorized as normal, near optimal, borderline high and very high. HbA1c laboratory test results were grouped into categories describing normal, pre-diabetes and manifest diabetes. In addition, certain features, such as single-vessel or multivessel coronary artery disease (CAD), were combined into a binary representation, i.e., a “yes” or “no” feature labeled “any CAD”. We also summarized specific UMC/M features such as early CVD, early peripheral artery disease and early chronic venous insufficiency into a new collective feature called “early cardiovascular conditions” or “early CV conditions” for brevity. Features with insufficient patient data, such as laboratory test results from follow-up visits in the UMC/M dataset, were excluded from the analysis to maintain data integrity and improve later modeling accuracy. An exception was made for the analysis of early cardiovascular disease, in which we retained laboratory data from additional follow-up visits. In addition, we transformed the Lp(a) data, originally presented as numerical values, into binary columns using a cut-off of 50 mg/dL for both datasets. This binary Lp(a) transformation was the only common modification made to both the UMC/M and LURIC datasets. For better understanding, all abbreviated feature names of both datasets are listed in Supplementary Table S2.

Given the similarity between the two primary datasets and their common focus on cardiovascular health, we focused on reducing each dataset to their common features (Supplementary Figure S1). We created a reduced version of the LURIC dataset by retaining only the features found in both datasets and named it “LURIC-Common”. Similarly, we created a reduced version of the UMC/M dataset, which we named “UMC/M-Common”. These new datasets maintain a consistent structure, allowing for easier comparative analysis. The reduced datasets contain 36 common features (Table 1 and Supplementary Table S3). Only features with common definitions across cohorts were used, with consensus achieved among the study investigators supervising the dataset creation.

Finally, for “End of Life” (EoL) targeted analyses, four distinct feature lists from the “LURIC” dataset were curated. The first feature list, called “LURIC-Common/EoL-1”, contains the features from “LURIC-Common” and an additional feature called “CV-EoL”, which indicates whether the enrolled patients had died from cardiovascular causes by their follow-up appointment in 2010 (Table 1). A second feature list, “EoL-2”, was aligned with the parameters used in the ESC cardiovascular mortality score (SCORE2)^8^. In addition, two more EoL prediction sets were formulated through consensus-driven discourse among the study investigators and based on the commercially available CoroPredict^®^ test^41^. “EoL-3” consists of 30 total features, while “EoL-4” consists of 12 features. Notably, the “EoL-4” parameters are included in “EoL-3”, supplemented by a number of laboratory features, which may contribute to increased accuracy in predictions involving the latter (Supplementary Table S3).

**Table 1:** Common Features found in the LURIC and UMC/M datasets. The common feature list has been used throughout phase 2 modeling for creating models based on LURIC data and for later external validation using UMC/M data with the same feature list. For EoL-1, the same feature list was used to predict 10-year cardiovascular mortality with ‘CV-EoL’ as an additional feature used as target for the EoL-1 model.

Common Features: LURIC and UMC/M		
sex cadyn strokeyn carosten pvdyn dm1yn dm2yn insuthyn afibyn height weight chol	ck hba1c homocys vitd25 crp etg eapoa1 eapob elpa vldlch ldlch hdlch	supercrp lvangio age bmi smoclass miyn acsyn earlycad pbnpl1 statinyn hypten COPDyn

### Automated machine learning analysis

**Fig. 1:**
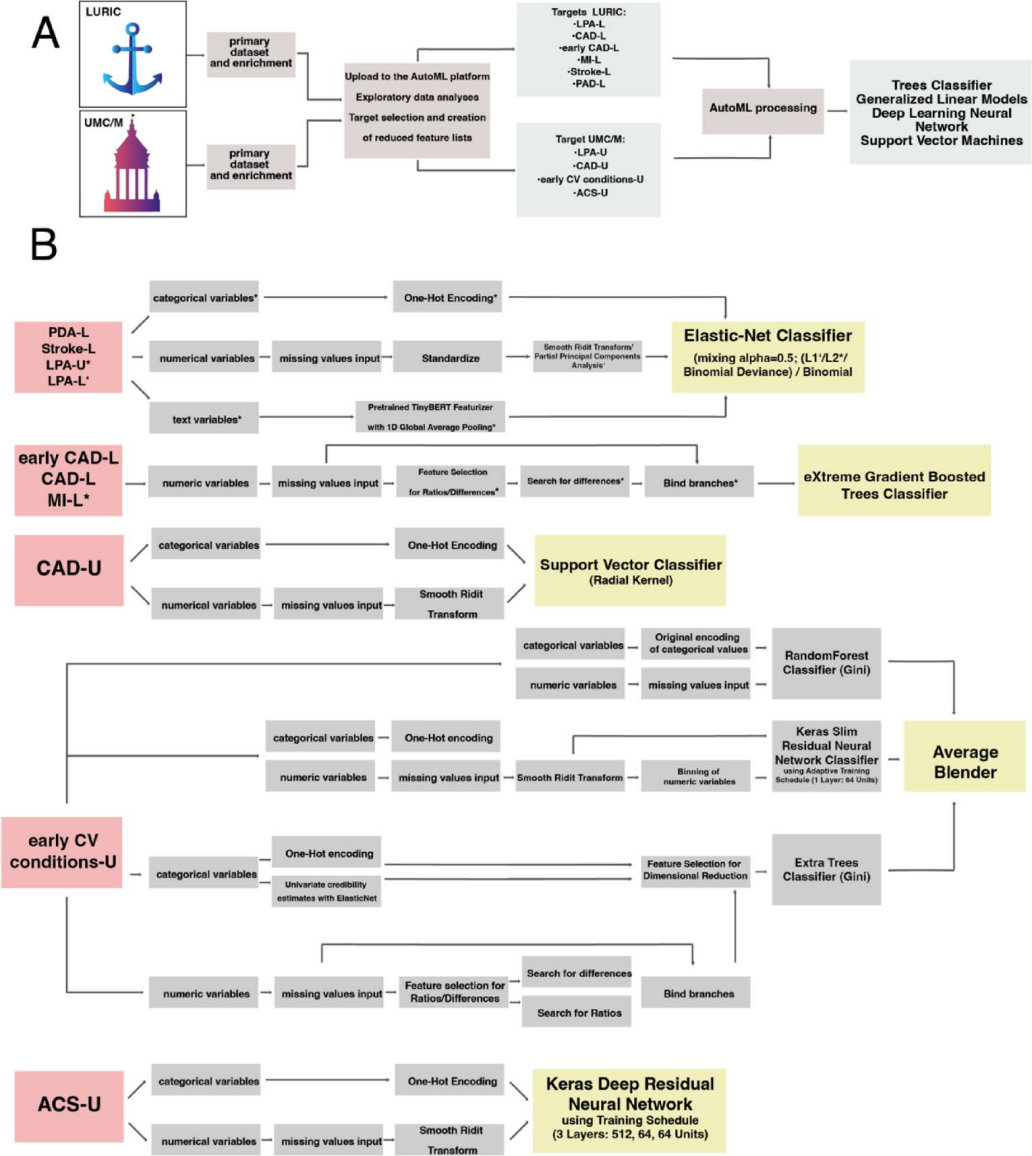
Graphical representation of the study workflow and model blueprints for Phase 1. Flowchart (**A**) of the data processing and modelling workflow for both the LURIC and UMC/M datasets, including the selection of appropriate target features. Individual blueprints (**B**) for the modelling steps for each target are shown graphically. Starting with the targets (pink), through the modelling steps (grey), to the individual models (yellow). Special characters such as apostrophes and asterisks associated with model targets indicate that specific modelling steps, also marked with the same character, were uniquely applied to that particular target model. This notation is used to indicate additional processing steps for specific models. Note that L1 and L2 regularization, also called lasso and ridge regression, represent machine learning techniques that discourage more complexity in models by introducing a penalty term in the cost function. This results in less overfitting in the models.

A comprehensive methodological description of DataRobot’s AutoML analysis has been described previously^42,43^. Based on its ability to systematically generate and compare a wide range of machine learning algorithms under uniform conditions, we decided to employ the aforementioned platform. This ensures objective benchmarking of model performance, reduces manual bias in model development, and enables integration of various modeling approaches, which would otherwise require substantial time and resources to implement individually. Additionally, automated processes for feature handling, hyperparameter optimization, and reproducibility through seed re-runs provide transparency in performance evaluation. In short, after uploading a dataset to the AutoML platform, exploratory data analyses (EDA) were performed to identify the types of features present, such as categorical, numerical, binary, or date features. In order to proceed to the subsequent modeling phase, a selection of target features to be analyzed is required (Fig. 1A). In this study, we selected several target features that can be broadly classified into three main groups, namely Lp(a), specific CVDs, and EoL. Individual target names are appended with ‘-L’ for targets from the LURIC dataset and ‘-U’ for targets from the UMC/M dataset, or have ‘Common’ added to the name if their analysis was performed with the “LURIC-Common” and “UMC/M-Common” datasets (Supplementary Table S4).

After target selection, during a second EDA, the AutoML platform evaluates the impact of the dataset features on the selected target, and automatically generates further reduced feature lists to ensure the exclusion of potential data leakage and features of low importance, thereby improving the modeling process. A complete list of manually extracted and automatically excluded features used for modelling for each target is provided in Supplementary Table S5, Supplementary Table S6 and Supplementary Table S3.

During modelling, the dataset is typically divided into three sets, training, validation and holdout sets, based on stratified or random partitioning (Supplementary Table S7)^42^. Upon completion, all ML models are ranked on the platform’s leaderboard based on their respective accuracy metrics. For this study, we relied primarily on area under the curve (AUC), while log loss values and and maximum Matthews correlation coefficient (Max MCC) were also considered for confirmation. Based on the highest ranked AUC value, we selected the best performing model from the leaderboard for each target for further analysis and prediction. To minimize overfitting, models were trained and evaluated using 5-fold cross-validation with stratified sampling to ensure balanced feature distributions. Model selection relied on mean cross-validation AUC across folds, complemented by holdout performance and, where possible, external validation on the UMC/M dataset. Models failing to meet prespecified AUC thresholds were excluded from further consideration. In Phase 1, we retained models with a cross-validation AUC greater than 0.62. In Phase 2, we only considered models that were trained using the LURIC-Common dataset and had a cross-validation and external validation AUC of at least 0.70 on the UMC/M-Common dataset. Due to the lack of external mortality data in Phase 3, models were ranked by cross-validation AUC alone, with a cutoff of at least 0.70. The validation, cross-validation (CV) and holdout scores of each selected model for each target feature are shown in Supplementary Table S4. To provide additional measures of uncertainty for performance metrics, we manually calculated confidence intervals for model metrics by re-running the model on ‘data seeds’^43^. To do this, we reran all our models for validation, CV and holdout splits with a total of 10 different random seeds (Supplementary Table S4). This allows us to assess the reliability of a model on different data splits. Other indicators of model accuracy were considered by evaluating lift charts and examining model metric results at each stage of validation. Finally, the combinations of data pre-processing steps, transformations and ML computations are visualized as blueprints for each selected model (Fig. 1B, Supplementary Figure S2). Supplementary Table S7 reports on the handling of data quality, including the handling of missing values. Outliers are detected through Ueda’s algorithm^44^, and target leakages are either calculated and flagged for user review or automatically removed if they surpass a specified threshold.

### Data analysis, machine learning predictions, and explainability

After evaluating the performance of the selected models, we explored the influence of features using the AutoML platform’s “Feature Impact” and “Feature Effects” functions in Phase 1. While Feature Impact shows which features most influence model outputs using either permutation, SHAP (SHapley Additive exPlanations) or tree-based importance methods^45^, “Feature Effects” demonstrates how variations in the value of each feature change model predictions^46^. For the graphical representation of the latter function, we chose partial dependency plots^42^, which show the change in target predictions when a specific feature is modified while all other features are held constant.

The model outputs in Phase 2 and 3 were interpreted using SHAP-based^47^ visualization techniques, based on an open-source algorithm that helps to visualize model insights by contributing to “Feature Impact” and prediction explainability with SHAP beeswarm plots, decision plots, heatmaps and waterfall plots. However, this only applies to models with SHAP value support, where a common feature list for both the LURIC and UMC/M dataset was employed. Beeswarm plots summarize the distribution of SHAP values across all instances in a dataset. In these plots, features are ranked by importance on the y-axis, while the x-axis reflects the range and direction of their SHAP values. Each point represents an individual prediction, with its colour indicating the original feature value (red for high, blue for low and grey for missing data). This enables the simultaneous visualization of both feature impact and feature value. SHAP decision plots were used to show how individual features affected the cumulative model prediction for each patient. In these plots, the x-axis represents the model output in log odds and the y-axis ranks features by importance. Individual prediction trajectories are shown as lines which cumulatively add or subtract the SHAP values of the features as one moves from the model’s base value to its final prediction score. To explore how feature influence varies across the patient population, SHAP heatmaps were employed. Each row of a heatmap corresponds to a feature ordered by importance, and each column represents a patient. Color intensity denotes the SHAP value, with red indicating a positive effect on the model prediction and blue indicating a negative effect. Columns are clustered to group patients with similar patterns of explanation, providing insight into population-wide feature behavior. To further illustrate how the models make decisions at the level of an individual patient, SHAP waterfall plots were generated. These plots decompose the predicted output into the contributions of each feature, starting from the expected value of the model output (*E[f(x)]*). Each subsequent bar shows the incremental change introduced by a feature, with red bars increasing the prediction and blue bars decreasing it, until the final predicted value is reached at the top of the plot.

Finally for the third phase, new patient data from the UMC/M dataset was used to make predictions for the selected EoL models. Specifically, the EoL-1 feature list was used to train EoL models on LURIC data. Afterwards, the best model was deployed as active model in the DataRobot AutoML platform. We then uploaded the UMC/M dataset with the common feature list into the Deployment application for more insights on the model and its handling of new data it has not been trained on. Data drift was also considered, as data changes over time when ML model predictions are used^22^. To prevent the loss of predictive power of the selected models, the AutoML platform’s data drift calculations were taken into account to identify feature and prediction drift over time^48^.

### Ethics and TRIPOD statement

This study was reviewed and approved by the Medical Ethics Committee II of the Medical Faculty Mannheim, Heidelberg University, Germany. The two clinical trials, conducted between 1997 and 2000 (LURIC) and between 2017 and 2020 (UMC/M), whose primary data sets were utilized to create the secondary data set for this study. Both trials were carried out in compliance with the principles of the Declaration of Helsinki (approval number: 2024 − 874). Additionally, for the LURIC study, ethical approval was granted by the Landesärztekammer Rheinland-Pfalz, Germany [approval number: #837.255.97(1394)]. This study adheres strictly to the TRIPOD guidelines (see Supplementary Table S8)^49^.

### Code availability

No custom code was written for model development or training, since the analyses were conducted via the DataRobot AutoML platform’s user interface. However, to support reproducibility, we provide the code used to extract and visualize SHAP values as Supplementary Methods.S2

## Results

### Study overview

This study was orchestrated as a three-phase endeavor to address our research objectives related to Lp(a) and CVD AutoML risk determinant analysis, verification of model reliability using separate patient datasets for training and validation, and lastly the investigation of 10-year CVD associated mortality (Supplementary Figure S1). The first phase was dedicated to the use of AutoML to generate ML models to gain new insights into which dataset features influence specific CVD events and Lp(a) levels using the LURIC and UMC/M datasets independently. Here, we leveraged non-overlapping feature lists that included richer clinical variables unique to each dataset, allowing AutoML analysis to capture insights specific to LURIC and UMC/M individually. In the second phase, AutoML modelling was carried out again, this time focusing on prediction and on a refined feature set using the “LURIC-Common” and “UMC/M-Common” datasets separately, with the aim of assessing the robustness and consistency of model performance when using a second dataset for validation. In this phase, only common features between LURIC and UMC/M datasets were used for AutoML analysis and external validation. The final phase focused on predicting 10-year CVD mortality or EoL.

### Phase 1: AutoML dataset analysis to find determinants of Lp(a) and CVD events

#### Model performance across LURIC and UMC/M datasets

**Fig. 2:**
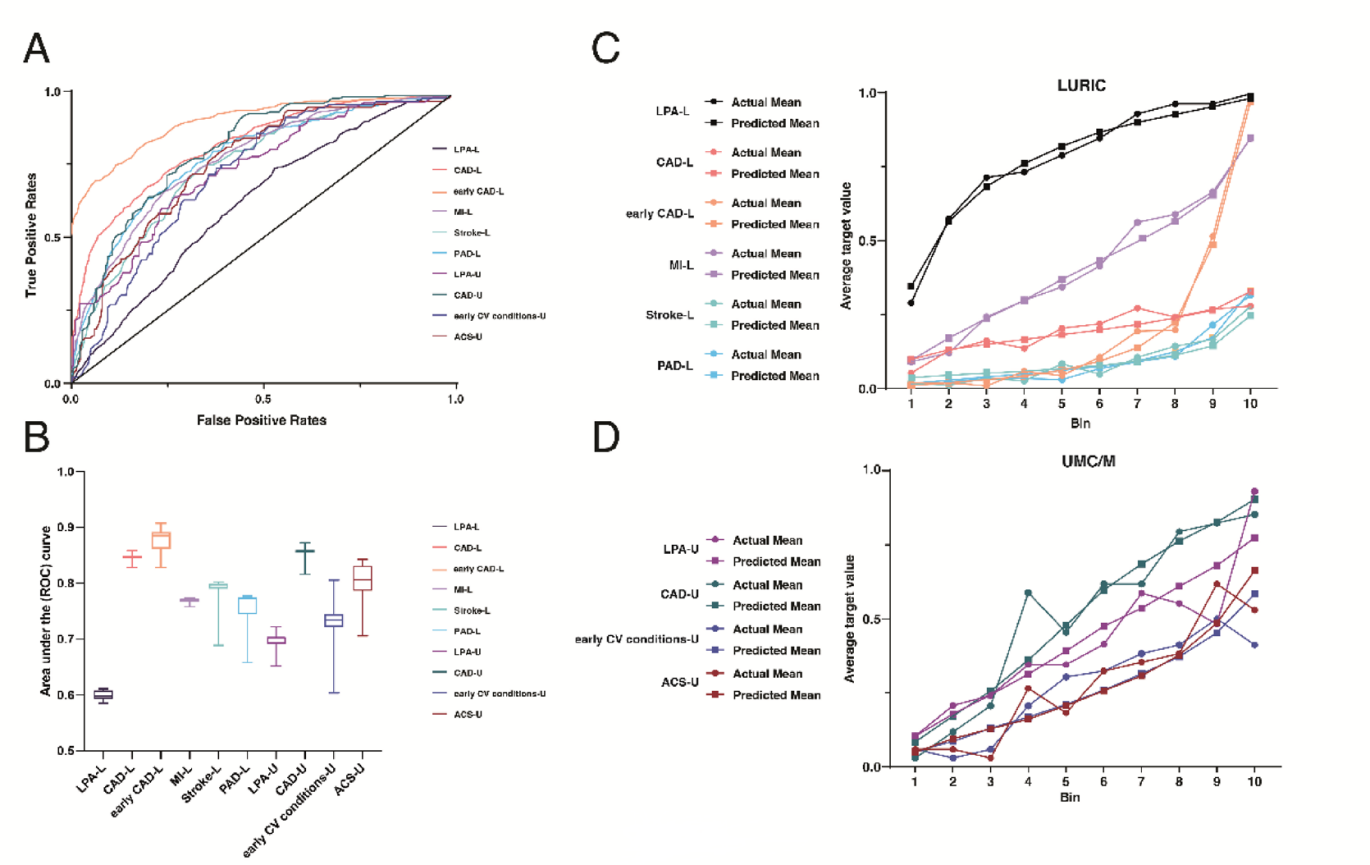
Graphical representation of the model metrics for the cross-validation partition in Phase 1. ROC curves (**A**) for all models showing false positive rates versus true positive rates. The diagonal black line is the line of identity. Mean area under the curve (AUC) values (**B**) in cross-validation for multiple models per target. When modelling for a given target in the ranking reached the cross-validation process, we documented the performance metrics and plotted them here as the mean (horizontal line). The bottom and top of the box represent the 1 st and 3rd quartiles, while the whiskers represent the minimum and maximum performance values. Lift charts of the “LURIC” models (**C**). Lift charts of the “UMC/M” models (**D**). Round and square curve symbols represent actual and predicted average modelling values for a machine learning model, while colors represent individual targets. The average data values for actual and predicted outcomes are segmented into equally sized data bins. Each bin can be traced to its corresponding y-axis value, which represents the percentage of data that falls within the target value.

After careful data preparation, the LURIC and UMC/M datasets were used to train multiple models for targeting Lp(a) and specific CVDs in order to identify determinants in these datasets for the selected target groups (Supplementary Table S4). These targets consisted specifically of the features ‘LPA-L’, ‘CAD-L’, ‘early CAD-L’, ‘MI-L’, ‘Stroke-L’ and ‘PAD-L’ from the LURIC dataset; and the features ‘LPA-U’, ‘CAD-U’, ‘early CV conditions-U’ and ‘ACS-U’ from the UMC/M dataset. For all models, the area under the curve (AUC) values ranged from 0.62 to 0.91 in CV (Fig. 2A). With an average AUC of 0.77, all models demonstrated consistently good accuracy values (Fig. 2B), indicating adequate selectivity in discriminating between events. However, when examining the lift charts^42^ and other performance metrics, it was observed that the selected LURIC models performed better compared to their UMC/M counterparts (Fig. 2C-D, Supplementary Figure S3, Supplementary Table S4). In particular, the best model performance was noted for the target feature ‘early CAD’ in the LURIC dataset with an AUC of 0.9101 (95% CI: [0.9088;0.9114]) (Fig. 2C, Supplementary Figure S3C), whereas the poorest performance accuracy was observed for the prediction of elevated Lp(a), ‘LPA-L’ (Fig. 2C, Supplementary Table S4). In the UMC/M dataset, the model for coronary artery disease, ‘CAD-U’, achieved the best performing AUC metrics of 0.8128 (95% CI: [0.8113; 0.8143]) among the other models using this dataset. Because lift charts illustrate the relative gain in identifying true positives within specific deciles of predicted risk, they tend to highlight local rather than overall model performance. This explains why the lift chart for ‘LPA-L’ appears favorable in the top deciles, even though its overall AUC remains among the lowest across all models.

#### Elevated Lp(a) is conditioned by cardiac function or ezetimibe treatment

**Fig. 3:**
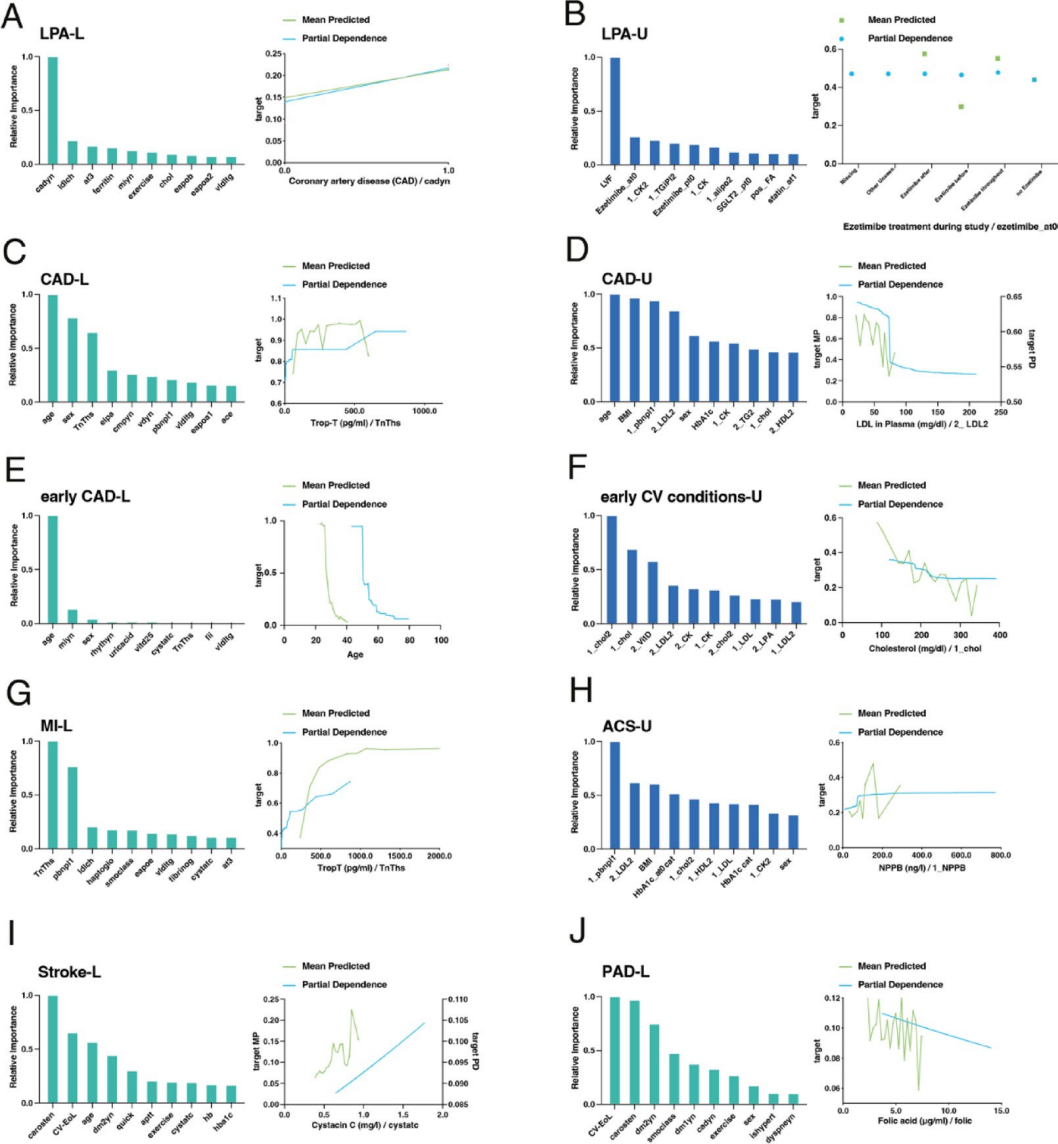
Machine learning model insights for targeted analysis of Lp(a) and specific CVDs. The Feature Impact and Feature Effects of the Phase 1 models for the targets ‘LPA-L’ (**A**), ‘LPA-U’ (**B**), ‘CAD-L’ (**C**), ‘CAD-U’ (**D**), ‘early CAD-L’ (**E**), ‘early CV conditions-U’ (**F**), ‘MI-L’ (**G**), ‘ACS-U’ (**H**), ‘Stroke-L’ (**I**) and ‘PAD-L’ (**J**). Feature Impact is shown to the left of each panel. Impact values have been normalized to 1.00 and are shown for the first ten most impactful features in the list. To the right of each panel is an example of a feature effect. Feature Effects show mean predicted values as a function of different variable inputs for the specific feature and partial dependence (PD) plots. The PD shows the change in target predictions when a particular feature is changed while all other features are held constant.

To gain insight into which features of the datasets most influenced the target in a selected ML model, we evaluated the Feature Impact and Feature Effects functions of each model (Fig. 3, Supplementary Figure S4). For the study of elevated Lp(a), we selected Elastic Net Classifiers models after completing the AutoML modelling on both analyzed datasets. Notably, within the UMC/M training data, 42.1% of patients had elevated Lp(a) levels, whereas this proportion was considerably lower in the LURIC cohort at 19.6%. When examining the Feature Impact, the most influential feature affecting ‘LPA-L’ was the presence of a coronary artery disease, ‘cadyn’, and the most influential feature affecting ‘LPA-U’ was the left ventricular function, ‘LVF’ (Fig. 3A-B). In the case of LURIC, Feature Effects showed that the presence of coronary artery disease was more likely to result in elevated Lp(a) levels when considering both partial dependence (PD) and predicted values (Fig. 3A). In addition, higher antithrombin 3 but lower ferritin levels were more indicative of Lp(a) > 50 mg/dL (Supplementary Figure S4A). Most interestingly, the second most influential feature on ‘LPA-U’ was ‘Ezetimibe_at0’, i.e. whether or not a patient had received ezetimibe treatment, without having previously been prescribed ezetimibe (Fig. 3B). High Lp(a) levels were more strongly linked to lower creatine kinase level (Supplementary Figure S4B).

#### Age is a strong determinant of CVDs, followed by Lp(a), troponin T, BMI and cholesterol

In respect of CAD, 77.9% of patients in the LURIC training data had coronary artery diseases, compared to 51% in the UMC/M training split. Interestingly, both the eXtreme Gradient Boosted Trees Classifier model for ‘CAD-L’ and the Support Vector Classifier model for ‘CAD-U’ favored ‘age’ as the most influential feature (Fig. 3C-D). Notably, the top ten features in both models included ‘sex’ as a target determinant. The main differences between the models were the inclusion of Lp(a) as a top CAD determinant in the LURIC model and BMI as a top CAD determinant in the UMC/M model, whereas these two features were not included in the top ten influential features of their counterparts. In addition, troponin T emerged as the third most influential feature in the LURIC model, but did not appear in the UMC/M model due to its absence from the study measurements. Greater age, Lp(a), BMI or troponin T levels were influential in determining the presence of CAD (Fig. 3C, Supplementary Figure S4C-D), whereas LDL levels > 68 mg/dL were strongly indicative of the absence of CAD in the CAD-U model when PD was taken into account (Fig. 3D). Similarly, ‘age’ had the strongest feature impact for ‘early CAD-L’, with all other features having only minimal influences on this target, the next being myocardial infarction with a normalized permutation importance of 13%, indicating a substantial gap between the impact values of these first two features (Fig. 3E). However, for the UMC/M dataset, the feature impact was more distributed for the target ‘early CV conditions-U’, with cholesterol, vitamin D and low-density lipoprotein (LDL) being the most influential features (Fig. 3F). In terms of data distribution, 21.6% of patients in the LURIC training data had early CAD, whereas 26.8% had early CV conditions in the UMC/M dataset. Feature effects in these cases showed that for ‘early CAD-L’, the likelihood of developing early CAD decreased steeply with increasing age, with PD remaining below the 10% target estimate after the age of 60 (Fig. 3E). Having a myocardial infarction was also indicative of early CAD (Supplementary Figure S4E). On the other hand, increasing cholesterol levels showed a decreasing influence on the target ‘early CV conditions-U’, and LDL at the second study visit has a lower probability of influencing early CV events when it is between 74 and 83 mg/dL in PD (Fig. 3F, Supplementary Figure S4F). Interestingly, the likelihood of such events increased as vitamin D levels at the second study visit rose above 24 µg/dL in PD in the UMC/M model.

#### Determinants of MI, ACS, Stroke, and PAD

For the analysis of myocardial infarction in LURIC (41.7% of training split) and acute coronary syndrome in the UMC/M dataset (28% of training split), NTproBNP emerged as a high impact feature, ranking first in UMC/M and second in LURIC (Fig. 3G-H). The strongest feature impact in ‘MI-L’ was attributed to troponin T, with increasing levels leading to an increased likelihood of myocardial infarction when considering PD, particularly when troponin T levels exceeded 110 mg/dL (Fig. 3G). In both datasets, NTproBNP strongly increased the likelihood of determining myocardial infarction or acute coronary syndrome when it was above 91 ng/dL (Fig. 3H) and approximately 200 ng/ml (Supplementary Figure S4G), considering PD. On the other hand, the PD of LDL dropped sharply between 65 and 74 mg/dL, and when testing for HbA1c, patients with pre-diabetes showed the highest PD for determining ACS-U (Supplementary Figure S4H).

Finally, the selected Elastic-Net Classifier models for Stroke and PAD were meaningfully influenced by carotid stenosis and patient death from cardiovascular causes in 2010 (Fig. 3I-J). Overall, 9.1% of patients had a stroke and 9.3% had peripheral arterial disease in the LURIC training sets. Age emerged as the third most important risk factor for stroke but did not appear in the top ten for PAD. Conversely, diabetes mellitus type 2 was a highly influential feature in both cases. Notably, the presence of carotid stenosis, lower Quick test values and elevated cystatin C levels contributed to an increased risk of stroke, the latter with a feature impact of 19% (Fig. 3I, Supplementary Figure S4I). Decreasing folic acid levels were important in determining the development of peripheral arterial disease (Fig. 3J), whereas CV-EoL in LURIC patients appeared to be a factor indicating possible peripheral arterial disease (Supplementary Figure S4J).

### Phase 2: Consistency evaluation of refined ML models for specific CVD events

#### Model performance on external validation

In phase 2, the aim was to show how well selected ML models performed when confronted with a foreign secondary dataset. To this end, new models were first created using the reduced “LURIC-Common” dataset. The “UMC/M-Common” dataset was then uploaded as secondary data to evaluate the performance of the model in externalizing and performing predictions (Supplementary Figure S1). After AutoML modelling with the “LURIC-Common” dataset, the identification and selection of models for each target was based on the highest AUC score in cross-validation. For the external validation step with “UMC/M-Common” data, a strict partitioning protocol was followed to ensure that this secondary dataset was used exclusively for validation and not included in model training. Notably, models with an AUC performance of less than 0.7 in the validation of the secondary “UMC/M-Common” dataset were not included, nor were models based on the Light Gradient Boosted Trees classifier, as our method of deriving confidence measures by re-running the selected models with different seeds was not supported with this modelling structure. Therefore, the final targets analyzed in phase 2 were the specific CVD events ‘CAD Common’, ‘MI Common’, ‘Stroke Common’, ‘PAD Common’ and ‘ACS Common’.

**Fig. 4:**
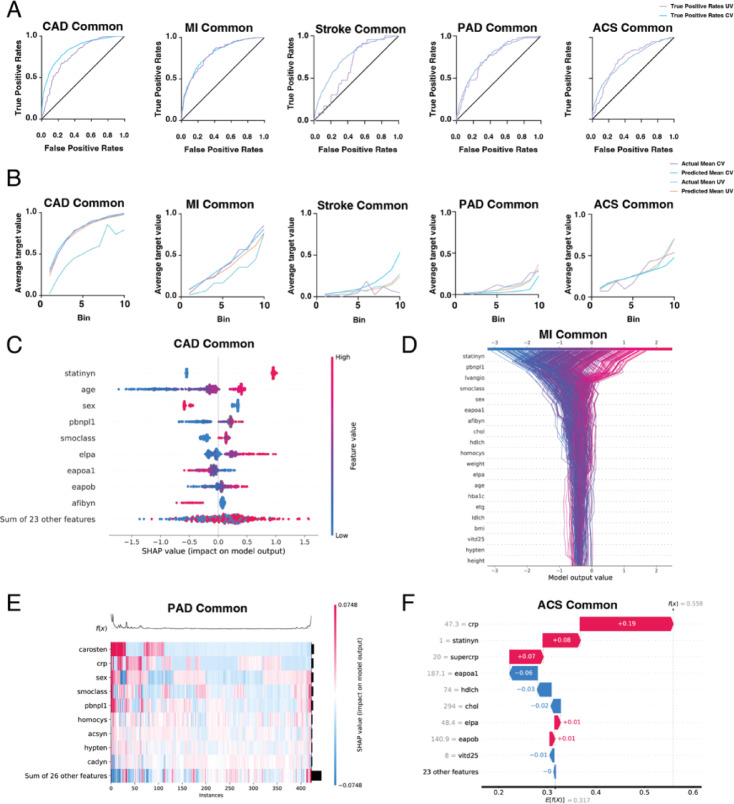
Performance and insights of refined machine learning (ML) models for specific cardiovascular disease (CVD) events, including coronary artery disease (CAD), myocardial infarction (MI), stroke, peripheral artery disease (PAD) and acute coronary syndrome (ACS). The models were trained on the LURIC Common dataset, cross-validated and then externally validated on the UMC/M Common dataset. Receiver operating characteristic (ROC) curves (**A**) and lift charts (**B**) illustrate model accuracy for each CVD event during cross-validation (CV) and external validation (UV). SHAP (SHapley Additive exPlanations) beeswarm plot (**C**) for the ‘CAD Common’ model, showing the impact of different features on the model’s output. Each point represents a SHAP value, with the x-axis indicating the impact of the feature and the y-axis ranking the features by importance. Red points indicate high feature values, blue low values and grey missing data. SHAP decision plot (**D**) for the ‘MI Common’ model, where the x-axis shows the model output value in log odds and the y-axis lists the features. Each line starts at the base value of the model and shows the contribution of each feature’s SHAP value to the final prediction, moving from bottom to top. The SHAP heatmap (**E**) for ‘PAD Common’ highlights the importance of features across the dataset. Each row corresponds to a feature and each column represents a data instance. The color intensity indicates the impact of the SHAP value, with red indicating a positive impact and blue indicating a negative impact. The black line at the top reflects the prediction output of the model for each instance. SHAP waterfall plot (**F**) for the ‘ACS Common’ model, explaining the predictions for a hypothetical patient. The plot shows how each feature contributes to the final log odds prediction, with red indicating positive contributions and blue indicating negative contributions. The expected output of the base model (*E[f(x)]*) is adjusted by the SHAP values to arrive at the final prediction. All SHAP-based visualizations were generated from the UMC/M Common external validation dataset.

The performance of the selected models varied, with AUC values ranging from 0.7224 to 0.8417 in cross-validation and from 0.6465 to 0.8080 in validation against the secondary dataset, hereafter referred to as UMC/M validation or ‘UV’ (Supplementary Table S4). The robustness and accuracy of the models were further corroborated by ROC curves and lift charts (Fig. 4A, B). Examining the ROC curves for the cross-validation of the ‘LURIC-Common’ dataset, all selected models demonstrated good performance, with each curve showing a clear improvement over the diagonal line (Fig. 4A). In addition, for the targets ‘MI Common’ and ‘PAD Common’, the UV curves of the ‘UMC/M-Common’ validation showed less deviation from the CV curves of the ‘LURIC-Common’ dataset, indicating good diagnostic accuracy for these selected models even on external datasets. On the other hand, in the case of ‘Stroke Common’, the UV curves were closer to the diagonal line, suggesting that this model does not discriminate well between stroke cases and non-cases in external datasets. With regard to lift charts, the selected model for the targets ‘ACS Common’ and ‘PAD Common’ had closely matching actual and predicted means in both ‘LURIC-Common’ CV and UV, indicating good model performance (Fig. 4B). Notably, the model selected for ‘CAD Common’ also showed good performance except for the actual mean UV values. Comparing these results with Phase 1, where the average AUC across models was 0.77, it is clear that the overall performance in Phase 2 with an average AUC of 0.74 was slightly lower, but still robust.

#### SHAP-based model interpretability

To better understand how the models arrived at their predictions, we next conducted SHAP analyses. The SHAP beeswarm plots for the selected phase 2 models (Fig. 4C, Supplementary Figure S5A) show how the most influential features on the target in a given dataset affect the model results. Thus, in the case of the selected eXtreme Gradient Boosted Trees Classifier with Early Stopping model for the target ‘CAD Common’, the most influential features were ‘statin therapy’ (‘statinyn’), followed by ‘age’, ‘sex’ and NTproBNP levels (‘pbnpl1’) (Fig. 4C). Notably, while age, sex and NTproBNP levels were also impactful features in the model for the target ‘CAD-L’ (Fig. 3C), having a statin therapy emerged in this study phase as a new and strongest influential feature in predicting whether or not a patient had coronary heart disease. Therefore, considering these first four features, it could be argued that older male patients on statin therapy with high serum brain natriuretic peptide levels are more likely to have coronary artery disease.

SHAP decision plots illustrate how individual features push the predictions up or down, helping to understand the model’s decision process (Fig. 4D, Supplementary Figure S5B). As an example, in the case of ‘MI Common’ (Fig. 4D), we have again chosen an eXtreme Gradient Boosted Trees Classifier with Early Stopping. Here, starting from the least influential feature ‘height’, whose SHAP values do not change the prediction extensively, it is clear that the SHAP values of the features ‘sex’, smoking (‘smoclass’), left ventricular angiography results (‘lvangio’), NTproBNP levels (‘pbnpl1’), and statin therapy (‘statinyn’) contribute notably to the prediction of myocardial infarction when following the prediction lines to the top of the decision plot, with their SHAP values strongly influencing the final model output values. Thus, the likelihood of myocardial infarction is higher in male smokers on statin therapy with abnormal left ventricular angiography results and higher NTproBNP levels.

Next, SHAP heatmaps provide an overview of feature importance across the study population during validation against the secondary dataset (Fig. 4E, Supplementary Figure S5C). For ‘Keras Deep Residual Neural Network Classifier using Training Schedule’ model selected for the target ‘PAD Common’ (Fig. 4E), carotid stenosis (‘carosten’), C-reactive protein (‘CRP’) and ‘sex’ are important influential features, with their positive SHAP values, albeit in few instances in the secondary dataset, leading to positive model prediction outputs. Notably, CRP appears as the second most influential feature in this model for predicting peripheral arterial disease, whereas this feature was not present in the case of the ‘PAD-L’ target analysis (Fig. 3J).

#### SHAP predictions for a hypothetical patient

To illustrate the clinical relevance of these findings, we applied the selected ML models to an exemplary hypothetical patient using SHAP waterfall plots (Supplementary Figure S5D). The hypothetical patient was a 73-year-old male, 1.68 m tall, active smoker with serum cholesterol of 294 mg/dL, triglycerides of 119 mg/dL, NTproBNP of 48 ng/ml and high-sensitivity CRP of 20 mg/dL. He was on statin therapy, had normal results on left ventricular angiography, but suffered from arterial hypertension.

SHAP waterfall plots visualize how each individual feature contributes to the final model prediction for the individual patient (Fig. 4F, Supplementary Figure S5D). In the model target ‘ACS Common’, our hypothetical patient was likely to have had an acute coronary syndrome with the model producing a predicted value, *f(x)*, of 0.559, exceeding the classification threshold. The plot showed that his altered serum levels, especially his elevated CRP, were highly influential in obtaining a positive predictive outcome (Fig. 4F), while few protective variables were present. In contrast, for the ‘MI Common’ model, the patient received a negative prediction, as protective variables like normal NTproBNP and normal angiographic findings outweighed the positive risk contributions from patient variables such as CRP levels or smoking (Supplementary Figure S5D). The patient data yielded positive prediction results for the other CVD targets (CAD, PAD, Stroke), consistent with the patient’s overall high-risk profile, though individual prediction patterns varied throughout models (Supplementary Figure S5D).

#### Dataset-wide prediction outcomes

Finally, we extended the analysis from a single patient to the entire UMC/M cohort. Predictions for each CVD outcome were generated using the final AutoML models trained on the LURIC-Common dataset and validated on the UMC/M-Common dataset (*n* = 423). Using the platform’s default classification thresholds, each model assigned a binary prediction outcome based on the 36 common features to the UMC/M patients. Overall, 362/423 patients were predicted as having had CAD, 130/423 for MI, 25/423 for Stroke, 4/423 for PAD and 14 for ACS.

### Phase 3: cardiovascular mortality risk stratification using AutoML

#### Model performance for mortality prediction

**Fig. 5:**
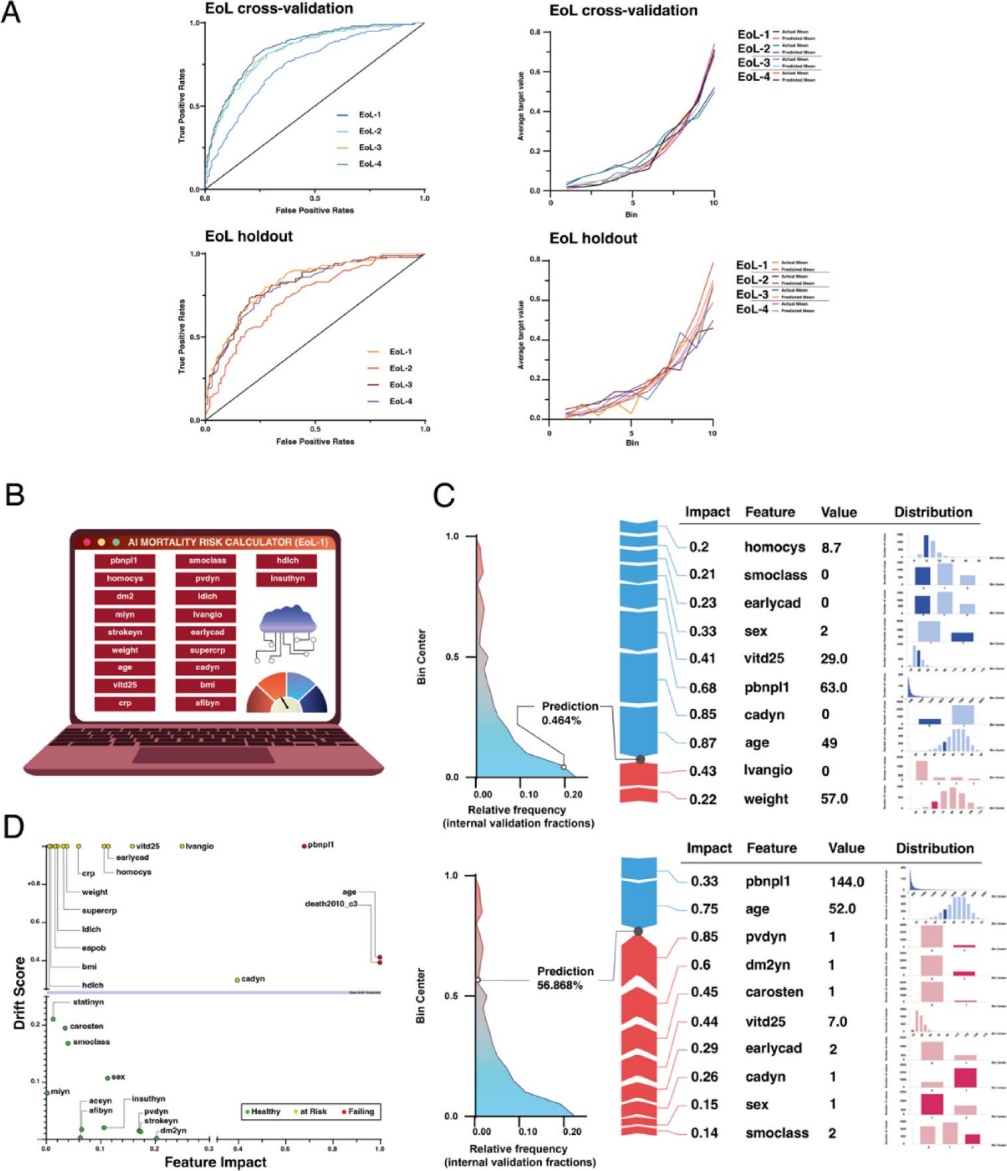
Cardiovascular mortality modeling and its predictive application. **(A)** ROC curves (left) and lift charts (right) for selected EoL models during cross-validation and on holdout sets. **(B)** A graphical illustration of the predictive cardiovascular mortality risk stratification application, showing the top-ranked influential features of the selected EoL1 model. **(C)** Insights for two exemplary patients with their model-predicted risk of death (EoL) are shown in detail, with red arrows indicating an increase in risk due to the specific constellation of feature variables and blue arrows indicating a decrease in risk. Each feature name, its values and its impact on the model output are shown. On the left, the relative frequency plots illustrate the predicted distribution for the hypothetical patients, where patients are categorized into clusters, with color indicating whether the outcome is classified as yes or no (Red = EoL within 10 years). The width of the area for a given prediction probability indicates how many patients in the dataset this prediction applies to. The marker on the graph shows how the patient compares to all others. On the right, the distribution analysis shows the data frequency (y-axis) in the following bins (x-axis) in each histogram. Data drift analysis **(D)** for influential features of the EoL1 model is shown using the UMC/M dataset as an external validation. The influence of the features is plotted against their drift scores, which indicate their deviation from the original training data. Features with a drift score greater than 0.8 were normalized to a maximum value of 1.00. The points are color-coded to represent the health of the features, i.e. whether their data distributions have remained consistent with the training data: green for ‘healthy’, yellow for ‘at risk’ and red for ‘failing’. The data drift threshold is marked to distinguish major drift from negligible changes.

In the final phase of our study, we set out to develop risk categorization models for cardiovascular mortality. These models were extensively trained on four different reduced feature lists from the “LURIC” dataset using the binary endpoint of 10-year cardiovascular mortality, namely the target feature “CV-EoL” (Supplementary Figure S1). After AutoML training and validation, the final models were again selected based on their best AUC scores in the cross-validation of each feature list (Supplementary Table S4), with the selected “EoL-2” model, where predictions were based of ESC cardiovascular mortality score (SCORE2), showing the poorest performance. Nevertheless, all four models had high cross-validation AUC values ranging from 0.74 to 0.85. Notably, “EoL-1” showed the best performance with an AUC of 0.85 (95% CI: [0.8491;0.8491]) with the Regularized Logistic Regression (L2) model during cross-validation and also the best AUC, LogLoss and Max MCC metric scores for the holdout partition, indicating stronger out-of-sample prediction accuracy (Supplementary Table S4). These good predictive accuracies are further supported by the ROC curves in CV and holdout and lift charts plotted for mean actual and mean predicted values for both the cross-validation and holdout data partition sets (Fig. 5A, Supplementary Figure S6).

#### SHAP feature analysis of mortality models

SHAP feature analysis of the training data provides further insight into the ranking of feature importance and how these individual features drive the model (Supplementary Figure S7). In particular, age and NTproBNP ‘pbnpl1’ and vitamin D25 ‘vitd25’ emerged as important features in the majority of EoL models. Looking at the SHAP beeswarm and decision plots, higher age and higher NTproBNP levels appeared to drive the models to produce positive outputs in predicting EoL, while vitamin D25 levels did not show a concentration-dependent effect on model output in the EoL-3 and EoL-4 models, but only in the EoL-1 model did lower vitamin D25 levels appear to be predictive of mortality (Supplementary Figure S7A, B). A scattered effect of individual variables of some features, such as CRP, sex or cystatin C ‘cystatc’, to name a few, is evident when looking at the SHAP heatmaps (Supplementary Figure S7C), suggesting a complex interaction of features influencing EoL predictions. Notably, Lp(a) ‘elpa’ is the fifth most influential feature in EoL-3, but drops to tenth in EoL-4 (Supplementary Figure S7B). In these two models, the distribution of Lp(a) parameter values for SHAP outcomes reflects a scattering in mortality prediction across the patients in the holdout partition (Supplementary Figure S7C).

#### Application to hypothetical patients

Given the superiority of the EoL-1 model, we chose to apply it to two new exemplary hypothetical patients as an AI mortality risk stratification application (Fig. 5B). The first hypothetical patient is a 49-year-old woman with normal NTproBNP levels of 63 ng/ml and vitamin D25 levels of 29 µg/l (Fig. 5C). She is a non-smoker and has no early CAD diagnosis. All these characteristics reduce her risk of cardiovascular mortality. On the other hand, a 52-year-old patient has a higher risk of mortality due to being an active male smoker, having a diagnosis of PAD, type 2 diabetes mellitus, carotid stenosis and low vitamin D25 levels of 7 µg/l, all of which strongly contribute to a positive model output for CV-EoL.

#### External validation and assessment of data drift

To further assess the performance of the EoL-1 model, particularly in the context of data drift, we used the UMC/M dataset as an external validation source. Data drift refers to the deviation of new data from the training data used to develop the model. In particular, there is a divergence in the age distribution between the original training data and the UMC/M dataset, identifying age as a failing feature with substantial drift (Fig. 5D). Similarly, the feature associated with cardiovascular mortality is identified as failing due to the lack of mortality follow-up data in the UMC/M dataset, which prevents an accurate comparison of predicted and actual values. On the other hand, many other features are categorized as ‘healthy’, indicating that their data distributions have remained consistent with the training data. These healthy features suggest that the model’s predictions for these variables are reliable for external validation with new data. Finally, some features are marked as ‘At Risk’, indicating a small amount of data drift. These features have moderate deviations but are not as critical to the overall performance of the model.

Understanding the drift scores and the impact of the features in this way allows real-time adjustments to be made to the predictive tool. As new data features emerge, this insight supports ongoing validation against mortality data, ensuring that the model remains robust and adaptive. As a result, new challenger models can be automatically deployed to address and correct any emerging discrepancies. Overall, 42 out of 423 patients in the UMC/M cohort were predicted as having as positive in the 10-year CV-EoL prediction in the EoL1 model.

## Discussion

By applying AutoML to the LURIC and UMC/M datasets, we were able to identify critical determinants of Lp(a) levels and several CVD outcomes, while developing robust predictive models for cardiovascular mortality. Notably, this study is the first to systematically apply AutoML to assess the relationship between Lp(a) and other risk factors within CVD-specific clinical datasets, highlighting its potential to improve our understanding and risk assessment for developing these conditions. This is consistent with the consensus paper, which highlights the transformative potential of artificial intelligence (AI) in refining cardiovascular risk prediction models and improving personalized medicine^13^.

In the first phase of our investigation, we used AutoML to identify key predictors of elevated Lp(a) and CVD events in both the LURIC and UMC/M datasets, providing new insights into the complex interactions between these determinants and the clinical outcomes of interest. The analysis showed that in the LURIC cohort, the presence of coronary artery disease was the most influential factor associated with elevated Lp(a) levels, whereas in the UMC/M dataset, left ventricular function emerged as the most important predictor. These findings are in line with previous research that has consistently highlighted the role of Lp(a) in the pathogenesis of CAD^50^. Therefore, conversely and according to our findings, the presence of CAD or cardiac dysfunction is already a strong indicator of elevated Lp(a) levels. Furthermore, our analysis identified a negative association between Lp(a) and ferritin levels in the LURIC cohort, echoing previous studies reporting similar but positive associations^51^. However, others have also found a negative association between Lp(a) and ferritin, although not reaching statistical significance^52^. Nevertheless, this finding is particularly intriguing given the established role of ferritin as an acute phase reactant and its potential involvement in atherosclerotic processes, although the causality of this association with Lp(a) remains uncertain and warrants further research. Furthermore, the association observed in our study between increasing antithrombin levels and elevated Lp(a) levels highlights the potential impact of Lp(a) on thromboembolic events. Although Lp(a) is structurally similar to plasminogen and has been implicated in impaired fibrinolysis^13^, its association with thromboembolic events remains controversial^53^. Next, while ezetimibe is widely recognized for its LDL cholesterol-lowering effects, our results suggest that patients treated with ezetimibe were more likely to have elevated Lp(a) levels. Although meta-analysis data show that ezetimibe does not significantly affect Lp(a) levels^54^, the observed association in our study may reflect the complex interplay between underlying cardiovascular disease and the need for intensive lipid-lowering therapy, rather than a direct effect of ezetimibe on Lp(a) levels.

AutoML analysis of specific CVDs further emphasized the importance of Lp(a) as a critical determinant of CAD. In the LURIC dataset, Lp(a) emerged as one of the top predictors of CAD, underscoring its established role in the pathogenesis of atherosclerosis and cardiovascular events. This observation is particularly noteworthy given that traditional risk factors such as age and cholesterol levels have historically dominated CVD risk prediction models^55^, although these predictors also emerged throughout our AutoML analysis for the targets ‘CAD’, ‘early CV conditions’ or ‘stroke’, to name a few. The inclusion of Lp(a) as an influential factor in our AutoML models not only confirms previous studies highlighting its importance^56^, but also suggests that Lp(a) should be systematically included in CVD risk scores to improve the accuracy of cardiovascular risk stratification. This finding is consistent with research showing that incorporating Lp(a) testing into existing risk scores, such as the Reynolds Score, improves cardiovascular risk stratification, particularly for individuals within intermediate-risk cohorts^57^. The potential utility of incorporating Lp(a) into the European Society of Cardiology (ESC) risk score for low-risk countries has also been suggested, further highlighting its relevance for intermediate risk profiles^20^. Notably, patients characterized by high Lp(a) levels and low cardiovascular risk scores have an increased likelihood of vascular events, in contrast to patients with elevated Lp(a) levels and higher CV risk stratification scores^58^. This reinforces the importance of considering Lp(a) in conjunction with other factors when assessing and managing cardiovascular risk. However, the prominence of Lp(a) in the LURIC model for CAD, in contrast to its absence from the top ten features in the UMC/M model, indicates the variability in the influence of Lp(a) in different populations. This variability further suggests the need for localized risk assessment strategies that take into account demographic and clinical differences between patient populations. The inclusion of Lp(a) as an important factor in our AutoML models not only confirms previous studies highlighting its importance, but also suggests that Lp(a) should be systematically included in CVD risk scores to improve the accuracy of cardiovascular risk stratification. This is consistent with findings from INTERASPIRE^59^, which highlighted significant differences in the achievement of guideline-recommended targets for secondary prevention across countries and demographic groups, suggesting that the inclusion of broader risk factors such as Lp(a) could improve risk prediction and management strategies.

Interestingly, the impact of troponin T and NTproBNP levels in predicting myocardial infarction and acute coronary syndrome further validates the importance of these biomarkers in acute cardiovascular events^60^. In addition, our analysis of stroke and peripheral arterial disease (PAD) showed that carotid artery stenosis and type 2 diabetes mellitus were strong predictors, respectively^61,62^. These findings are consistent with the established understanding that these conditions contribute significantly to atherosclerotic disease processes. In particular, the role of diabetes as a major risk factor for both stroke and PAD emphasizes the importance of managing glycemic control in patients at risk for these conditions. Intriguingly, however, the results of our study also draw attention to the role of non-traditional factors, such as hypervitaminosis D influencing the likelihood of early CV events, elevated cystatin C levels contributing to an increased risk of stroke, or decreasing folic acid levels as a determinant of peripheral artery disease. The identification of elevated vitamin D levels as a potential driver of early cardiovascular events highlights that excess vitamin D, most likely caused by excessive supplementation, could lead to several adverse health effects, including cardiovascular complications. Indeed, there are studies showing that it may contribute to cardiovascular diseases by promoting hypercalcemia and calcification in the arteries and other soft tissues, which increases the risk of early cardiovascular events^63,64^. The association between cystatin C and ischemic stroke has been confirmed by a recent meta-analysis, further validating our findings^65^. In addition, prothrombin time has been shown to be an effective predictor of death on hospital discharge in patients with acute ischemic stroke^66^, as we have also found that lower Quick test values are determinants of stroke. Finally, our analysis also highlights the protective role of higher folic acid levels against PAD. Despite the lack of consistent evidence regarding the relationship between folic acid and the incidence of atherosclerosis^67^, one study supports the inhibition of atherosclerotic plaque formation through the regulation of hyperlipidemia and hyperhomocysteinemia-related DNA methylation^68^. Therefore, by preventing the progression of atherosclerosis, folic acid supplementation may play a crucial role in reducing the incidence of these diseases. The identification of such non-traditional factors highlights the need for continuous model adaptation and validation, as emphasized in the consensus paper^13^.

In Phase 2, we sought to determine whether the target CVDs from phase 1 could also be accurately predicted with ML models. We therefore assessed whether the predictions of models trained and tested on the LURIC dataset would be consistent and validated on the UMC/M dataset. To ensure compatibility between the two cohorts, we restricted the analysis to the 36 features shared by both datasets. This reduced the number of input features compared to Phase 1 while keeping the number of patients unchanged. This reduction was unrelated to follow-up data and was implemented to enable external validation using common feature sets. While Phase 2 ensures comparability through the use of common features, Phase 1 was retained as cohort-specific variables, such as troponin T in LURIC or cCTA findings in UMC/M, contribute additional information on cardiovascular pathophysiology that would otherwise be lost if only Phase 2 results were considered.

While the models in Phase 2 for ‘MI Common’ and ‘PAD Common’ showed good predictive accuracy on the external UMC/M dataset, the model for ‘Stroke Common’ had weaker discrimination and overall, the average AUC in Phase 2 (0.74) was slightly lower than in Phase 1 (0.77) but still considered robust. The SHAP feature analysis provided deeper insight into the predictive models by identifying and visualizing the most influential features. Thus, the comparison between Phase 1 and Phase 2 results revealed both consistencies and novel findings in the predictors of CVD outcomes. In the SHAP analysis of the ‘CAD Common’ model in Phase 2, ‘statin therapy’ emerged as the most influential feature, a notable shift from Phase 1 where ‘age’ and ‘Lp(a)’ were among the top predictors of CAD in the LURIC dataset. The prominence of ‘statin therapy’ in Phase 2 suggests that patients currently on statin therapy are more likely to have coronary artery disease, with several studies supporting the idea that statin therapy influences the likelihood of CAD, most likely due to Lp(a) elevation^69,70^. In addition, the SHAP decision plot for ‘MI Common’ emphasized the role of traditional risk factors, including ‘sex’, ‘smoking’, ‘left ventricular angiography results’, ‘NTproBNP levels’ and ‘statin therapy’, as important predictors of myocardial infarction, consistent with the Phase 1 results. However, the introduction of ‘statin therapy’ and the consistent impact of ‘smoking’ in both phases highlight the need to include both treatment history and lifestyle factors in risk prediction models. Next, ‘CRP’ emerged as a critical determinant in the Phase 2 analysis, although it was not highlighted in the Phase 1 analysis for the ‘PAD-L’ target. This suggests that inflammation, as reflected by elevated CRP levels, is also playing a more prominent role in PAD prediction^71^. These Phase 2 results illustrate the dynamic nature of CVD risk prediction, where different data sets and model validations can bring new factors to the forefront. These results suggest that predictive models need to be adaptable and validated across multiple datasets to ensure their robustness and clinical utility.

Although many CVD risk models already exist, their clinical utility remains questionable due to a lack of rigorous validation in real-world settings^72^. Therefore, validation of model accuracy through cross-validation and external datasets, coupled with SHAP analysis, strengthens the reliability of these models and their potential application in personalized medicine. We have demonstrated the clinical relevance and utility of these models by examining the predictions for a hypothetical patient, providing practical insights into the implementation of ML models^43^. The application of AI is already revealing its potential to provide personalized risk assessments^73^ and early disease detection^74^, identifying subtle patterns and anomalies that enable proactive strategies for timely intervention. AI’s adaptability extends to the integration of diverse data sources, including images, lab records and even unstructured clinical notes^75^, often yielding predictive accuracy that exceeds that of traditional scoring systems^76^. Despite the promise, a number of challenges lie ahead. Data bias is a key concern, which can distort predictions and exacerbate health inequalities if under-represented populations are not included^77^. Beyond this, transitioning to cardiovascular mortality risk assessment, the landscape of machine learning applications is still in its infancy^78^. Traditional risk assessment tools, such as the Framingham Risk Score, remain clinically relevant despite the problem of discrepancies in prediction that may not be uniformly reflected in different patient cohorts^79^.

In the final phase of our study, we therefore focused on developing risk stratification models for cardiovascular mortality. The performance of these models, particularly the EoL-1 model based on regularized logistic regression (L2), highlights the potential of machine learning to predict long-term cardiovascular outcomes with high accuracy. Unlike SCORE2, which is based on Cox regression and models hazards over time, our EoL models used a binary classification framework predicting the cardiovascular mortality by the end of follow-up. This methological distinction enables our models to directly estimate the probability of cardiovascular mortality by the end of follow-up, capturing complex, non-linear interactions among predictors that survival-based scores may not fully account for and thereby providing complementary insights into individualized risk. The robust out-of-sample predictive accuracy of this model is further supported by the ROC curves and lift charts, which indicate a high level of reliability in predicting cardiovascular mortality. Application of the EoL-1 model to hypothetical patients demonstrated its practical utility in risk stratification. For example, the model correctly identified a 49-year-old female patient with normal NTproBNP and vitamin D25 levels as having a low risk of cardiovascular mortality, whereas a 52-year-old male patient with several high-risk factors, including PAD, type 2 diabetes mellitus and carotid stenosis, was predicted to have a high risk of mortality. Features such as ‘age’, ‘NTproBNP’ and ‘vitamin D25’ were consistently ranked as influential predictors in the SHAP analysis of the Phase 3 models. Interestingly, while vitamin D25 levels emerged as an important feature, its influence varied across the models, suggesting a more complex relationship with mortality risk that may be context dependent. For example, lower vitamin D25 levels were only predictive of higher mortality in the EoL-1 model, while in other models the effect was less pronounced. Nevertheless, studies have shown that lower 25-hydroxycholecalciferol levels are associated with a higher risk of death^80^. Furthermore, the analysis highlighted the role of Lp(a) in mortality prediction. The non-linear associations observed in our models suggest that Lp(a) contributes to cardiovascular risk in ways not captured by traditional linear approaches. While adding biomarkers such as Lp(a) has yielded only incremental improvements in conventional scores^81^, in our ML framework they substantially improved prediction, particularly in the EoL3 and EoL-4 models.

A critical aspect of Phase 3 was the evaluation of model performance in the context of data drift^82^, using the UMC/M dataset as an external validation source. The identification of drift in age distribution and the lack of follow-up mortality data in the UMC/M dataset highlight the challenges of applying predictive models to new patient populations. By demonstrating how drift analysis and adaptive AI deployments via challenger models can be used to monitor and recalibrate predictive tools, our study highlights a path forward for ensuring model robustness in evolving clinical contexts^83^. This reflects the dynamic nature of AI and underscores importance of continuous oversight to safeguard its transformative potential in healthcare. Traditional care models have proven inadequate in many regions, and our work highlights the capacity of AutoML to deliver more personalized, accurate, and responsive healthcare systems. By integrating these advanced predictive tools into clinical practice, we have the potential to drastically improve patient outcomes and address the global burden of cardiovascular disease. In addition, questions arise when features with high impact on target prediction, not traditionally considered significant by researchers, come into play^76^. This highlights the importance of ongoing research to validate these unconventional predictors and ensure AI-driven models provide accurate, actionable insights.

Several limitations of this study should also be considered. First, the analyses were based on retrospective observational data, which may be subject to selection bias and unmeasured confounding factors. Second, despite harmonization efforts, the LURIC and UMC/M cohorts differ in terms of available clinical variables and diagnostic practices, which may influence cross-cohort comparisons. Third, the absence of mortality follow-up data in the UMC/M cohort prevented the external validation of Phase 3 mortality models, which limits the generalizability of the findings. Fourth, we modeled cardiovascular mortality as a binary endpoint, which did not make use of the available time-to event information. While this design ensured methological consistency across all project phases, extending the analyses to survival-based ML models would facilitate comparisons across different modeling frameworks, given that traditional risk estimators such as SCORE2 are based on survival frameworks like Cox regression^84^. The development of future survival-based ML models represents an important direction for future research. Finally, although SHAP plots improve the interpretability of complex ML models, integrating them into routine clinical decision-making requires translating technical explanations into practical tools for clinicians.

## Conclusion

In conclusion, as medical data continues to evolve, so must the tools we use to analyze and interpret it. Our study not only advances the methodology for cardiovascular disease risk stratification but also lays the groundwork for more personalized, precise, and adaptive healthcare systems. Importantly, we demonstrated that the predictive role of Lp(a) can be more accurately contextualized when evaluated alongside a wide range of clinical, biochemical, and demographic features, rather than in isolation, by re-examining its role within an AutoML framework. This underscores the clinical relevance of Lp(a) and the value of AutoML in revealing its nuanced role in various cardiovascular outcomes. As we move forward, the integration of these cutting-edge predictive models into clinical practice holds great promise for improving patient outcomes and reducing the global burden of cardiovascular disease.

## Supplementary Information

Below is the link to the electronic supplementary material.

Supplementary Material 1

Supplementary Material 2

Supplementary Material 3

Supplementary Material 4

Supplementary Material 5

Supplementary Material 6

Supplementary Material 7

Supplementary Material 8

Supplementary Material 9

Supplementary Material 10

Supplementary Material 11

Supplementary Material 12

## Data Availability

The patients’ clinical data is available from the authors upon reasonable request. Please contact the authors via email at Ksenija.Stach-Jablonski@klinikumevb.de to obtain the dataset.

## Abbreviations

ACS: Acute coronary syndrome
ACS-L/U: Patient with acute coronary syndrome in the LURIC / UMC/M study cohort
ApoB: Apolipoprotein B
AVG: Average blender
CAD-L/U: coronary artery disease in the LURIC / UMC/M study cohort
CI: Confidence Interval
CVD: Cardiovascular disease
CV: Cardiovascular
early CAD-L: early coronary artery disease in the LURIC study cohort
early CV conditions-U: early cardiovascular conditions (early cardiovascular disease, early chronic venous insufficiency, and peripheral artery disease) in the UMC/M study cohort
EGBTC: eXtreme gradient boosted trees classifier
ENET: Elastic-Net Classifier
HbA1c: Glycated hemoglobin
HMG-CoA reductase: 3-hydroxy-3-methyl-glutaryl-coenzyme A reductase
KERA: Kera’s deep residual neural network classifier
LGBTC: Light gradient boosted trees classifier
Lp(a): Lipoprotein (a)
MCC: Mathews correlation coefficient
LPA-L/U: Lipoprotein (a) over 50 mg/dL in the LURIC / UMC/M study cohort
PAD: Peripheral artery disease
PAD-L: Peripheral artery disease in the LURIC study cohort
PROCAM: Prospective cardiovascular münster study
Stroke-L: Stroke in the LURIC study cohort
SVC (RK): Support vector classifier (Radial Kernel)

## References

[1] Kim, H. C. Epidemiology of cardiovascular disease and its risk factors in Korea. *Glob Health Med.* **3**, 134–141. 10.35772/ghm.2021.01008 (2021). 34250288 10.35772/ghm.2021.01008 PMC8239378

[2] Roth, G. A. et al. Global burden of cardiovascular diseases and risk Factors, 1990–2019: update from the GBD 2019 study. *J. Am. Coll. Cardiol.* **76**, 2982–3021. 10.1016/j.jacc.2020.11.010 (2020). 33309175 10.1016/j.jacc.2020.11.010 PMC7755038

[3] Coronado, F., Melvin, S. C., Bell, R. A. & Zhao, G. Global responses to Prevent, Manage, and control cardiovascular diseases. *Prev. Chronic Dis.* **19**, E84. 10.5888/pcd19.220347 (2022). 36480801 10.5888/pcd19.220347 PMC9746707

[4] (WHO). W. H. O. *Cardiovascular diseases (CVDs)*, (2021). https://www.who.int/news-room/fact-sheets/detail/cardiovascular-diseases-(cvds)

[5] Assmann, G. & Schulte, H. The prospective cardiovascular Münster (PROCAM) study: prevalence of hyperlipidemia in persons with hypertension and/or diabetes mellitus and the relationship to coronary heart disease. *Am. Heart J.* **116**, 1713–1724. 10.1016/0002-8703(88)90220-7 (1988). 3202078 10.1016/0002-8703(88)90220-7

[6] Khan, S. S. et al. Development and validation of the American heart association’s PREVENT equations. *Circulation* **149**, 430–449. 10.1161/circulationaha.123.067626 (2024). 37947085 10.1161/CIRCULATIONAHA.123.067626 PMC10910659

[7] Hippisley-Cox, J., Coupland, C. & Brindle, P. Development and validation of QRISK3 risk prediction algorithms to estimate future risk of cardiovascular disease: prospective cohort study. *Bmj* 357, j (2099). (2017) 10.1136/bmj.j2099 10.1136/bmj.j2099 PMC5441081 28536104

[8] collaboration, S. w. g. a. E. C. r. SCORE2 risk prediction algorithms: new models to estimate 10-year risk of cardiovascular disease in Europe. *Eur. Heart J.* **42**, 2439–2454. 10.1093/eurheartj/ehab309 (2021). 34120177 10.1093/eurheartj/ehab309 PMC8248998

[9] Studziński, K., Tomasik, T., Krzysztoń, J., Jóźwiak, J. & Windak, A. Effect of using cardiovascular risk scoring in routine risk assessment in primary prevention of cardiovascular disease: an overview of systematic reviews. *BMC Cardiovasc. Disord*. **19**, 11. 10.1186/s12872-018-0990-2 (2019). 30626326 10.1186/s12872-018-0990-2 PMC6327540

[10] Tsimikas, S. A test in context: Lipoprotein(a): Diagnosis, Prognosis, Controversies, and emerging therapies. *J. Am. Coll. Cardiol.* **69**, 692–711. 10.1016/j.jacc.2016.11.042 (2017). 28183512 10.1016/j.jacc.2016.11.042

[11] Kannel, W. B., Dawber, T. R., Kagan, A., Revotskie, N. & Stokes, J. 3rd Factors of risk in the development of coronary heart disease–six year follow-up experience. The Framingham study. *Ann. Intern. Med.* **55**, 33–50. 10.7326/0003-4819-55-1-33 (1961). 13751193 10.7326/0003-4819-55-1-33

[12] Tsimikas, S. et al. Antisense therapy targeting apolipoprotein(a): a randomised, double-blind, placebo-controlled phase 1 study. *Lancet* **386**, 1472–1483. 10.1016/s0140-6736(15)61252-1 (2015). 26210642 10.1016/S0140-6736(15)61252-1

[13] Kronenberg, F. et al. Lipoprotein(a) in atherosclerotic cardiovascular disease and aortic stenosis: a European atherosclerosis society consensus statement. *Eur. Heart J.* **43**, 3925–3946. 10.1093/eurheartj/ehac361 (2022). 36036785 10.1093/eurheartj/ehac361 PMC9639807

[14] Tsimikas, S. & Stroes, E. S. G. The dedicated Lp(a) clinic: A concept whose time has arrived? *Atherosclerosis* 300, 1–9 (2020). 10.1016/j.atherosclerosis.2020.03.003 10.1016/j.atherosclerosis.2020.03.003 32234580

[15] Enkhmaa, B., Petersen, K. S., Kris-Etherton, P. M. & Berglund, L. Diet and Lp(a): Does Dietary Change Modify Residual Cardiovascular Risk Conferred by Lp(a)? *Nutrients* 12 (2020). 10.3390/nu12072024 10.3390/nu12072024 PMC7400957 32646066

[16] Wilson, D. P. et al. Use of Lipoprotein(a) in clinical practice: A biomarker whose time has come. A scientific statement from the National lipid association. *J. Clin. Lipidol.* **13**, 374–392. 10.1016/j.jacl.2019.04.010 (2019). 31147269 10.1016/j.jacl.2019.04.010

[17] Villard, E. F. et al. PCSK9 modulates the secretion but not the cellular uptake of Lipoprotein(a) ex vivo: an effect blunted by Alirocumab. *JACC Basic. Transl Sci.* **1**, 419–427. 10.1016/j.jacbts.2016.06.006 (2016). 29308438 10.1016/j.jacbts.2016.06.006 PMC5753417

[18] Sosnowska, B., Surma, S. & Banach, M. Targeted treatment against lipoprotein (a): the coming breakthrough in lipid Lowering therapy. *Pharmaceuticals (Basel)*. 15. 10.3390/ph15121573 (2022). 10.3390/ph15121573 PMC9781646 36559024

[19] Dhindsa, D. S., Sandesara, P. B., Shapiro, M. D. & Wong, N. D. The evolving Understanding and approach to residual cardiovascular risk management. *Front. Cardiovasc. Med.* **7**, 88. 10.3389/fcvm.2020.00088 (2020). 32478100 10.3389/fcvm.2020.00088 PMC7237700

[20] Delabays, B. et al. Use of lipoprotein(a) for refining cardiovascular risk prediction in a low-risk population: the CoLaus/PsyCoLaus study. *Eur. J. Prev. Cardiol.* **28**, e18–e20. 10.1177/2047487320938271 (2021). 34298560 10.1177/2047487320938271

[21] Obermeyer, Z. & Lee, T. H. Lost in Thought - The limits of the human Mind and the future of medicine. *N Engl. J. Med.* **377**, 1209–1211. 10.1056/NEJMp1705348 (2017). 28953443 10.1056/NEJMp1705348 PMC5754014

[22] Weissler, E. H. et al. The role of machine learning in clinical research: transforming the future of evidence generation. *Trials* **22**, 537. 10.1186/s13063-021-05489-x (2021). 34399832 10.1186/s13063-021-05489-x PMC8365941

[23] Salazar-Reyna, R. et al. A systematic literature review of data science, data analytics and machine learning applied to healthcare engineering systems. *Manag. Decis.* **60**, 300–319. 10.1108/md-01-2020-0035 (2020).

[24] Abbasi, B. & Goldenholz, D. M. Machine learning applications in epilepsy. *Epilepsia* **60**, 2037–2047. 10.1111/epi.16333 (2019). 31478577 10.1111/epi.16333 PMC9897263

[25] Jiang, M. et al. Machine learning in rheumatic diseases. *Clin. Rev. Allergy Immunol.* **60**, 96–110. 10.1007/s12016-020-08805-6 (2021). 32681407 10.1007/s12016-020-08805-6

[26] Liang, H. et al. Evaluation and accurate diagnoses of pediatric diseases using artificial intelligence. *Nat. Med.* **25**, 433–438. 10.1038/s41591-018-0335-9 (2019). 30742121 10.1038/s41591-018-0335-9

[27] Vatansever, S. et al. Artificial intelligence and machine learning-aided drug discovery in central nervous system diseases: State-of-the-arts and future directions. *Med. Res. Rev.* **41**, 1427–1473. 10.1002/med.21764 (2021). 33295676 10.1002/med.21764 PMC8043990

[28] Xue, Y. et al. Risk stratification of ST-segment elevation myocardial infarction (STEMI) patients using machine learning based on lipid profiles. *Lipids Health Dis.* **20**, 48. 10.1186/s12944-021-01475-z (2021). 33957898 10.1186/s12944-021-01475-z PMC8101132

[29] Ibrahim, S., Reeskamp, L. F., Stroes, E. S. G. & Watts, G. F. Advances, gaps and opportunities in the detection of Familial hypercholesterolemia: overview of current and future screening and detection methods. *Curr. Opin. Lipidol.* **31**, 347–355. 10.1097/mol.0000000000000714 (2020). 33027222 10.1097/MOL.0000000000000714

[30] Zhao, J. et al. Using topic modeling via non-negative matrix factorization to identify relationships between genetic variants and disease phenotypes: A case study of Lipoprotein(a) (LPA). *PLoS One*. **14**, e0212112. 10.1371/journal.pone.0212112 (2019). 30759150 10.1371/journal.pone.0212112 PMC6374022

[31] Widen, E., Raben, T. G., Lello, L. & Hsu, S. D. H. Machine learning prediction of biomarkers from SNPs and of disease risk from biomarkers in the UK biobank. *Genes (Basel)*. 12. 10.3390/genes12070991 (2021). 10.3390/genes12070991 PMC8308062 34209487

[32] Alsharqi, M. et al. Artificial intelligence and echocardiography. *Echo Res. Pract.* **5**, R115–R125. 10.1530/ERP-18-0056 (2018). 30400053 10.1530/ERP-18-0056 PMC6280250

[33] Tobias, M., Ulle, N. & Shoemaker, T. Fourteen things you need to know about collaborating with data scientists. *Nature*10.1038/d41586-023-02291-4 (2023). 37443305 10.1038/d41586-023-02291-4

[34] Waring, J., Lindvall, C. & Umeton, R. Automated machine learning: review of the state-of-the-art and opportunities for healthcare. *Artif. Intell. Med.* **104**, 101822. 10.1016/j.artmed.2020.101822 (2020). 32499001 10.1016/j.artmed.2020.101822

[35] Benecke, J. et al. Retrospective analysis and time series forecasting with automated machine learning of ascariasis, enterobiasis and cystic echinococcosis in Romania. *PLoS Negl. Trop. Dis.* **15**, e0009831. 10.1371/journal.pntd.0009831 (2021). 34723982 10.1371/journal.pntd.0009831 PMC8584970

[36] Alaa, A. M., Bolton, T., Di Angelantonio, E., Rudd, J. H. F. & van der Schaar, M. Cardiovascular disease risk prediction using automated machine learning: A prospective study of 423,604 UK biobank participants. *PLoS One*. **14**, e0213653. 10.1371/journal.pone.0213653 (2019). 31091238 10.1371/journal.pone.0213653 PMC6519796

[37] Nabrdalik, K. et al. Machine learning predicts cardiovascular events in patients with diabetes: the Silesia Diabetes-Heart project. *Curr. Probl. Cardiol.* **101694**10.1016/j.cpcardiol.2023.101694 (2023). 10.1016/j.cpcardiol.2023.101694 36921649

[38] Marz, W. et al. Low-density lipoprotein triglycerides associated with low-grade systemic inflammation, adhesion molecules, and angiographic coronary artery disease: the Ludwigshafen risk and cardiovascular health study. *Circulation* **110**, 3068–3074. 10.1161/01.CIR.0000146898.06923.80 (2004). 15505088 10.1161/01.CIR.0000146898.06923.80

[39] Winkelmann, B. R. et al. Rationale and design of the LURIC study–a resource for functional genomics, pharmacogenomics and long-term prognosis of cardiovascular disease. *Pharmacogenomics* **2**, 1–73. 10.1517/14622416.2.1.S1 (2001). 10.1517/14622416.2.1.S1 11258203

[40] Assmann, G., Schulte, H., Cullen, P. & Seedorf, U. Assessing risk of myocardial infarction and stroke: new data from the prospective cardiovascular Munster (PROCAM) study. *Eur. J. Clin. Invest.* **37**, 925–932. 10.1111/j.1365-2362.2007.01888.x (2007). 18036028 10.1111/j.1365-2362.2007.01888.x

[41] GmbH, S. H. D. COROPREDICT^®^*-TEST*, (2019). https://www.synlab.de/fileadmin/pdf/fachinformationen/coropredict-test.pdf

[42] Bibi, I. et al. Automated machine learning analysis of patients with chronic skin disease using a medical smartphone app: retrospective study. *J. Med. Internet Res.* **25**, e50886. 10.2196/50886 (2023). 38015608 10.2196/50886 PMC10716771

[43] Schaffert, D. et al. Using automated machine learning to predict necessary upcoming therapy changes in patients with psoriasis vulgaris and psoriatic arthritis and uncover new influences on disease progression: retrospective study. *JMIR Form. Res.* **8**, e55855. 10.2196/55855 (2024). 38738977 10.2196/55855 PMC11240079

[44] Marmolejo-Ramos, F., Vélez, J. I. & Romão, X. Automatic detection of discordant outliers via the ueda’s method. *J. Stat. Distrib. Appl.* **2**, 1–14 (2015).

[45] DataRobot, I. *Feature Impact*, (2023). https://docs.datarobot.com/en/docs/modeling/analyze-models/understand/feature-impact.html

[46] DataRobot, I. *Feature Effects*, (2023). https://docs.datarobot.com/en/docs/modeling/analyze-models/understand/feature-effects.html

[47] Lundberg, S. *shap.decision_plot*, (2018). https://shap-lrjball.readthedocs.io/en/latest/generated/shap.decision_plot.html

[48] Darnell, D. *All Data Drift is Not Created Equal…*, (2020).

[49] Collins, G. S., Reitsma, J. B., Altman, D. G. & Moons, K. G. Transparent reporting of a multivariable prediction model for individual prognosis or diagnosis (TRIPOD): the TRIPOD statement. *Bmj* **350**, g7594. 10.1136/bmj.g7594 (2015). 25569120 10.1136/bmj.g7594

[50] Jang, A. Y., Han, S. H., Sohn, I. S., Oh, P. C. & Koh, K. K. Lipoprotein(a) and cardiovascular Diseases - Revisited. *Circ. J.* **84**, 867–874. 10.1253/circj.CJ-20-0051 (2020). 32336721 10.1253/circj.CJ-20-0051

[51] Chakraborty, B. et al. Lipoprotein(a), ferritin, and albumin in acute phase reaction predicts severity and mortality of acute ischemic stroke in North Indian patients. *J. Stroke Cerebrovasc. Dis.* **22**, e159–167. 10.1016/j.jstrokecerebrovasdis.2012.10.013 (2013). 23253530 10.1016/j.jstrokecerebrovasdis.2012.10.013

[52] Brandt, E. J. et al. Association of vitamins, minerals, and lead with Lipoprotein(a) in a cross-sectional cohort of US adults. *Int. J. Vitam. Nutr. Res.* 1–12. 10.1024/0300-9831/a000709 (2021). 10.1024/0300-9831/a000709 PMC8964024 34024154

[53] Boffa, M. B. Beyond fibrinolysis: the confounding role of Lp(a) in thrombosis. *Atherosclerosis* **349**, 72–81. 10.1016/j.atherosclerosis.2022.04.009 (2022). 35606079 10.1016/j.atherosclerosis.2022.04.009

[54] Sahebkar, A. et al. Impact of Ezetimibe on plasma lipoprotein(a) concentrations as monotherapy or in combination with statins: a systematic review and meta-analysis of randomized controlled trials. *Sci. Rep.* **8**, 17887. 10.1038/s41598-018-36204-7 (2018). 30552391 10.1038/s41598-018-36204-7 PMC6294784

[55] Kondamudi, N., Mehta, A., Thangada, N. D. & Pandey, A. Physical activity and cardiorespiratory fitness: vital signs for cardiovascular risk assessment. *Curr. Cardiol. Rep.* **23**, 172. 10.1007/s11886-021-01596-y (2021). 34647161 10.1007/s11886-021-01596-y

[56] Reyes-Soffer, G., Yeang, C., Michos, E. D., Boatwright, W. & Ballantyne, C. M. High lipoprotein(a): actionable strategies for risk assessment and mitigation. *Am. J. Prev. Cardiol.* **18**, 100651. 10.1016/j.ajpc.2024.100651 (2024). 38646021 10.1016/j.ajpc.2024.100651 PMC11031736

[57] Willeit, P. et al. Discrimination and net reclassification of cardiovascular risk with lipoprotein(a): prospective 15-year outcomes in the Bruneck study. *J. Am. Coll. Cardiol.* **64**, 851–860. 10.1016/j.jacc.2014.03.061 (2014). 25169167 10.1016/j.jacc.2014.03.061

[58] Perrot, N. et al. Ideal cardiovascular health influences cardiovascular disease risk associated with high lipoprotein(a) levels and genotype: the EPIC-Norfolk prospective population study. *Atherosclerosis* **256**, 47–52. 10.1016/j.atherosclerosis.2016.11.010 (2017). 27998826 10.1016/j.atherosclerosis.2016.11.010 PMC5321848

[59] McEvoy, J. W. et al. INTERASPIRE: an international survey of coronary Patients; their Cardiometabolic, renal and biomarker Status; and the quality of preventive care delivered in all WHO regions: in partnership with the world heart Federation, European society of cardiology, Asia Pacific society of cardiology, interAmerican society of cardiology, and PanAfrican society of cardiology. *Curr. Cardiol. Rep.* **23**, 136. 10.1007/s11886-021-01568-2 (2021). 34410520 10.1007/s11886-021-01568-2 PMC8374115

[60] Chan, D. & Ng, L. L. Biomarkers in acute myocardial infarction. *BMC Med.* **8**, 34. 10.1186/1741-7015-8-34 (2010). 20529285 10.1186/1741-7015-8-34 PMC2898678

[61] Klimontov, V. V., Koroleva, E. A., Khapaev, R. S., Korbut, A. I. & Lykov, A. P. Carotid artery disease in subjects with type 2 diabetes: risk factors and biomarkers. *J. Clin. Med.* **11**10.3390/jcm11010072 (2021). 10.3390/jcm11010072 PMC8745306 35011813

[62] Selvin, E., Wattanakit, K., Steffes, M. W., Coresh, J. & Sharrett, A. R. HbA1c and peripheral arterial disease in diabetes: the atherosclerosis risk in communities study. *Diabetes Care*. **29**, 877–882. 10.2337/diacare.29.04.06.dc05-2018 (2006). 16567831 10.2337/diacare.29.04.06.dc05-2018

[63] Grübler, M. R. et al. Vitamin-D concentrations, cardiovascular risk and events - a review of epidemiological evidence. *Reviews Endocr. Metabolic Disorders*. **18**, 259–272. 10.1007/s11154-017-9417-0 (2017). 10.1007/s11154-017-9417-0 28451877

[64] Marcinowska-Suchowierska, E., Kupisz-Urbanska, M., Lukaszkiewicz, J., Pludowski, P. & Jones, G. Vitamin D Toxicity-A clinical perspective. *Front. Endocrinol. (Lausanne)*. **9**, 550. 10.3389/fendo.2018.00550 (2018). 30294301 10.3389/fendo.2018.00550 PMC6158375

[65] Wang, Y. et al. Association between Cystatin C and the risk of ischemic stroke: a systematic review and Meta-analysis. *J. Mol. Neurosci.* **69**, 444–449. 10.1007/s12031-019-01373-1 (2019). 31313057 10.1007/s12031-019-01373-1

[66] You, S. et al. Prognostic significance of international normalised ratio and prothrombin time in Chinese acute ischaemic stroke patients. *Postgrad. Med. J.*10.1136/postgradmedj-2021-141204 (2022). 37076768 10.1136/postmj/postgradmedj-2021-141204

[67] Kim, H. N., Eun, Y. M. & Song, S. W. Serum folate and vitamin B(12) levels are not associated with the incidence risk of atherosclerotic events over 12 years: the Korean genome and epidemiology study. *Nutr. Res.* **63**, 34–41. 10.1016/j.nutres.2018.12.009 (2019). 30824395 10.1016/j.nutres.2018.12.009

[68] Xiang, Y. et al. Atheroprotective mechanism by which folic acid regulates monocyte subsets and function through DNA methylation. *Clin. Epigenetics*. **14**, 32. 10.1186/s13148-022-01248-0 (2022). 35227297 10.1186/s13148-022-01248-0 PMC8887029

[69] Ngamdu, K. S. et al. Long-term Statin therapy is associated with severe coronary artery calcification. *PLoS One*. **18**, e0289111. 10.1371/journal.pone.0289111 (2023). 37498869 10.1371/journal.pone.0289111 PMC10374064

[70] Zhu, L. et al. Effect of an increase in Lp(a) following Statin therapy on cardiovascular prognosis in secondary prevention population of coronary artery disease. *BMC Cardiovasc. Disord*. **22**, 474. 10.1186/s12872-022-02932-y (2022). 36348286 10.1186/s12872-022-02932-y PMC9644478

[71] Ridker, P. M. Clinical application of C-reactive protein for cardiovascular disease detection and prevention. *Circulation* **107**, 363–369. 10.1161/01.cir.0000053730.47739.3c (2003). 12551853 10.1161/01.cir.0000053730.47739.3c

[72] Damen, J. A. et al. Prediction models for cardiovascular disease risk in the general population: systematic review. *Bmj* **353** (i2416). 10.1136/bmj.i2416 (2016). 10.1136/bmj.i2416 PMC4868251 27184143

[73] Kakileti, S. T. et al. Personalized risk prediction for breast cancer pre-screening using artificial intelligence and thermal radiomics. *Artif. Intell. Med.* **105**, 101854. 10.1016/j.artmed.2020.101854 (2020). 32505418 10.1016/j.artmed.2020.101854

[74] Khedher, L. et al. Early diagnosis of Alzheimer׳ s disease based on partial least squares, principal component analysis and support vector machine using segmented MRI images. *Neurocomputing* **151**, 139–150 (2015).

[75] Hashir, M. & Sawhney, R. Towards unstructured mortality prediction with free-text clinical notes. *J. Biomed. Inf.* **108**, 103489. 10.1016/j.jbi.2020.103489 (2020). 10.1016/j.jbi.2020.103489 32592755

[76] Ambale-Venkatesh, B. et al. Cardiovascular event prediction by machine learning: the Multi-Ethnic study of atherosclerosis. *Circ. Res.* **121**, 1092–1101. 10.1161/circresaha.117.311312 (2017). 28794054 10.1161/CIRCRESAHA.117.311312 PMC5640485

[77] Larrazabal, A. J., Nieto, N., Peterson, V., Milone, D. H. & Ferrante, E. Gender imbalance in medical imaging datasets produces biased classifiers for computer-aided diagnosis. *Proc. Natl. Acad. Sci. U S A*. **117**, 12592–12594. 10.1073/pnas.1919012117 (2020). 32457147 10.1073/pnas.1919012117 PMC7293650

[78] Kilic, A. Artificial intelligence and machine learning in cardiovascular health care. *Ann. Thorac. Surg.* **109**, 1323–1329. 10.1016/j.athoracsur.2019.09.042 (2020). 31706869 10.1016/j.athoracsur.2019.09.042

[79] Brindle, P. et al. Predictive accuracy of the Framingham coronary risk score in British men: prospective cohort study. *Bmj* **327**, 1267. 10.1136/bmj.327.7426.1267 (2003). 14644971 10.1136/bmj.327.7426.1267 PMC286248

[80] Melamed, M. L., Michos, E. D., Post, W. & Astor, B. 25-hydroxyvitamin D levels and the risk of mortality in the general population. *Arch. Intern. Med.* **168**, 1629–1637. 10.1001/archinte.168.15.1629 (2008). 18695076 10.1001/archinte.168.15.1629 PMC2677029

[81] Di Angelantonio, E. et al. Lipid-related markers and cardiovascular disease prediction. *Jama* **307**, 2499–2506. 10.1001/jama.2012.6571 (2012). 22797450 10.1001/jama.2012.6571 PMC4211641

[82] Finlayson, S. G. et al. The clinician and dataset shift in artificial intelligence. *N Engl. J. Med.* **385**, 283–286. 10.1056/NEJMc2104626 (2021). 34260843 10.1056/NEJMc2104626 PMC8665481

[83] Rotalinti, Y., Tucker, A., Lonergan, M., Myles, P. & Branson, R. 243–258 (Springer Nature Switzerland).

[84] Tirkkonen, A. et al. Predicting cardiovascular morbidity and mortality with SCORE2 (OP) and Framingham risk estimates in combination with indicators of biological ageing. *Age Ageing*. **54**10.1093/ageing/afaf075 (2025). 10.1093/ageing/afaf075 PMC11966606 40178198

