# Supplementary material for: Cardiovascular risk assessment enhanced by automated machine learning in a multi-phase study
Source: Sci Rep. 2025 Oct 20;15:36474. doi: 10.1038/s41598-025-24189-z (PMC12537956; doi:10.1038/s41598-025-24189-z)
Supplement: Supplementary file 1 — Supplementary Material 1 [file 41598_2025_24189_MOESM1_ESM.pdf]

Data Study Handling Overview

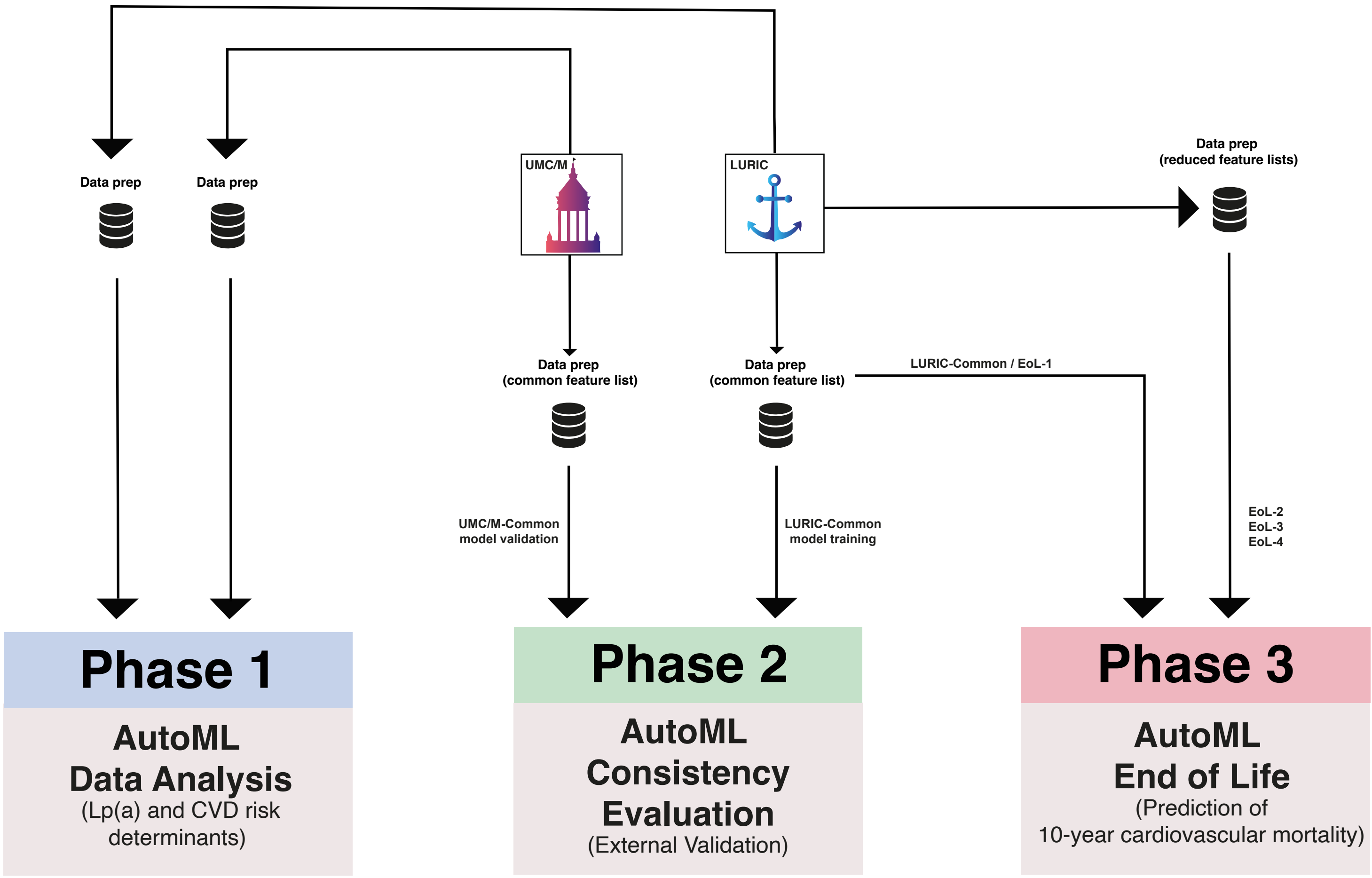

Figure S1

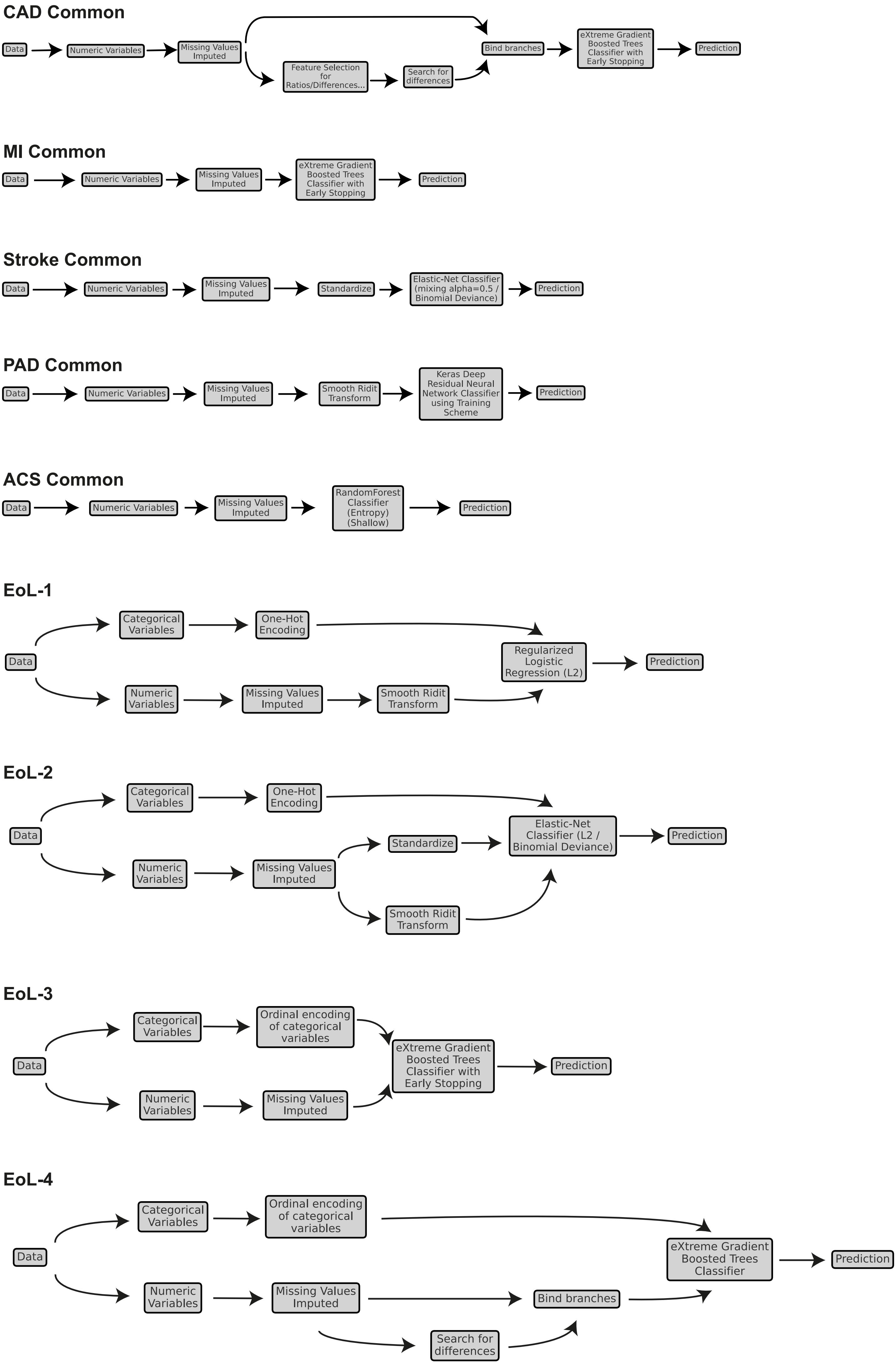
CAD Common
Data
Numeric Variables
Missing Values Imputed
Feature Selection for Ratios/Differences...
Search for differences
Bind branches
eXtreme Gradient Boosted Trees Classifier with Early Stopping
Prediction
MI Common
Data
Numeric Variables
Missing Values Imputed
eXtreme Gradient Boosted Trees Classifier with Early Stopping
Prediction
Stroke Common
Data
Numeric Variables
Missing Values Imputed
Standardize
Elastic-Net Classifier (mixing alpha=0.5 / Binomial Deviance)
Prediction
PAD Common
Data
Numeric Variables
Missing Values Imputed
Smooth Ridit Transform
Keras Deep Residual Neural Network Classifier using Training Scheme
Prediction
ACS Common
Data
Numeric Variables
Missing Values Imputed
RandomForest Classifier (Entropy) (Shallow)
Prediction
EoL-1
Data
Categorical Variables
One-Hot Encoding
Numeric Variables
Missing Values Imputed
Smooth Ridit Transform
Regularized Logistic Regression (L2)
Prediction
EoL-2
Data
Categorical Variables
One-Hot Encoding
Numeric Variables
Missing Values Imputed
Standardize
Smooth Ridit Transform
Elastic-Net Classifier (L2 / Binomial Deviance)
Prediction
EoL-3
Data
Categorical Variables
Ordinal encoding of categorical variables
Numeric Variables
Missing Values Imputed
eXtreme Gradient Boosted Trees Classifier with Early Stopping
Prediction
EoL-4
Data
Categorical Variables
Ordinal encoding of categorical variables
Numeric Variables
Missing Values Imputed
Bind branches
Search for differences
eXtreme Gradient Boosted Trees Classifier
Prediction

Figure S2

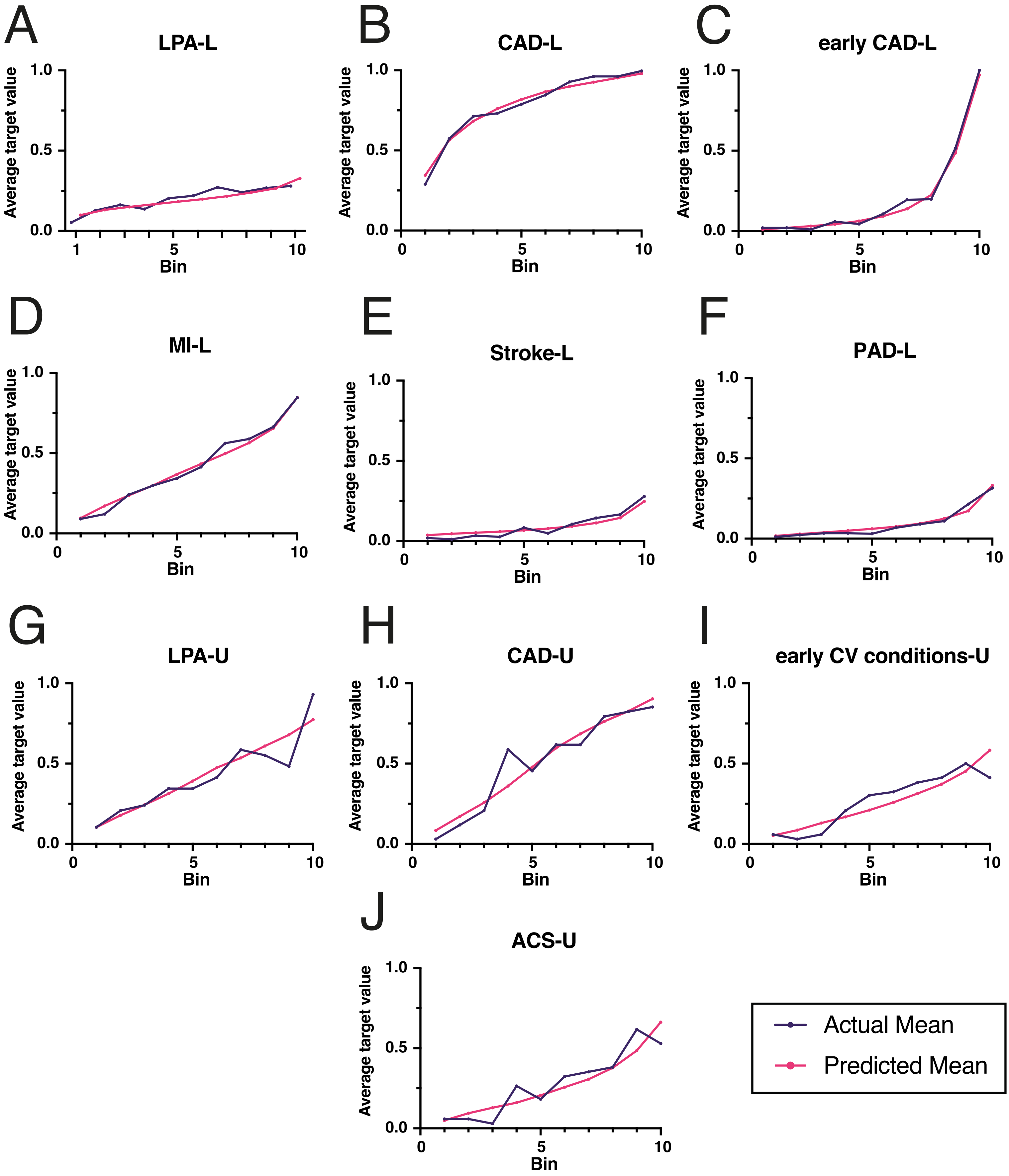
A
LPA-L
B
CAD-L
C
early CAD-L
D
MI-L
E
Stroke-L
F
PAD-L
G
LPA-U
H
CAD-U
I
early CV conditions-U
J
ACS-U
Average target value
Bin
Actual Mean
Predicted Mean

Figure S3

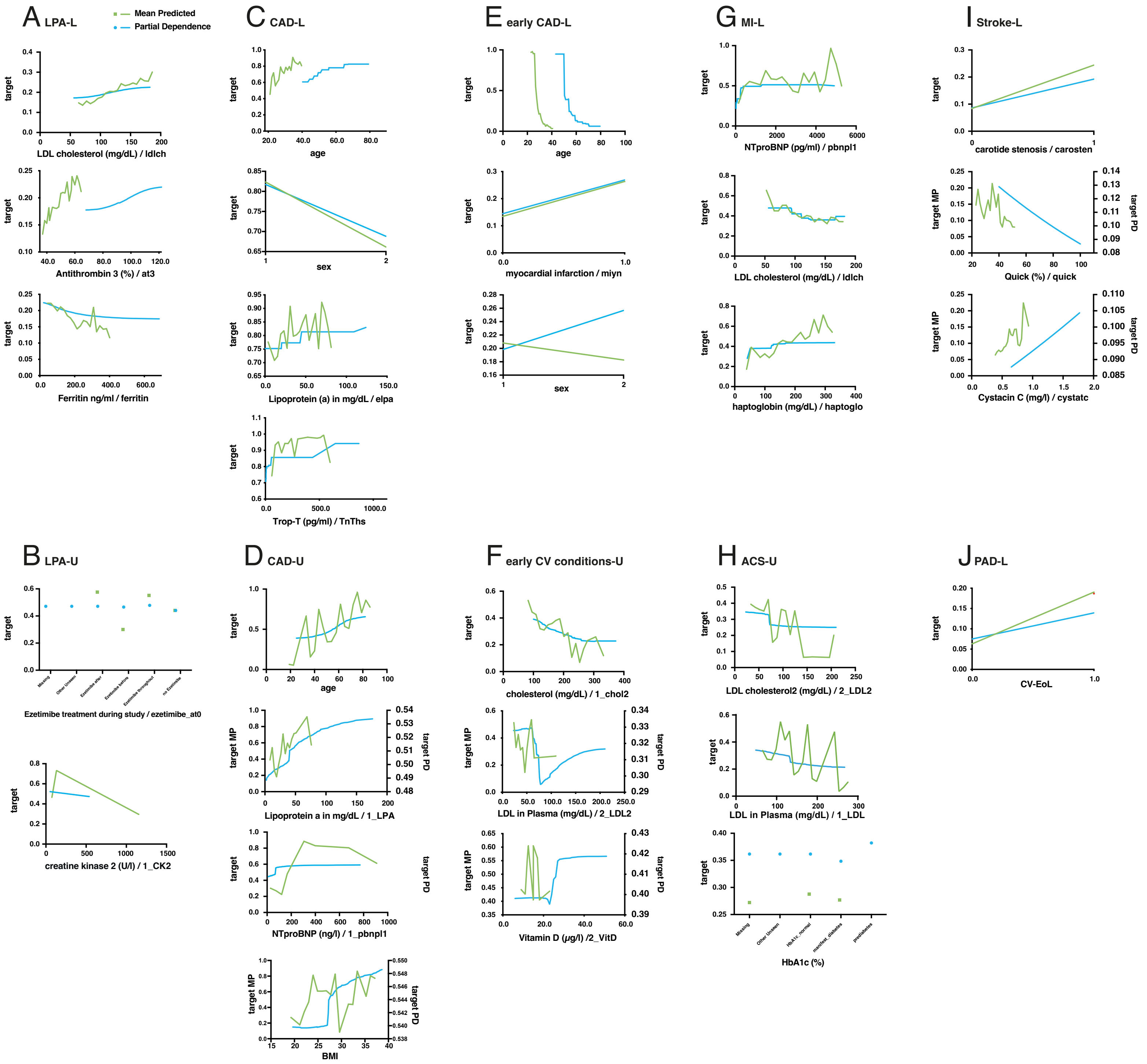
A LPA-L
Mean Predicted
Partial Dependence
LDL cholesterol (mg/dL) / ldlch
Antithrombin 3 (%) / at3
Ferritin ng/ml / ferritin
C CAD-L
age
sex
Lipoprotein (a) in mg/dL / elpa
Trop-T (pg/ml) / TnThs
E early CAD-L
age
myocardial infarction / miyn
sex
G MI-L
NTproBNP (pg/ml) / pbnpl1
LDL cholesterol (mg/dL) / ldlch
haptoglobin (mg/dL) / haptoglo
I Stroke-L
carotide stenosis / carosten
Quick (%) / quick
Cystacin C (mg/l) / cystatc
B LPA-U
Missing
Other Unseen
Ezetimibe after
Ezetimibe before
Ezetimibe throughout
no Ezetimibe
Ezetimibe treatment during study / ezetimibe_at0
creatine kinase 2 (U/l) / 1_CK2
D CAD-U
age
Lipoprotein a in mg/dL / 1_LPA
NTproBNP (ng/l) / 1_pbnpl1
BMI
F early CV conditions-U
cholesterol (mg/dL) / 1_chol2
LDL in Plasma (mg/dL) / 2_LDL2
Vitamin D (µg/l) /2_VitD
H ACS-U
LDL cholesterol2 (mg/dL) / 2_LDL2
LDL in Plasma (mg/dL) / 1_LDL
Missing
Other Unseen
HbA1c_normal
manifest_diabetes
prediabetes
HbA1c (%)
J PAD-L
CV-EoL
target
target MP
target PD

Figure S4

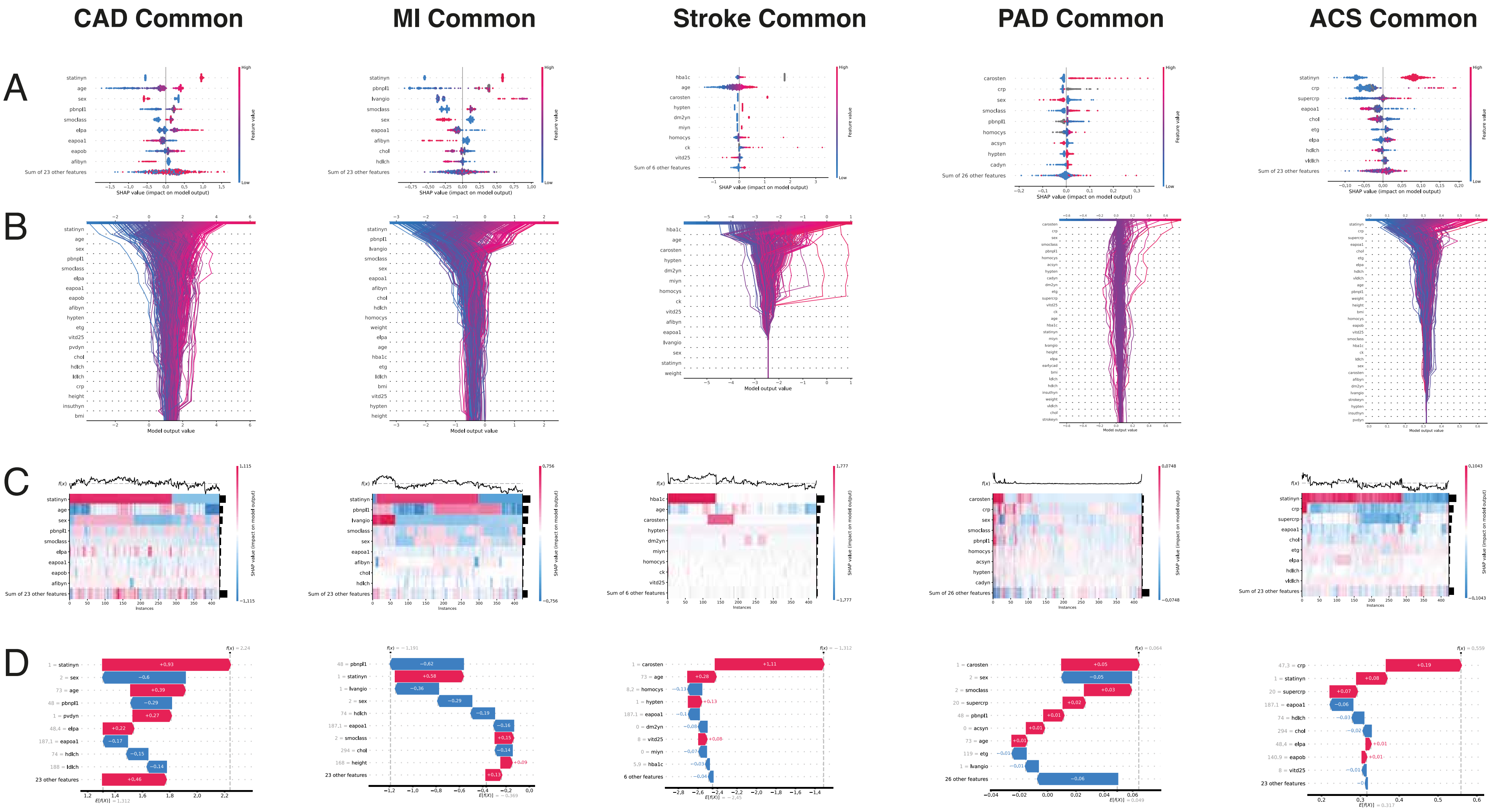
CAD Common
MI Common
Stroke Common
PAD Common
ACS Common
A
B
C
D

Figure S5

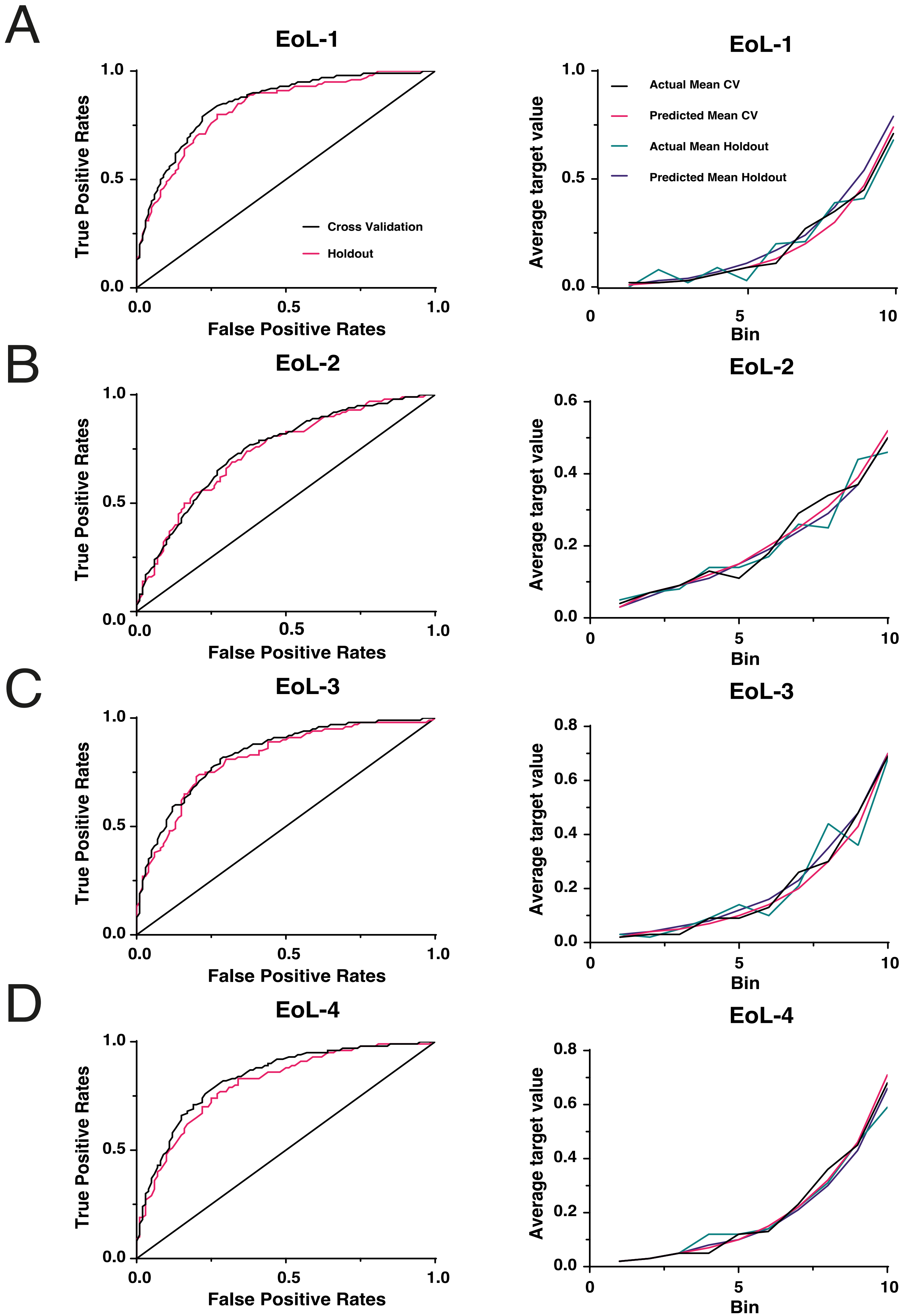
A
EoL-1
True Positive Rates
False Positive Rates
Cross Validation
Holdout
EoL-1
Average target value
Bin
Actual Mean CV
Predicted Mean CV
Actual Mean Holdout
Predicted Mean Holdout
B
EoL-2
True Positive Rates
False Positive Rates
EoL-2
Average target value
Bin
C
EoL-3
True Positive Rates
False Positive Rates
EoL-3
Average target value
Bin
D
EoL-4
True Positive Rates
False Positive Rates
EoL-4
Average target value
Bin

Figure S6

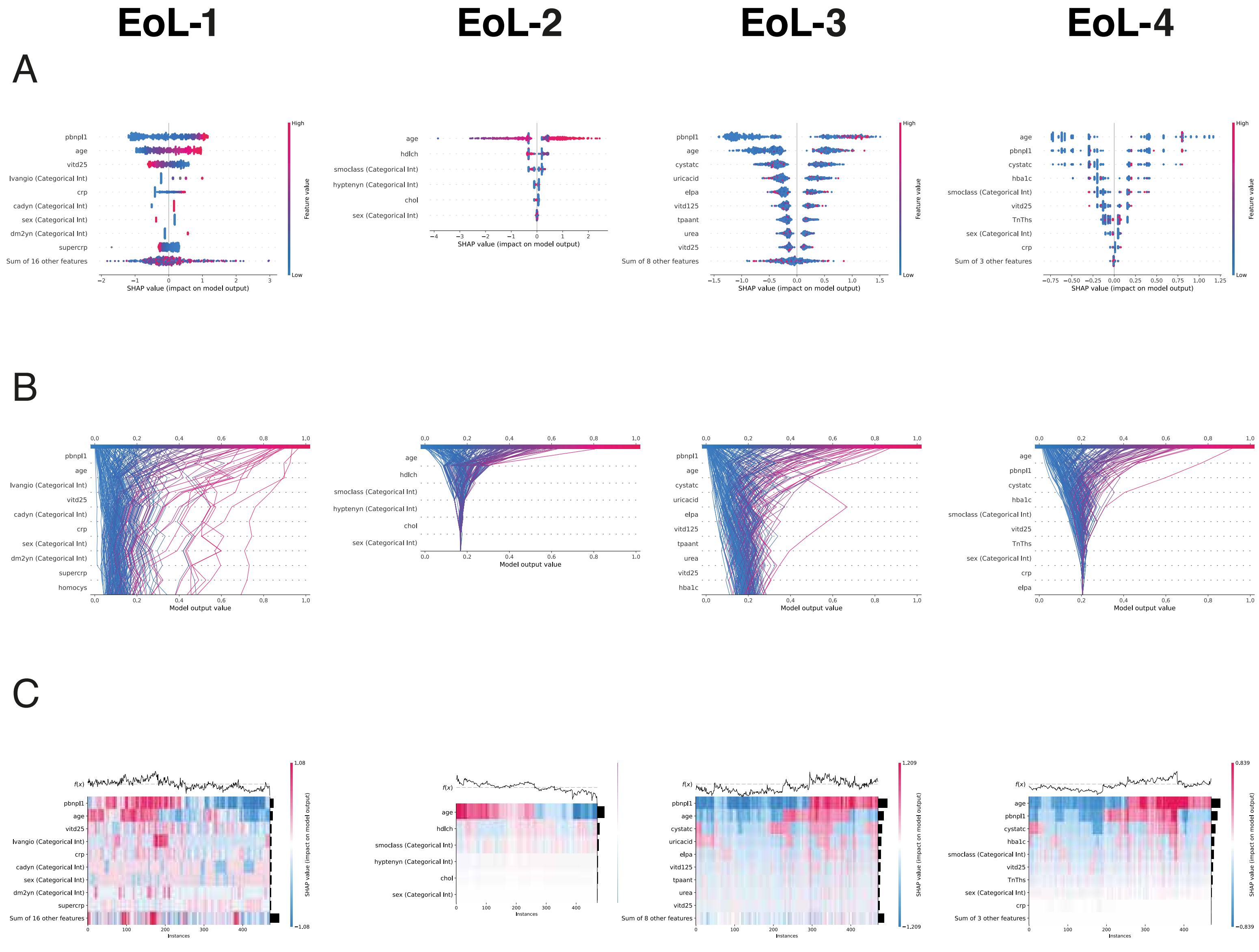
EoL-1
EoL-2
EoL-3
EoL-4
A
B
C
SHAP value (impact on model output)
Feature value
High
Low
Model output value
Instances
SHAP value (impact on model output)
pbnpl1
age
vitd25
Ivangio (Categorical Int)
crp
cadyn (Categorical Int)
sex (Categorical Int)
dm2yn (Categorical Int)
supercrp
homocys
Sum of 16 other features
hdlch
smoclass (Categorical Int)
hyptenyn (Categorical Int)
chol
cystatc
uricacid
elpa
vitd125
tpaant
urea
hba1c
TnThs
Sum of 8 other features
Sum of 3 other features
f(x)
1.08
−1.08
1.209
−1.209
0.839
−0.839

Figure S7
