# Supplementary material for: Cardiovascular risk assessment enhanced by automated machine learning in a multi-phase study
Source: Sci Rep. 2025 Oct 20;15:36474. doi: 10.1038/s41598-025-24189-z (PMC12537956; doi:10.1038/s41598-025-24189-z)
Supplement: Supplementary file 3 — Supplementary Material 3 [file 41598_2025_24189_MOESM3_ESM.pdf]

Feature name	Type	Method
**Any CAD (coronary artery disease)**	binary	Transformation of single or multi-vessel coronary artery disease classification into a single feature<br>0 (no)<br>1 (yes)
**BMI categorical**	multiclass	Classification of BMI values according to the following classes:<br><br>Underweight (BMI below 17.5)<br>Normal weight (BMI 17.5-24.9)<br>Overweight (BMI 25-29.9)<br>Obese (BMI over 29.9)
**Early cardiovascular conditions**	binary	Merging of the following two features in the primary dataset into one:<br><br>Early cardiovascular disease<br>Early chronic venous insufficiency<br>Peripheral artery disease
**CHA2DS2VASC Score**	numeric	Calculation of new feature from retrospective data with the help of the following features:<br><br>Congestive heart failure<br>Age over 75 years<br>Diabetes<br>Stroke<br>Vascular disease<br>Age 65-74<br>Sex (female)
**Ezetimibe**	multiclass	Classification of Ezetimibe intake over the course of the study into the following classes:<br><br>No Ezetimibe<br>Ezetimibe before study enrolment<br>Ezetimibe after study enrolment
**HbA1c categorical (at enrolment and during the study)**	multiclass	Classification of HbA1c laboratory results into the following classes:<br><br>Normal HbA1c levels<br>Prediabetes<br>Manifest Diabetes
**HbA1c development**	multiclass	Classification of HbA1c categories from first to second study visit into the following classes:<br><br>No HbA1c change<br>HbA1c increase<br>HbA1c decrease

**LDL categorical**	multiclass	Categorization of LDL values into the following classes:<br><br>Normal LDL (below 100mg/dL)<br>Near optimal LDL (100-129mg/dL)<br>Borderline high LDL (130-159mg/dL)<br>High LDL (160-189mg/dL)<br>Very high LDL (over 189mg/dL)
**LDL categorical binary**	binary	LDL categories were assigned as follows:<br><br>0. LDL normal, near optimal and borderline high<br>1. LDL high or very high
**Lipoprotein (a) over 50mg/dL**	binary	Lipoprotein (a) values were assigned as either 0 or 1 with a cut-off of 50mg/dL in both the UMC/M and LURIC datasets.
