# Supplementary material for: Cardiovascular risk assessment enhanced by automated machine learning in a multi-phase study
Source: Sci Rep. 2025 Oct 20;15:36474. doi: 10.1038/s41598-025-24189-z (PMC12537956; doi:10.1038/s41598-025-24189-z)
Supplement: Supplementary file 4 — Supplementary Material 4 [file 41598_2025_24189_MOESM4_ESM.pdf]

Study Phase	Phase 2		Phase 3			
**Feature List Name**	**LURIC-Common**	**UMC/M-Common**	**LURIC-Common/EoL-1**	**EoL-2**	**EoL-3**	**EoL-4**
**Target Group/Name**	**Specific CVDs**		**EoL-1**	**EoL-2**	**EoL-3**	**EoL-4**
**Final Features before EDA (* indicates features retained after EDA)**	sex*	sex	sex	hdlchol	sex	sex
	**cadyn**	**cadyn**	cadyn	hypten	urea	hba1c
	**strokeyn***	**strokeyn**	strokeyn	chol	uricacid	vitd25
	carosten*	carosten	carosten	sex	iron	vitd125
	**pvdyn**	**pvdyn**	pvdyn	smoke	ferritin	crp
	dm1yn	dm1	dm1yn	age	hba1c	elpa
	dm2yn*	dm2	dm2yn	**CV-EoL**	ace	cystatc
	insuthyn	insulin_pt0	insuthyn		vitb12	age
	afibyn*	afibyn	afibyn		folicac	smoclass
	height	height	height		vitd25	pbnpl1
	weight*	weight	weight		vitd125	TnThs
	chol	chol	chol		fibrinog	Galectin3
	ck*	CK	ck		at3	**CV-EoL**
	hba1c*	HbA1c	hba1c		fii	
	homocys*	homocys	homocys		vwfag	
	vitd25*	vitD	vitd25		ddimer	
	crp	CRP	crp		tpaant	
	etg	TG	etg		crp	
	eapoa1*	ApoA1	eapoa1		haptoglo	
	eapob	ApoB	eapob		eapoa1	
	elpa	LPA	elpa		eapoa2	
	vldlch	VLDLprebetafract	vldlch		eapob	
	ldlch	LDL_C	ldlch		eapoe	
	hdlch	HDL	hdlch		elpa	
	supercrp	hscrp	supercrp		cystatc	
	lvangio*	lvfunction	lvangio		age	
	age*	age	age		smoclass	
	bmi	bmi	bmi		pbnpl1	
	smoclass	smoke	smoclass		TnThs	
	**miyn***	**miyn**	miyn		Galectin3	
	**acsyn**	**acsyn**	acsyn		**CV-EoL**	
	earlycad	earlycvcondition	earlycad			
	pbnpl1	NPPB	pbnpl1			
	statinyn*	statinyn	statinyn			
	hypten*	aht	hypten			
	COPDyn	COPDyn	COPDyn			
			**CV-EoL**			
