# Supplementary material for: Cardiovascular risk assessment enhanced by automated machine learning in a multi-phase study
Source: Sci Rep. 2025 Oct 20;15:36474. doi: 10.1038/s41598-025-24189-z (PMC12537956; doi:10.1038/s41598-025-24189-z)
Supplement: Supplementary file 5 — Supplementary Material 5 [file 41598_2025_24189_MOESM5_ESM.pdf]

Target groups	Target	Built Models	Selected Model	AUC			LogLoss			Max MCC		
				Validation	CV	Holdout	Validation	CV	Holdout	Validation	CV	Holdout
**Lp(a)**	**LPA-L**	137	**Elastic-Net Classifier (L1 / Binomial Deviance)**	0.6084 (0.6083;0.6086)	**0.6249 (0.6248;0.6249)**	0.6065 (0.6064;0.6065)	0.2932 (0.2913; 0.295)	**0.266 (0.2644; 0.2676)**	0.2587 (0.2568; 0.2607)	0.6581 (0.6489; 0.6673)	**0.7129 (0.7096; 0.7162)**	0.6656 (0.6576; 0.6736)
**specific CVDs**	**CAD-L**	10	**eXtreme Gradient Boosted Trees Classifier**	0.8458 (0.8455;0.8461)	**0.8244 (0.8243;0.8245)**	0.8178 (0.8175;0.8180)	0.2610 (0.261; 0.2611)	**0.2752 (0.2751; 0.2752)**	0.2774 (0.2774; 0.2775)	0.2994 (0.2992; 0.2996)	**0.2712 (0.2705; 0.2718)**	0.2322 (0.2301; 0.2343)
	**early CAD-L**	120	**eXtreme Gradient Boosted Trees Classifier**	0.8932 (0.8908;0.8956)	**0.9101 (0.9088;0.9114)**	0.9105 (0.9089;0.9120)	0.5581 (0.5579; 0.5583)	**0.5548 (0.5547; 0.5549)**	0.5858 (0.5855; 0.5861)	0.4473 (0.4433; 0.4513)	**0.434 (0.4322; 0.4359)**	0.3623 (0.3612; 0.3634)
	**MI-L**	72	**eXtreme Gradient Boosted Trees Classifier**	0.7725 (0.7721;0.7730)	**0.7770 (0.7769;0.7771)**	0.7334 (0.7330;0.7337)	0.2744 (0.2718; 0.277)	**0.264 (0.2639; 0.264)**	0.2905 (0.2904; 0.2905)	0.3632 (0.3607; 0.3657)	**0.3353 (0.3336; 0.337)**	0.3344 (0.3329; 0.3359)
	**Stroke-L**	10	**Elastic-Net Classifier (mixing alpha=0.5 / Binomial Deviance)**	0.7989 (0.7986;0.7991)	**0.7505 (0.7503;0.7507)**	0.7337 (0.7334;0.7340)	0.3851 (0.385; 0.3852)	**0.4007 (0.4006; 0.4008)**	0.4061 (0.406; 0.4062)	0.5117 (0.5096; 0.5138)	**0.4685 (0.4677; 0.4694)**	0.4523 (0.4502; 0.4544)
	**PAD-L**	10	**Elastic-Net Classifier (mixing alpha=0.5 / Binomial Deviance)**	0.7738 (0.7736;0.7740)	**0.7823 (0.7822;0.7825)**	0.7940 (0.7938;0.7942)	0.261 (0.261; 0.2611)	**0.2752 (0.2751; 0.2752)**	0.2774 (0.2774; 0.2775)	0.2994 (0.2992; 0.2996)	**0.2712 (0.2705; 0.2718)**	0.2322 (0.2301; 0.2343)
**Lp(a)**	**LPA-U**	77	**Elastic-Net Classifier (L2 / Binomial Deviance)**	0.7031 (0.7028;0.7034)	**0.7409 (0.7406;0.7413)**	0.7679 (0.7676;0.7681)	0.6388 (0.6386; 0.639)	**0.5904 (0.5903; 0.5905)**	0.5507 (0.5506; 0.5507)	0.4322 (0.4322; 0.4322)	**0.4589 (0.4567; 0.461)**	0.4912 (0.4912; 0.4912)
**specific CVDs**	**CAD-U**	35	**Support Vector Classifier (Radial Kernel)**	0.8568 (0.8559;0.8577)	**0.8128 (0.8113;0.8143)**	0.8108 (0.8093;0.8123)	0.4718 (0.4695; 0.4741)	**0.533 (0.5309; 0.5351)**	0.5349 (0.5332; 0.5366)	0.6201 (0.6201; 0.6201)	**0.5614 (0.5583; 0.5645)**	0.5608 (0.5531; 0.5685)
	**early CV conditio ns-U**	280	**AVG Blender**	0.7222 (0.7074;0.7371)	**0.7003 (0.6895;0.7111)**	0.6376 (0.6236;0.6517)	0.5478 (0.5065; 0.5891)	**0.5753 (0.541; 0.6096)**	0.6093 (0.5789; 0.6397)	0.391 (0.3785; 0.4035)	**0.3778 (0.3658; 0.3899)**	0.297 (0.2731; 0.3209)
	**ACS-U**	71	**Keras Deep Residual Neural Network Classifier using Training Schedule (3 Layers: 512, 64, 64 Units)**	0.8158 (0.8018;0.8298)	**0.7591 (0.7512;0.7669)**	0.7315 (0.7031;0.7598)	0.4758 (0.4669; 0.4848)	**0.5202 (0.5158; 0.5246)**	0.53 (0.5161; 0.544)	0.5023 (0.4746; 0.5299)	**0.4536 (0.4361; 0.471)**	0.4109 (0.3766; 0.4453)

Target Group	Target	Com-pared Models	Selected Model	AUC				LogLoss				Max MCC			
				Validation	CV	Holdout	Secondary dataset validation	Validation	CV	Holdout	Secondary dataset validation	Validation	CV	Holdout	Secondary dataset validation
Specific CVDs	CAD Common	39	eXtreme Gradient Boosted Trees Classifier with Early Stopping	0.8728 (0.8707;0.8749)	**0.8417 (0.8407;0.8427)**	0.8358 (0.8345;0.8371)	0.7757 (0.7741;0.7773)	0.3591 (0.3566;0.3615)	0.3866 (0.3854;0.3878)	0.3897 (0.3885;0.3908)	0.7746 (0.7692;0.7801)	0.5546 (0.5507;0.5585)	0.5071 (0.5044;0.5098)	0.4816 (0.4753;0.4879)	0.4371 (0.4294;0.4448)
	MI Common	39	eXtreme Gradient Boosted Trees Classifier with Early Stopping	0.8005 (0.7999;0.8011)	**0.8048 (0.8036;0.8059)**	0.8097 (0.8088;0.8106)	0.8080 (0.8063;0.8098)	0.5332 (0.5322;0.5341)	0.5279 (0.5271;0.5287)	0.5200 (0.5189;0.5211)	0.4879 (0.4857;0.4900)	0.4600 (0.4562;0.4638)	0.4762 (0.4741;0.4783)	0.4845 (0.4807;0.4883)	0.4362 (0.4289;0.4436)
	Stroke Common	39	Elastic-Net Classifier (mixing alpha=0.5 / Binomial Deviance)	0.7707 (0.7703;0.7711)	**0.7486 (0.7484;0.7487)**	0.6842 (0.6839;0.6845)	0.6465 (0.6459;0.6470)	0.2743 (0.2742;0.2744)	0.2759 (0.2759;0.2760)	0.2854 (0.2853;0.2854)	0.2875 (0.2870;0.2880)	0.2526 (0.2519;0.2533)	0.2691 (0.2685;0.2697)	0.1921 (0.1903;0.1938)	0.1666 (0.1660;0.1672)
	PAD Common	39	Keras Deep Residual Neural Network Classifier using Training Schedule (3 Layers: 512, 64, 64 Units)	0.7555 (0.7550;0.7560)	**0.7584 (0.7580;0.7588)**	0.7649 (0.7645;0.7653)	0.7870 (0.7860;0.7880)	0.2797 (0.2792;0.2802)	0.2813 (0.2808;0.2818)	0.2725 (0.2720;0.2730)	0.3663 (0.3653;0.3673)	0.2941 (0.2936;0.2946)	0.2879 (0.2874;0.2884)	0.3239 (0.3234;0.3244)	0.3234 (0.3229;0.3239)
	ACS Common	42	RandomForest Classifier (Entropy) (Shallow)	0.6934 (0.6922;0.6945)	**0.7224 (0.7221;0.7227)**	0.7108 (0.7097;0.712)	0.7273 (0.7252;0.7294)	0.5496 (0.5491;0.5502)	0.5443 (0.5441;0.5445)	0.5587 (0.5580;0.5593)	0.5385 (0.5376;0.5395)	0.3329 (0.3290;0.3367)	0.3656 (0.3636;0.3675)	0.3214 (0.3160;0.3268)	0.3652 (0.3602;0.3701)

Target group	Target	Compared Models	Selected Model	AUC			LogLoss			Max MCC		
				Validation	CV	Holdout	Validation	CV	Holdout	Validation	CV	Holdout
EoL	**EoL-1**	78	**Regularized Logistic Regression (L2)**	0.8163 (0.8163;0.8163)	**0.8491 (0.8491;0.8491)**	0.8274 (0.8274;0.8274)	0.3953 (0.3953;0.3953)	**0.3718 (0.3718;0.3718)**	0.3924 (0.3924;0.3924)	0.4724 (0.4724;0.4724)	**0.5102 (0.5102;0.5102)**	0.4541 (0.4541;0.4541)
	**EoL-2**	38	**Elastic-Net Classifier (L2 / Binomial Deviance)**	0.7299 (0.7263;0.7299)	**0.7450 (0.7428;0.7450)**	0.7363 (0.7357;0.7389)	0.4559 (0.4559;0.4576)	**0.4530 (0.4530;0.4543)**	0.4496 (0.4489;0.4498)	0.3153 (0.3153;0.3261)	**0.3473 (0.3453;0.3506)**	0.3281 (0.3250;0.3419)
	**EoL-3**	60	**eXtreme Gradient Boosted Trees Classifier with Early Stopping**	0.8163 (0.8094;0.8163)	**0.8340 (0.8312;0.8352)**	0.8157 (0.8139;0.8192)	0.4047 (0.4039;0.4092)	**0.3857 (0.3853;0.3891)**	0.3984 (0.3951;0.4000)	0.4315 (0.4171;0.4382)	**0.4754 (0.4681;0.4874)**	0.4667 (0.4470;0.4744)
	**EoL-4**	38	**eXtreme Gradient Boosted Trees Classifier**	0.8053 (0.8053;0.8053)	**0.8339 (0.8339;0.8339)**	0.8062 (0.8062;0.8062)	0.4011 (0.4011;0.4011)	**0.3865 (0.3865;0.3865)**	0.4014 (0.4014;0.4014)	0.4340 (0.4340;0.4340)	**0.4828 (0.4828;0.4828)**	0.4183 (0.4183;0.4183)
