# Supplementary material for: Cardiovascular risk assessment enhanced by automated machine learning in a multi-phase study
Source: Sci Rep. 2025 Oct 20;15:36474. doi: 10.1038/s41598-025-24189-z (PMC12537956; doi:10.1038/s41598-025-24189-z)
Supplement: Supplementary file 6 — Supplementary Material 6 [file 41598_2025_24189_MOESM6_ESM.pdf]

LPA-L	CAD-L	early CAD-L		MI-L	Stroke-L		PAD-L
sex	**cadyn**	**earlycad**	eapoe	**miyn**	sex	eapoe	**padyn**
cadyn	sex	sex	elpa	sex	cadyn	elpa	sex
vdyn	strokeyn	cadyn	vldlch	crea	**strokeyn**	vldlch	cadyn
exercise	carosten	strokeyn	vldltg	urea	carosten	vldltg	strokeyn
urea	pvdyn	carosten	ldlch	uricacid	pvdyn	ldlch	carosten
chol	dm1yn	pvdyn	ldltg	ldlchol	dm1yn	ldltg	pvdyn
ferritin	dm2yn	dm1yn	supercrp	iron	dm2yn	supercrp	dm1yn
vitb12	canceryn	dm2yn	cystatc	ferritin	canceryn	cystatc	dm2yn
at3	venthrom	canceryn	age	hba1c	venthrom	age	canceryn
fii	infectyn	venthrom	bmi	ace	infectyn	bmi	venthrom
vwfag	cmpyn	infectyn	ishypert	vitb12	cmpyn	ishypert	infectyn
eapoa1	vdyn	cmpyn	smoclass	folicac	vdyn	smoclass	cmpyn
eapoa2	rhythyn	vdyn	miyn	tsh	rhythyn	miyn	vdyn
eapob	immunyn	rhythyn	pbnpl1	vitd25	immunyn	pbnpl1	rhythyn
**lpa50**	anginayn	immunyn	CV-EoL	parat	anginayn	CV-EoL	immunyn
vldlch	dyspneyn	anginayn	TnThs	hb	dyspneyn	TnThs	anginayn
vldltg	exercise	dyspneyn	Galectin3	quick	exercise	Galectin3	dyspneyn
ldlch	crea	exercise		fibrinog	crea		exercise
miyn	urea	crea		at3	urea		crea
Galectin3	uricacid	urea		fii	uricacid		urea
	ldlchol	uricacid		vwfag	ldlchol		uricacid
	iron	ldlchol		ddimer	iron		ldlchol
	ferritin	iron		tpaant	ferritin		iron
	ldh	ferritin		crp	ldh		ferritin
	hba1c	ldh		haptoglo	hba1c		ldh
	ace	hba1c		eapoa1	ace		hba1c
	vitb12	ace		eapoa2	vitb12		ace
	folicac	vitb12		eapob	folicac		vitb12
	tsh	folicac		eapoe	tsh		folicac
	vitd25	tsh		elpa	vitd25		tsh
	parat	vitd25		vldlch	parat		vitd25
	hb	parat		vldltg	hb		parat
	quick	hb		ldlch	quick		hb
	aptt	quick		ldltg	aptt		quick
	fibrinog	aptt		supercrp	fibrinog		aptt
	at3	fibrinog		cystatc	at3		fibrinog
	fii	at3		age	fii		at3
	vwfag	fii		bmi	vwfag		fii
	ddimer	vwfag		smoclass	ddimer		vwfag
	tpaant	ddimer		pbnpl1	tpaant		ddimer
	crp	tpaant		TnThs	crp		tpaant
	haptoglo	crp		Galectin3	haptoglo		crp
	eapoa1	haptoglo			eapoa1		haptoglo
	eapoa2	eapoa1			eapoa2		eapoa1
	eapob	eapoa2			eapob		eapoa2
	eapoe	eapob					eapob
	elpa						eapoe
