# Supplementary material for: Cardiovascular risk assessment enhanced by automated machine learning in a multi-phase study
Source: Sci Rep. 2025 Oct 20;15:36474. doi: 10.1038/s41598-025-24189-z (PMC12537956; doi:10.1038/s41598-025-24189-z)
Supplement: Supplementary file 7 — Supplementary Material 7 [file 41598_2025_24189_MOESM7_ESM.pdf]

LPA-U	CAD-U	early CV conditions-U				ACS-U
sex	**cadyn**	**earlycvevent**	2_LPA	4_HDL	6_LDLbetafract	**acsyn**
BMI cat	sex	1_LDLbetafract	2_ApoA1	4_chol	6_HDLalphafract	sex
statin_pt0	age	1_HDLalphafract	2_ApoB	4_TG	6_VDLprebetafract	age
statin_at0	BMI	1_VDLprebetafract	2_CRP	4_LDL2	6_blipo mg/	BMI
ezetimibe	HbA1c	1_blipo	2_regCRP	4_HDL2	6_alipo	HbA1c cat
ezetimibe_pt0	HbA1c_at0	1_alipo	2_NPPB	4_chol2	6_preblipo	HbA1c_at0 cat
ezetimibe_at0	1_LDL	1_prebfract	2_NPPB_GFR	4_TG2	6_a_lipo2	pos_ FA
PSCK9	1_HDL	1_alipo2	2_homocys	4_CK	6_b_lipo2	smoke
otherlipidpharm	1_chol	1_b_lipo2	2_vitD	4_CK2	6_LDL	1_LDL
bypass_surgery	1_TG	1_LDL	3_LDLbetafract	4_LPA	6_HDL	1_HDL
COPD	1_CK	1_HDL	3_HDLalphafract	4_ApoA1	6_chol	1_chol
LVF	1_LPA	1_chol	3_VDLprebetafract	4_ApoB	6_TG	1_TG
LVFhireduced	1_ApoA1	1_TG	3_blipo mg/	4_CRP	6_LDLiPl2	1_LDL2
ddysfct	1_ApoB	1_LDL2	3_alipo	4_regCRP	6_HDL2	1_HDL2
aht	1_CRP	1_HDL2	3_preblipo	4_NPPB	6_chol2	1_chol2
dm1	1_NPPB	1_chol2	3_a_lipo2	4_NPPB_GFR	6_TG2	1_TG2
HbA1c cat	1_homocys	1_TG2	3_b_lipo2	4_homocys	6_CK	1_CK
pos_ FA	1_vitD	1_CK	3_LDL	4_vitD	6_CK2	1_CK2
earlycvevent	2_LDL2	1_CK2	3_HDL	5_LDLbetafract	6_LPA	1_LPA
LDL_C	2_HDL2	1_LPA	3_chol	5_HDLalphafract	6_ApoA1	1_ApoA1
FHscore	2_chol2	1_ApoA1	3_TG	5_VDLprebetafract	6_ApoB	1_ApoB
CHA2DS2_VASc_Score	2_TG2	1_ApoB	3_LDL2	5_blipo mg/	6_CRP	1_CRP
SGLT2pt0	2_CK	1_CRP	3_HDL2	5_alipo	6_regCRP	1_NPPB
SGLT2at0	2_LPA	1_regCRP	3_chol2	5_preblipo	6_NPPB	1_homocys
1_alipo2	2_ApoA1	1_NPPB	3_TG2	5_a_lipo2	6_NPPB_GFR	1_vitD
1_b_lipo2	2_ApoB	1_NPPB_GFR	3_CK	5_b_lipo2	6_homocys	2_LDL
1_LDL	2_CRP	1_homocys	3_CK2	5_LDL	6_vitD	2_HDL
1_LDL_categorical	2_NPPB	1_vitD	3_LPA	5_HDL		2_chol
LDLcatyn	2_homocys	2_LDLbetafract	3_ApoA1	5_chol		2_TG
1_HDL	2_vitD	2_HDLalphafract	3_ApoB	5_TG		2_LDL2
1_TG2		2_VDLprebetafract	3_CRP	5_LDLiPl2		2_HDL2
1_CK		2_blipo mg/	3_regCRP	5_HDL2		2_chol2
1_CK2		2_alipo	3_NPPB	5_chol2		2_TG2
**1_LPAov50**		2_preblipo	3_NPPB_GFR	5_TG2		2_CK
1_ApoB		2_a_lipo2	3_homocys	5_CK		2_CK2
1_regCRP		2_b_lipo2	3_vitD	5_CK2		2_LPA
2_NPPB_GFR		2_LDL	4_LDLbetafract	5_LPA		2_ApoA1
3_LDL		2_HDL	4_HDLalphafract	5_ApoA1		2_ApoB
3_TG		2_chol	4_VDLprebetafract	5_ApoB		2_CRP
3_TG2		2_TG	4_blipo	5_CRP		2_NPPB
3_CK		2_LDL2	4_alipo	5_regCRP		2_homocys
3_NPPB_GFR		2_HDL2	4_preblipo	5_NPPB		2_vitD
4_LDL2		2_chol2	4_alipo2	5_NPPB_GFR		
		2_TG2	4_blipo2	5_homocys		
		2_CK	4_LDL	5_vitD		
		2_CK2				
