# Supplementary material for: Cardiovascular risk assessment enhanced by automated machine learning in a multi-phase study
Source: Sci Rep. 2025 Oct 20;15:36474. doi: 10.1038/s41598-025-24189-z (PMC12537956; doi:10.1038/s41598-025-24189-z)
Supplement: Supplementary file 8 — Supplementary Material 8 [file 41598_2025_24189_MOESM8_ESM.pdf]

Phase 1

Target: LPA-L

Data Partitioning Methodology

Data partitions were selected by means of random sampling.

Model Features and Summary Statistics

Feature Name	Var Type	Unique	Missing	Mean	Std Dev	Median	Min	Max	Target Leakage
sex	Numeric	2	0	1.306	0.46	1.0	1.0	2.0	Low
cadyn	Numeric	2	0	0.78	0.41	1.0	0.0	1.0	Low
vdyn	Numeric	2	0	0.18	0.38	0.0	0.0	1.0	Low
exercise	Numeric	11	58	5.85	1.79	6.0	1.0	11.0	Low
urea	Numeric	102	2	39.39	15.46	36.0	7.0	209.0	Low
chol	Numeric	237	0	208.0053	43.56	205.0	79.0	453.0	Low
ferritin	Numeric	605	0	215.804	211.24	156.0	5.0	3316.0	Low
vitb12	Numeric	701	4	395.74	224.17	341.0	69.0	2000.0	Low
at3	Numeric	89	31	97.1008	13.54	97.0	36.0	147.0	Low
fii	Numeric	150	517	104.16	25.34	107.0	13.0	199.0	Low
vwfag	Numeric	303	11	170.21	72.85	158.0	21.0	552.0	Low
eapoa1	Numeric	147	0	129.4	24.73	127.0	58.0	241.0	Low
eapoa2	Categorical	451	0	N/A	N/A	N/A	N/A	N/A	Low
eapob	Numeric	145	0	104.26	24.63	103.0	32.0	245.0	Low
LPAov50	Numeric	2	0	0.2	0.4	0.0	0.0	1.0	N/A
vldlch	Numeric	148	0	36.608	25.41	31.0	0.0	350.0	Low
vldltg	Numeric	359	0	123.68	105.77	99.0	10.0	2379.0	Low
ldlch	Numeric	196	0	116.4	33.8	114.0	15.0	361.0	Low
miyn	Numeric	2	0	0.41	0.49	0.0	0.0	1.0	Low
Galectin3	Categorical	299	599	N/A	N/A	N/A	N/A	N/A	Low

Data Quality Handling Report

Feature Name	Var Type	Missing Count	Missing Percentage	Imputation Name	Imputation Description
Galectin3	Categorical	737	22	One-Hot Encoding	Missing indicator treated as feature
fii	Numeric	649	20	Missing Values Imputed	Missing indicator treated as feature, Imputed value: 107

exercise	Numeric	67	2	Missing Values Imputed	Missing indicator treated as feature, Imputed value: 6
at3	Numeric	36	1	Missing Values Imputed	Missing indicator treated as feature, Imputed value: 97
vwfag	Numeric	13	0	Missing Values Imputed	Missing indicator treated as feature, Imputed value: 158
vitb12	Numeric	4	0	Missing Values Imputed	Missing indicator treated as feature, Imputed value: 344
urea	Numeric	2	0	Missing Values Imputed	Missing indicator treated as feature, Imputed value: 36
eapoa2	Categorical	0	0	One-Hot Encoding	Missing values treated as infrequent
sex	Numeric	0	0	Missing Values Imputed	Imputed value: 1
cadyn	Numeric	0	0	Missing Values Imputed	Imputed value: 1
vdyn	Numeric	0	0	Missing Values Imputed	Imputed value: 0
chol	Numeric	0	0	Missing Values Imputed	Imputed value: 205
ferritin	Numeric	0	0	Missing Values Imputed	Imputed value: 156
eapoa1	Numeric	0	0	Missing Values Imputed	Imputed value: 127
eapob	Numeric	0	0	Missing Values Imputed	Imputed value: 103
vldlch	Numeric	0	0	Missing Values Imputed	Imputed value: 31
vldltg	Numeric	0	0	Missing Values Imputed	Imputed value: 99
ldlch	Numeric	0	0	Missing Values Imputed	Imputed value: 114
miyn	Numeric	0	0	Missing Values Imputed	Imputed value: 0

Target: CAD-L

Data Partitioning Methodology

The modeling partitions were randomly selected using a stratified sample to preserve the distribution of the target for each partition.

Model Features and Summary Statistics

Feature Name	Var Type	Unique	Missing	Mean	Std Dev	Median	Min	Max	Target Leakage
sex	Numeric	2	0	1.306	0.46	1.0	1.0	2.0	Low
strokeyn	Numeric	2	0	0.09	0.29	0.0	0.0	1.0	Low
carosten	Numeric	2	4	0.049	0.22	0.0	0.0	1.0	Low
pvdyn	Numeric	2	0	0.095	0.29	0.0	0.0	1.0	Low
dm1yn	Numeric	2	0	0.003	0.055	0.0	0.0	1.0	Low
dm2yn	Numeric	2	0	0.17	0.38	0.0	0.0	1.0	Low
canceryn	Numeric	2	4	0.075	0.26	0.0	0.0	1.0	Low
venthrom	Numeric	2	4	0.0604	0.24	0.0	0.0	1.0	Low
infectyn	Numeric	2	5	0.097	0.3	0.0	0.0	1.0	Low
cmpyn	Numeric	2	0	0.1006	0.3009	0.0	0.0	1.0	Low
vdyn	Numeric	2	0	0.18	0.38	0.0	0.0	1.0	Low
rhythyn	Numeric	2	29	0.15	0.36	0.0	0.0	1.0	Low
immunyn	Numeric	2	36	0.019	0.14	0.0	0.0	1.0	Low
anginayn	Numeric	2	95	0.54	0.5	1.0	0.0	1.0	Low
dyspneyn	Numeric	2	118	0.39	0.49	0.0	0.0	1.0	Low
exercise	Numeric	11	57	5.83	1.77	6.0	1.0	11.0	Low
crea	Numeric	33	2	0.98	0.39	0.9	0.5	9.6	Low
urea	Numeric	104	2	39.63	15.88	36.0	7.0	221.0	Low
uricacid	Numeric	110	1	5.13	1.709	4.9	0.9	17.8	Low
ldlch	N/A	0	3316	N/A	N/A	N/A	N/A	N/A	N/A
iron	Numeric	214	4	92.77	39.305	89.0	10.0	364.0	Low
ferritin	Numeric	620	0	220.36	215.52	160.0	5.0	3316.0	Low
ldh	Numeric	281	1	180.88	57.97	169.0	31.0	731.0	Low
hba1c	Numeric	85	3	6.32	1.23	6.0	3.1	16.5	Low
ace	Numeric	89	41	25.42	15.89	24.0	2.0	123.0	Low
vitb12	Numeric	682	3	397.67	228.5	345.0	74.0	2000.0	Low
folicac	Numeric	147	1	8.207	2.86	7.9	1.4	21.1	Low
tsh	Numeric	467	38	1.7	3.38	1.24	0.01	86.4	Low
vitd25	Numeric	404	15	17.44	9.84	15.5	2.5	170.1	Low
PTH	N/A	0	3316	N/A	N/A	N/A	N/A	N/A	N/A

hb	Numeric	97	0	13.83	1.5	13.9	7.9	18.5	Low
quick	Numeric	76	2	90.54	13.59	95.0	19.0	100.0	Low
aptt	Numeric	114	15	37.82	19.59	33.0	3.0	201.0	Low
fibrinog	Numeric	374	1	396.13	107.6	376.0	10.0	930.0	Low
at3	Numeric	88	28	97.13	13.43	97.0	36.0	146.0	Low
fii	Numeric	153	524	104.34	25.52	107.0	9.0	174.0	Low
vwfag	Numeric	304	8	169.76	72.707	158.0	21.0	552.0	Low
ddimer	Numeric	276	29	0.62	1.018	0.35	0.0	21.0	Low
tpaant	Numeric	314	3	13.63	7.094	12.0	1.3	96.0	Low
crp	Numeric	416	6	0.97	1.92	0.4	0.0	23.46	Low
haptoglo	Numeric	360	15	159.16	76.705	149.0	8.0	1137.0	Low
eapoa1	Numeric	150	0	129.38	25.055	127.0	52.0	249.0	Low
eapoa2	Numeric	454	0	41.57	9.57	41.1	2.1	123.0	Low
eapob	Numeric	150	0	104.84	24.88	103.0	32.0	245.0	Low
eapoe	Numeric	219	0	9.031	3.49	8.6	0.0	48.6	Low
elpa	Numeric	485	1	29.43	34.92	16.0	0.0	236.0	Low
vldlch	Numeric	150	0	37.26	26.69	31.0	0.0	350.0	Low
vldltg	Numeric	372	0	128.079	118.84	100.0	10.0	2379.0	Low
ldlch	Numeric	203	0	116.77	34.88	114.0	15.0	361.0	Low
ldltg	Numeric	83	1	31.7	11.92	30.0	2.0	117.0	Low
supercrp	Numeric	1039	5	8.84	17.61	3.4	0.17	199.0	Low
cystatc	Numeric	187	5	1.0001	0.404	0.91	0.35	6.8	Low
age	Numeric	2401	0	62.59	10.73	63.49	18.55	92.101	Low
bmi	Numeric	1679	0	27.58	4.098	27.17	16.34	48.34	Low
ishypert	Numeric	2	0	0.32	0.47	0.0	0.0	1.0	Low
smoclass	Numeric	3	0	0.84	0.73	1.0	0.0	2.0	Low
pbnpl1	Numeric	1241	41	905.62	2056.24	293.0	5.0	35000.0	Low
death2010	Numeric	2	0	0.306	0.46	0.0	0.0	1.0	Low
TnThs	Numeric	864	80	93.78	386.7	11.0	1.5	6640.0	Low
Galectin3	Numeric	303	576	15.74	6.903	14.6	3.0	97.5	Low

Data Quality Handling Report

**Feature Name**	**Var Type**	**Missing Count**	**Missing Percentage**	**Imputation Name**	**Imputation Description**
ldlch	Numeric	3316	100	Missing Values Imputed	Imputed value: 0
PTH	Numeric	3316	100	Missing Values Imputed	Imputed value: 0
Galectin3	Numeric	737	22	Missing Values Imputed	Missing indicator treated as feature, Imputed value: 14.5

fii	Numeric	650	20	Missing Values Imputed	Missing indicator treated as feature, Imputed value: 107
dyspneyn	Numeric	153	5	Missing Values Imputed	Missing indicator treated as feature, Imputed value: 0
anginayn	Numeric	125	4	Missing Values Imputed	Missing indicator treated as feature, Imputed value: 1
TnThs	Numeric	99	3	Missing Values Imputed	Missing indicator treated as feature, Imputed value: 11
exercise	Numeric	68	2	Missing Values Imputed	Missing indicator treated as feature, Imputed value: 6
pbnpl1	Numeric	51	2	Missing Values Imputed	Missing indicator treated as feature, Imputed value: 293
immunyn	Numeric	48	1	Missing Values Imputed	Missing indicator treated as feature, Imputed value: 0
ace	Numeric	48	1	Missing Values Imputed	Missing indicator treated as feature, Imputed value: 24
tsh	Numeric	45	1	Missing Values Imputed	Missing indicator treated as feature, Imputed value: 1.24
rhythyn	Numeric	42	1	Missing Values Imputed	Missing indicator treated as feature, Imputed value: 0
at3	Numeric	36	1	Missing Values Imputed	Missing indicator treated as feature, Imputed value: 97
ddimer	Numeric	35	1	Missing Values Imputed	Missing indicator treated as feature, Imputed value: 0.35
aptt	Numeric	21	1	Missing Values Imputed	Missing indicator treated as feature, Imputed value: 33
vitd25	Numeric	17	1	Missing Values Imputed	Missing indicator treated as feature, Imputed value: 15.6
haptoglo	Numeric	16	0	Missing Values Imputed	Missing indicator treated as feature, Imputed value: 150
vwfag	Numeric	13	0	Missing Values Imputed	Missing indicator treated as feature, Imputed value: 158
venthrom	Numeric	8	0	Missing Values Imputed	Missing indicator treated as feature, Imputed value: 0
infectyn	Numeric	6	0	Missing Values Imputed	Missing indicator treated as feature, Imputed value: 0
crp	Numeric	6	0	Missing Values Imputed	Missing indicator treated as feature, Imputed value: 0.41
supercrp	Numeric	6	0	Missing Values Imputed	Missing indicator treated as feature, Imputed value: 3.39

cystatc	Numeric	6	0	Missing Values Imputed	Imputed value: 0.92
carosten	Numeric	4	0	Missing Values Imputed	Missing indicator treated as feature, Imputed value: 0
canceryn	Numeric	4	0	Missing Values Imputed	Missing indicator treated as feature, Imputed value: 0
iron	Numeric	4	0	Missing Values Imputed	Missing indicator treated as feature, Imputed value: 89
hba1c	Numeric	4	0	Missing Values Imputed	Missing indicator treated as feature, Imputed value: 6
vitb12	Numeric	4	0	Missing Values Imputed	Missing indicator treated as feature, Imputed value: 344
quick	Numeric	4	0	Missing Values Imputed	Missing indicator treated as feature, Imputed value: 95
fibrinog	Numeric	4	0	Missing Values Imputed	Missing indicator treated as feature, Imputed value: 377
crea	Numeric	3	0	Missing Values Imputed	Missing indicator treated as feature, Imputed value: 0.9
tpaant	Numeric	3	0	Missing Values Imputed	Missing indicator treated as feature, Imputed value: 11.9
elpa	Numeric	3	0	Missing Values Imputed	Missing indicator treated as feature, Imputed value: 16
urea	Numeric	2	0	Missing Values Imputed	Missing indicator treated as feature, Imputed value: 36
ldltg	Numeric	2	0	Missing Values Imputed	Missing indicator treated as feature, Imputed value: 30
uricacid	Numeric	1	0	Missing Values Imputed	Missing indicator treated as feature, Imputed value: 4.9
ldh	Numeric	1	0	Missing Values Imputed	Missing indicator treated as feature, Imputed value: 168
folicac	Numeric	1	0	Missing Values Imputed	Missing indicator treated as feature, Imputed value: 7.9
eapoa1	Numeric	1	0	Missing Values Imputed	Missing indicator treated as feature, Imputed value: 127
eapoa2	Numeric	1	0	Missing Values Imputed	Imputed value: 41.2
eapob	Numeric	1	0	Missing Values Imputed	Imputed value: 103
eapoe	Numeric	1	0	Missing Values Imputed	Imputed value: 8.6
vldlch	Numeric	1	0	Missing Values Imputed	Imputed value: 31
vldltg	Numeric	1	0	Missing Values Imputed	Imputed value: 99
ldlch	Numeric	1	0	Missing Values Imputed	Imputed value: 114
sex	Numeric	0	0	Missing Values Imputed	Imputed value: 1

strokeyn	Numeric	0	0	Missing Values Imputed	Imputed value: 0
pvdyn	Numeric	0	0	Missing Values Imputed	Imputed value: 0
dm1yn	Numeric	0	0	Missing Values Imputed	Imputed value: 0
dm2yn	Numeric	0	0	Missing Values Imputed	Imputed value: 0
cmpyn	Numeric	0	0	Missing Values Imputed	Imputed value: 0
vdyn	Numeric	0	0	Missing Values Imputed	Imputed value: 0
ferritin	Numeric	0	0	Missing Values Imputed	Imputed value: 156
hb	Numeric	0	0	Missing Values Imputed	Imputed value: 13.9
age	Numeric	0	0	Missing Values Imputed	Imputed value: 63.5418
bmi	Numeric	0	0	Missing Values Imputed	Imputed value: 27.0602
ishypert	Numeric	0	0	Missing Values Imputed	Imputed value: 0
smoclass	Numeric	0	0	Missing Values Imputed	Imputed value: 1
death2010	Numeric	0	0	Missing Values Imputed	Imputed value: 0

Target: Early CAD-L

Data Partitioning Methodology

Data partitions were selected by means of random sampling.

Model Features and Summary Statistics

Feature Name	Var Type	Unique	Missing	Mean	Std Dev	Median	Min	Max	Target Leakage
sex	Numeric	2	0	1.26	0.44	1.0	1.0	2.0	Low
cadyn	Numeric	1	0	1.0	0.0	1.0	1.0	1.0	Low
strokeyn	Numeric	2	0	0.099	0.3	0.0	0.0	1.0	Low
carosten	Numeric	2	3	0.058	0.23	0.0	0.0	1.0	Low
pvdyn	Numeric	2	0	0.11	0.32	0.0	0.0	1.0	Low
dm1yn	Numeric	2	0	0.0039	0.062	0.0	0.0	1.0	Low
dm2yn	Numeric	2	0	0.2	0.4	0.0	0.0	1.0	Low
canceryn	Numeric	2	3	0.081	0.27	0.0	0.0	1.0	Low
venthrom	Numeric	2	2	0.0606	0.24	0.0	0.0	1.0	Low
infectyn	Numeric	2	5	0.098	0.3	0.0	0.0	1.0	Low
cmpyn	Numeric	2	0	0.086	0.28	0.0	0.0	1.0	Low
vdyn	Numeric	2	0	0.16	0.37	0.0	0.0	1.0	Low
rhythyn	Numeric	2	26	0.13	0.34	0.0	0.0	1.0	Low
immunyn	Numeric	2	31	0.019	0.14	0.0	0.0	1.0	Low
anginayn	Numeric	2	71	0.56	0.5	1.0	0.0	1.0	Low
dyspneyn	Numeric	2	93	0.38	0.49	0.0	0.0	1.0	Low
exercise	Numeric	11	44	5.76	1.75	6.0	1.0	11.0	Low
crea	Numeric	32	2	1.0	0.42	0.9	0.5	9.6	Low
urea	Numeric	101	1	40.49	16.27	37.0	7.0	221.0	Low
uricacid	Numeric	106	1	5.21	1.69	5.0	0.9	17.8	Low
ldlch	N/A	0	3316	N/A	N/A	N/A	N/A	N/A	N/A
iron	Numeric	201	4	90.49	38.803	86.0	12.0	364.0	Low
ferritin	Numeric	575	0	221.087	219.081	160.0	5.0	3316.0	Low
ldh	Numeric	270	1	182.68	60.87	169.0	31.0	731.0	Low
hba1c	Numeric	83	3	6.41	1.3	6.1	3.1	16.5	Low
ace	Numeric	85	33	24.26	15.68	23.0	2.0	111.0	Low
vitb12	Numeric	631	3	397.92	232.2007	344.0	74.0	2000.0	Low
folicac	Numeric	140	1	8.17	2.86	7.9	1.8	21.1	Low
tsh	Numeric	435	27	1.703	3.54	1.24	0.01	86.4	Low
vitd25	Numeric	381	12	17.22	9.47	15.4	2.5	75.6	Low

PTH	N/A	0	3316	N/A	N/A	N/A	N/A	N/A	N/A
hb	Numeric	95	0	13.81	1.51	13.9	8.4	18.5	Low
quick	Numeric	72	2	90.77	12.99	95.0	23.0	100.0	Low
aptt	Numeric	100	13	37.65	18.28	33.0	3.0	201.0	Low
fibrinog	Numeric	357	1	405.85	109.66	387.5	141.0	930.0	Low
at3	Numeric	84	22	96.52	13.45	96.0	36.0	146.0	Low
fii	Numeric	142	410	104.7	24.59	106.0	9.0	174.0	Low
vwfag	Numeric	287	9	174.34	73.93	162.0	21.0	542.0	Low
ddimer	Numeric	265	26	0.66	1.089	0.37	0.0	21.0	Low
tpaant	Numeric	294	2	13.91	6.9	12.2	2.5	72.6	Low
crp	Numeric	390	5	1.055	2.024	0.45	0.01	23.46	Low
haptoglo	Numeric	342	9	164.17	75.32	155.0	8.0	594.0	Low
eapoa1	Numeric	138	1	127.19	24.25	124.0	52.0	241.0	Low
eapoa2	Numeric	415	1	40.98	9.32	40.4	2.1	123.0	Low
eapob	Numeric	146	1	105.12	25.22	103.0	42.0	245.0	Low
eapoe	Numeric	200	1	8.83	3.501	8.45	0.0	48.6	Low
elpa	Numeric	440	2	30.72	36.51	17.0	0.0	236.0	Low
vldlch	Numeric	141	1	37.66	26.068	31.0	0.0	254.0	Low
vldltg	Numeric	348	1	130.34	111.11	103.0	10.0	1385.0	Low
ldlch	Numeric	193	1	115.39	35.0504	112.0	20.0	361.0	Low
ldltg	Numeric	80	2	32.17	11.85	30.0	2.0	105.0	Low
supercrp	Numeric	961	4	9.61	18.49	3.76	0.17	199.0	Low
cystatc	Numeric	180	4	1.0204	0.43	0.93	0.35	6.8	Low
age	Numeric	1899	0	63.74	9.96	64.48	29.26	92.101	Low
bmi	Numeric	1367	0	27.58	4.071	27.18	16.75	57.099	Low
ishypert	Numeric	2	0	0.34	0.47	0.0	0.0	1.0	Low
smoclass	Numeric	3	0	0.89	0.709	1.0	0.0	2.0	Low
miyn	Numeric	2	0	0.53	0.5	1.0	0.0	1.0	Low
pbnpl1	Numeric	1111	35	961.13	2144.74	337.0	5.0	35000.0	Low
death2010	Numeric	2	0	0.35	0.48	0.0	0.0	1.0	Low
TnThs	Numeric	744	58	113.48	436.41	12.76	1.5	6640.0	Low
Galectin3	Numeric	282	470	15.93	7.0805	14.7	3.0	97.5	Low

Data Quality Handling Report

**Feature Name**	**Var Type**	**Missing Count**	**Missing Percentage**	**Imputation Name**	**Imputation Description**
ldlch	Numeric	2582	100	Missing Values Imputed	Imputed value: 0
PTH	Numeric	2582	100	Missing Values Imputed	Imputed value: 0

Galectin3	Numeric	597	23	Missing Values Imputed	Missing indicator treated as feature, Imputed value: 14.7
fii	Numeric	515	20	Missing Values Imputed	Missing indicator treated as feature, Imputed value: 106
dyspneyn	Numeric	120	5	Missing Values Imputed	Missing indicator treated as feature, Imputed value: 0
anginayn	Numeric	92	4	Missing Values Imputed	Missing indicator treated as feature, Imputed value: 1
TnThs	Numeric	73	3	Missing Values Imputed	Missing indicator treated as feature, Imputed value: 12.31
exercise	Numeric	47	2	Missing Values Imputed	Missing indicator treated as feature, Imputed value: 6
pbnpl1	Numeric	45	2	Missing Values Imputed	Missing indicator treated as feature, Imputed value: 333
ace	Numeric	40	2	Missing Values Imputed	Missing indicator treated as feature, Imputed value: 22
immunyn	Numeric	39	2	Missing Values Imputed	Missing indicator treated as feature, Imputed value: 0
rhythyn	Numeric	35	1	Missing Values Imputed	Missing indicator treated as feature, Imputed value: 0
tsh	Numeric	31	1	Missing Values Imputed	Missing indicator treated as feature, Imputed value: 1.23
ddimer	Numeric	31	1	Missing Values Imputed	Missing indicator treated as feature, Imputed value: 0.37
at3	Numeric	28	1	Missing Values Imputed	Missing indicator treated as feature, Imputed value: 96
aptt	Numeric	17	1	Missing Values Imputed	Missing indicator treated as feature, Imputed value: 33
vitd25	Numeric	14	1	Missing Values Imputed	Missing indicator treated as feature, Imputed value: 15.5
vwfag	Numeric	12	0	Missing Values Imputed	Missing indicator treated as feature, Imputed value: 162
haptoglo	Numeric	9	0	Missing Values Imputed	Missing indicator treated as feature, Imputed value: 155
venthrom	Numeric	5	0	Missing Values Imputed	Missing indicator treated as feature, Imputed value: 0
infectyn	Numeric	5	0	Missing Values Imputed	Missing indicator treated as feature, Imputed value: 0
crp	Numeric	5	0	Missing Values Imputed	Missing indicator treated as feature, Imputed value: 0.45

supercrp	Numeric	5	0	Missing Values Imputed	Missing indicator treated as feature, Imputed value: 3.82
cystatc	Numeric	5	0	Missing Values Imputed	Imputed value: 0.93
iron	Numeric	4	0	Missing Values Imputed	Missing indicator treated as feature, Imputed value: 86
hba1c	Numeric	4	0	Missing Values Imputed	Missing indicator treated as feature, Imputed value: 6.1
vitb12	Numeric	4	0	Missing Values Imputed	Missing indicator treated as feature, Imputed value: 343
carosten	Numeric	3	0	Missing Values Imputed	Missing indicator treated as feature, Imputed value: 0
canceryn	Numeric	3	0	Missing Values Imputed	Missing indicator treated as feature, Imputed value: 0
crea	Numeric	3	0	Missing Values Imputed	Missing indicator treated as feature, Imputed value: 0.9
quick	Numeric	3	0	Missing Values Imputed	Missing indicator treated as feature, Imputed value: 95
elpa	Numeric	3	0	Missing Values Imputed	Missing indicator treated as feature, Imputed value: 17
fibrinog	Numeric	2	0	Missing Values Imputed	Missing indicator treated as feature, Imputed value: 388
tpaant	Numeric	2	0	Missing Values Imputed	Missing indicator treated as feature, Imputed value: 12.2
ldltg	Numeric	2	0	Missing Values Imputed	Missing indicator treated as feature, Imputed value: 30
urea	Numeric	1	0	Missing Values Imputed	Missing indicator treated as feature, Imputed value: 37
uricacid	Numeric	1	0	Missing Values Imputed	Missing indicator treated as feature, Imputed value: 5
ldh	Numeric	1	0	Missing Values Imputed	Missing indicator treated as feature, Imputed value: 169
folicac	Numeric	1	0	Missing Values Imputed	Missing indicator treated as feature, Imputed value: 7.8
eapoa1	Numeric	1	0	Missing Values Imputed	Missing indicator treated as feature, Imputed value: 124
eapoa2	Numeric	1	0	Missing Values Imputed	Imputed value: 40.4
eapob	Numeric	1	0	Missing Values Imputed	Imputed value: 103
eapoe	Numeric	1	0	Missing Values Imputed	Imputed value: 8.4
vldlch	Numeric	1	0	Missing Values Imputed	Imputed value: 31
vldltg	Numeric	1	0	Missing Values Imputed	Imputed value: 102

ldlch	Numeric	1	0	Missing Values Imputed	Imputed value: 112
sex	Numeric	0	0	Missing Values Imputed	Imputed value: 1
cadyn	Numeric	0	0	Missing Values Imputed	Imputed value: 0
strokeyn	Numeric	0	0	Missing Values Imputed	Imputed value: 0
pvdyn	Numeric	0	0	Missing Values Imputed	Imputed value: 0
dm1yn	Numeric	0	0	Missing Values Imputed	Imputed value: 0
dm2yn	Numeric	0	0	Missing Values Imputed	Imputed value: 0
cmpyn	Numeric	0	0	Missing Values Imputed	Imputed value: 0
vdyn	Numeric	0	0	Missing Values Imputed	Imputed value: 0
ferritin	Numeric	0	0	Missing Values Imputed	Imputed value: 160
hb	Numeric	0	0	Missing Values Imputed	Imputed value: 13.9
age	Numeric	0	0	Missing Values Imputed	Imputed value: 64.4891
bmi	Numeric	0	0	Missing Values Imputed	Imputed value: 27.1527
ishypert	Numeric	0	0	Missing Values Imputed	Imputed value: 0
smoclass	Numeric	0	0	Missing Values Imputed	Imputed value: 1
miyn	Numeric	0	0	Missing Values Imputed	Imputed value: 1
death2010	Numeric	0	0	Missing Values Imputed	Imputed value: 0

Target: MI-L

Data Partitioning Methodology

Data partitions were selected by means of random sampling.

Model Features and Summary Statistics

Feature Name	Var Type	Unique	Missing	Mean	Std Dev	Median	Min	Max	Target Leakage
sex	Numeric	2	0	1.301	0.46	1.0	1.0	2.0	Low
crea	Numeric	33	3	0.98	0.41	0.9	0.5	9.6	Low
urea	Numeric	102	2	39.56	15.88	36.0	7.0	221.0	Low
uricacid	Numeric	109	0	5.14	1.72	4.9	0.9	17.8	Low
ldlch	N/A	0	3316	N/A	N/A	N/A	N/A	N/A	N/A
iron	Numeric	210	3	92.57	39.07	88.0	10.0	364.0	Low
ferritin	Numeric	611	0	216.35	210.42	156.0	5.0	3316.0	Low
hba1c	Numeric	85	3	6.32	1.25	6.0	3.9	18.6	Low
ace	Numeric	92	37	25.24	15.92	24.0	1.0	118.0	Low
vitb12	Numeric	696	3	394.38	221.43	343.0	69.0	2000.0	Low
folicac	Numeric	148	0	8.17	2.83	7.9	1.4	21.1	Low
tsh	Numeric	467	29	1.75	4.4	1.24	0.01	134.9	Low
vitd25	Numeric	399	14	17.24	9.24	15.5	1.9	75.6	Low
PTH	N/A	0	3316	N/A	N/A	N/A	N/A	N/A	N/A
hb	Numeric	94	0	13.83	1.49	13.9	7.9	18.5	Low
quick	Numeric	75	4	90.4	13.83	95.0	19.0	100.0	Low
fibrinog	Numeric	373	4	397.14	108.409	378.0	10.0	930.0	Low
at3	Numeric	88	32	97.085	13.48	97.0	40.0	147.0	Low
fii	Numeric	154	526	104.055	26.0024	107.0	9.0	199.0	Low
vwfag	Numeric	305	12	170.25	72.69	158.0	21.0	552.0	Low
ddimer	Numeric	279	31	0.63	1.075	0.36	0.0	21.0	Low
tpaant	Numeric	312	3	13.53	7.059	11.9	1.3	96.0	Low
crp	Numeric	425	5	1.0	2.036	0.4	0.01	28.24	Low
haptoglo	Numeric	361	11	158.62	76.6	149.0	8.0	1137.0	Low
eapoa1	Numeric	149	1	129.53	24.97	127.0	52.0	241.0	Low
eapoa2	Numeric	454	1	41.58	9.42	41.2	2.1	85.6	Low
eapob	Numeric	148	1	104.014	24.93	102.0	32.0	245.0	Low
eapoe	Numeric	220	1	8.98	3.34	8.6	0.0	39.0	Low
elpa	Numeric	478	2	28.67	34.093	16.0	0.0	236.0	Low
vldlch	Numeric	146	1	36.52	25.35	31.0	0.0	350.0	Low
vldltg	Numeric	360	1	123.98	108.99	99.0	10.0	2379.0	Low

ldlch	Numeric	198	1	116.204	34.37	113.0	15.0	361.0	Low
ldltg	Numeric	80	1	31.47	11.64	30.0	2.0	133.0	Low
supercrp	Numeric	1049	4	9.17	18.84	3.34	0.17	269.0	Low
cystatc	Numeric	186	4	1.0019	0.42	0.92	0.35	7.44	Low
age	Numeric	2417	0	62.63	10.62	63.58	17.25	92.101	Low
bmi	Numeric	1660	0	27.5	4.072	27.055	16.35	48.34	Low
smoclass	Numeric	3	0	0.84	0.73	1.0	0.0	2.0	Low
pbnpl1	Numeric	1242	33	930.77	2179.35	294.0	5.0	35000.0	Low
TnThs	Numeric	849	74	101.93	426.83	11.0	1.5	6640.0	Low
Galectin3	Numeric	300	595	15.71	7.24	14.4	1.7	100.1	Low

Data Quality Handling Report

**Feature Name**	**Var Type**	**Missing Count**	**Missing Percentage**	**Imputation Name**	**Imputation Description**
ldlch	Numeric	2122	100	Missing Values Imputed	Imputed value: 0
PTH	Numeric	2122	100	Missing Values Imputed	Imputed value: 0
Galectin3	Numeric	487	23	Missing Values Imputed	Missing indicator treated as feature, Imputed value: 14.4
fii	Numeric	419	20	Missing Values Imputed	Missing indicator treated as feature, Imputed value: 107
TnThs	Numeric	59	3	Missing Values Imputed	Missing indicator treated as feature, Imputed value: 11
ace	Numeric	30	1	Missing Values Imputed	Missing indicator treated as feature, Imputed value: 24
at3	Numeric	28	1	Missing Values Imputed	Missing indicator treated as feature, Imputed value: 97
pbnpl1	Numeric	26	1	Missing Values Imputed	Missing indicator treated as feature, Imputed value: 299
tsh	Numeric	25	1	Missing Values Imputed	Missing indicator treated as feature, Imputed value: 1.23
ddimer	Numeric	22	1	Missing Values Imputed	Missing indicator treated as feature, Imputed value: 0.36
vitd25	Numeric	10	0	Missing Values Imputed	Missing indicator treated as feature, Imputed value: 15.6
vwfag	Numeric	10	0	Missing Values Imputed	Missing indicator treated as feature, Imputed value: 158
haptoglo	Numeric	9	0	Missing Values Imputed	Missing indicator treated as feature, Imputed value: 149
crp	Numeric	4	0	Missing Values Imputed	Missing indicator treated as feature, Imputed value: 0.41

supercrp	Numeric	4	0	Missing Values Imputed	Missing indicator treated as feature, Imputed value: 3.42
cystatc	Numeric	4	0	Missing Values Imputed	Imputed value: 0.92
crea	Numeric	3	0	Missing Values Imputed	Missing indicator treated as feature, Imputed value: 0.9
iron	Numeric	3	0	Missing Values Imputed	Missing indicator treated as feature, Imputed value: 88
quick	Numeric	3	0	Missing Values Imputed	Missing indicator treated as feature, Imputed value: 95
tpaant	Numeric	3	0	Missing Values Imputed	Missing indicator treated as feature, Imputed value: 12
hba1c	Numeric	2	0	Missing Values Imputed	Missing indicator treated as feature, Imputed value: 6
vitb12	Numeric	2	0	Missing Values Imputed	Missing indicator treated as feature, Imputed value: 341
fibrinog	Numeric	2	0	Missing Values Imputed	Missing indicator treated as feature, Imputed value: 380
elpa	Numeric	2	0	Missing Values Imputed	Missing indicator treated as feature, Imputed value: 16
urea	Numeric	1	0	Missing Values Imputed	Missing indicator treated as feature, Imputed value: 36
eapoa1	Numeric	1	0	Missing Values Imputed	Missing indicator treated as feature, Imputed value: 127
eapoa2	Numeric	1	0	Missing Values Imputed	Imputed value: 41.2
eapob	Numeric	1	0	Missing Values Imputed	Imputed value: 102
eapoe	Numeric	1	0	Missing Values Imputed	Imputed value: 8.6
vldlch	Numeric	1	0	Missing Values Imputed	Imputed value: 31
vldltg	Numeric	1	0	Missing Values Imputed	Imputed value: 99
ldlch	Numeric	1	0	Missing Values Imputed	Imputed value: 114
ldltg	Numeric	1	0	Missing Values Imputed	Imputed value: 30
sex	Numeric	0	0	Missing Values Imputed	Imputed value: 1
uricacid	Numeric	0	0	Missing Values Imputed	Imputed value: 4.9
ferritin	Numeric	0	0	Missing Values Imputed	Imputed value: 154
folicac	Numeric	0	0	Missing Values Imputed	Imputed value: 7.9
hb	Numeric	0	0	Missing Values Imputed	Imputed value: 13.9
age	Numeric	0	0	Missing Values Imputed	Imputed value: 63.4925
bmi	Numeric	0	0	Missing Values Imputed	Imputed value: 27.1178
smoclass	Numeric	0	0	Missing Values Imputed	Imputed value: 1

Target: Stroke-L

Data Partitioning Methodology

Data partitions were selected by means of random sampling.

Model Features and Summary Statistics

Feature Name	Var Type	Unique	Missing	Mean	Std Dev	Median	Min	Max	Target Leakage
sex	Numeric	2	0	1.301	0.46	1.0	1.0	2.0	Low
cadyn	Numeric	2	0	0.78	0.41	1.0	0.0	1.0	Low
strokeyn	Numeric	2	0	0.092	0.29	0.0	0.0	1.0	N/A
carosten	Numeric	2	3	0.0502	0.22	0.0	0.0	1.0	Low
pvdyn	Numeric	2	0	0.093	0.29	0.0	0.0	1.0	Low
dm1yn	Numeric	2	0	0.0026	0.051	0.0	0.0	1.0	Low
dm2yn	Numeric	2	0	0.18	0.38	0.0	0.0	1.0	Low
canceryn	Numeric	2	4	0.069	0.25	0.0	0.0	1.0	Low
venthrom	Numeric	2	6	0.063	0.24	0.0	0.0	1.0	Low
infectyn	Numeric	2	4	0.096	0.29	0.0	0.0	1.0	Low
cmpyn	Numeric	2	0	0.097	0.3	0.0	0.0	1.0	Low
vdyn	Numeric	2	0	0.18	0.38	0.0	0.0	1.0	Low
rhythyn	Numeric	2	32	0.15	0.35	0.0	0.0	1.0	Low
immunyn	Numeric	2	38	0.0207	0.14	0.0	0.0	1.0	Low
anginayn	Numeric	3	105	0.54	0.5	1.0	0.0	2.0	Low
dyspneyn	Numeric	3	121	0.39	0.49	0.0	0.0	2.0	Low
exercise	Numeric	11	58	5.81	1.77	6.0	1.0	11.0	Low
crea	Numeric	33	3	0.98	0.41	0.9	0.5	9.6	Low
urea	Numeric	102	2	39.56	15.88	36.0	7.0	221.0	Low
uricacid	Numeric	109	0	5.14	1.72	4.9	0.9	17.8	Low
ldlch	N/A	0	3316	N/A	N/A	N/A	N/A	N/A	N/A
iron	Numeric	210	3	92.57	39.07	88.0	10.0	364.0	Low
ferritin	Numeric	611	0	216.35	210.42	156.0	5.0	3316.0	Low
ldh	Numeric	290	1	182.069	61.4003	169.0	31.0	731.0	Low
hba1c	Numeric	85	3	6.32	1.25	6.0	3.9	18.6	Low
ace	Numeric	92	37	25.24	15.92	24.0	1.0	118.0	Low
vitb12	Numeric	696	3	394.38	221.43	343.0	69.0	2000.0	Low
folicac	Numeric	148	0	8.17	2.83	7.9	1.4	21.1	Low
tsh	Numeric	467	29	1.75	4.4	1.24	0.01	134.9	Low
vitd25	Numeric	399	14	17.24	9.24	15.5	1.9	75.6	Low
PTH	N/A	0	3316	N/A	N/A	N/A	N/A	N/A	N/A

hb	Numeric	94	0	13.83	1.49	13.9	7.9	18.5	Low
quick	Numeric	75	4	90.4	13.83	95.0	19.0	100.0	Low
aptt	Numeric	115	18	37.92	19.7	33.0	24.0	201.0	Low
fibrinog	Numeric	373	4	397.14	108.409	378.0	10.0	930.0	Low
at3	Numeric	88	32	97.085	13.48	97.0	40.0	147.0	Low
fii	Numeric	154	526	104.055	26.0024	107.0	9.0	199.0	Low
vwfag	Numeric	305	12	170.25	72.69	158.0	21.0	552.0	Low
ddimer	Numeric	279	31	0.63	1.075	0.36	0.0	21.0	Low
tpaant	Numeric	312	3	13.53	7.059	11.9	1.3	96.0	Low
crp	Numeric	425	5	1.0	2.036	0.4	0.01	28.24	Low
haptoglo	Numeric	361	11	158.62	76.6	149.0	8.0	1137.0	Low
eapoa1	Numeric	149	1	129.53	24.97	127.0	52.0	241.0	Low
eapoa2	Numeric	454	1	41.58	9.42	41.2	2.1	85.6	Low
eapob	Numeric	148	1	104.014	24.93	102.0	32.0	245.0	Low
eapoe	Numeric	220	1	8.98	3.34	8.6	0.0	39.0	Low
elpa	Numeric	478	2	28.67	34.093	16.0	0.0	236.0	Low
vldlch	Numeric	146	1	36.52	25.35	31.0	0.0	350.0	Low
vldltg	Numeric	360	1	123.98	108.99	99.0	10.0	2379.0	Low
ldlch	Numeric	198	1	116.204	34.37	113.0	15.0	361.0	Low
ldltg	Numeric	80	1	31.47	11.64	30.0	2.0	133.0	Low
supercrp	Numeric	1049	4	9.17	18.84	3.34	0.17	269.0	Low
cystatc	Numeric	186	4	1.0019	0.42	0.92	0.35	7.44	Low
age	Numeric	2417	0	62.63	10.62	63.58	17.25	92.101	Low
bmi	Numeric	1660	0	27.5	4.072	27.055	16.35	48.34	Low
ishypert	Numeric	2	0	0.31	0.46	0.0	0.0	1.0	Low
smoclass	Numeric	3	0	0.84	0.73	1.0	0.0	2.0	Low
miyn	Numeric	2	0	0.42	0.49	0.0	0.0	1.0	Low
pbnpl1	Numeric	1242	33	930.77	2179.35	294.0	5.0	35000.0	Low
death2010	Numeric	2	0	0.3	0.46	0.0	0.0	1.0	Low
TnThs	Numeric	849	74	101.93	426.83	11.0	1.5	6640.0	Low
Galectin3	Numeric	300	595	15.71	7.24	14.4	1.7	100.1	Low

Data Quality Handling Report

**Feature Name**	**Var Type**	**Missing Count**	**Missing Percentage**	**Imputation Name**	**Imputation Description**
ldlch	Numeric	3316	100	Missing Values Imputed	Imputed value: 0
PTH	Numeric	3316	100	Missing Values Imputed	Imputed value: 0
Galectin3	Numeric	737	22	Missing Values Imputed	Missing indicator treated as feature, Imputed value: 14.5

fii	Numeric	650	20	Missing Values Imputed	Missing indicator treated as feature, Imputed value: 107
dyspneyn	Numeric	153	5	Missing Values Imputed	Missing indicator treated as feature, Imputed value: 0
anginayn	Numeric	125	4	Missing Values Imputed	Missing indicator treated as feature, Imputed value: 1
TnThs	Numeric	99	3	Missing Values Imputed	Missing indicator treated as feature, Imputed value: 11
exercise	Numeric	68	2	Missing Values Imputed	Missing indicator treated as feature, Imputed value: 6
pbnpl1	Numeric	51	2	Missing Values Imputed	Missing indicator treated as feature, Imputed value: 293
immunyn	Numeric	48	1	Missing Values Imputed	Missing indicator treated as feature, Imputed value: 0
ace	Numeric	48	1	Missing Values Imputed	Missing indicator treated as feature, Imputed value: 24
tsh	Numeric	45	1	Missing Values Imputed	Missing indicator treated as feature, Imputed value: 1.24
rhythyn	Numeric	42	1	Missing Values Imputed	Missing indicator treated as feature, Imputed value: 0
at3	Numeric	36	1	Missing Values Imputed	Missing indicator treated as feature, Imputed value: 97
ddimer	Numeric	35	1	Missing Values Imputed	Missing indicator treated as feature, Imputed value: 0.35
aptt	Numeric	21	1	Missing Values Imputed	Missing indicator treated as feature, Imputed value: 33
vitd25	Numeric	17	1	Missing Values Imputed	Missing indicator treated as feature, Imputed value: 15.6
haptoglo	Numeric	16	0	Missing Values Imputed	Missing indicator treated as feature, Imputed value: 150
vwfag	Numeric	13	0	Missing Values Imputed	Missing indicator treated as feature, Imputed value: 158
venthrom	Numeric	8	0	Missing Values Imputed	Missing indicator treated as feature, Imputed value: 0
infectyn	Numeric	6	0	Missing Values Imputed	Missing indicator treated as feature, Imputed value: 0
crp	Numeric	6	0	Missing Values Imputed	Missing indicator treated as feature, Imputed value: 0.41
supercrp	Numeric	6	0	Missing Values Imputed	Missing indicator treated as feature, Imputed value: 3.39

cystatc	Numeric	6	0	Missing Values Imputed	Imputed value: 0.92
carosten	Numeric	4	0	Missing Values Imputed	Missing indicator treated as feature, Imputed value: 0
canceryn	Numeric	4	0	Missing Values Imputed	Missing indicator treated as feature, Imputed value: 0
iron	Numeric	4	0	Missing Values Imputed	Missing indicator treated as feature, Imputed value: 89
hba1c	Numeric	4	0	Missing Values Imputed	Missing indicator treated as feature, Imputed value: 6
vitb12	Numeric	4	0	Missing Values Imputed	Missing indicator treated as feature, Imputed value: 344
quick	Numeric	4	0	Missing Values Imputed	Missing indicator treated as feature, Imputed value: 95
fibrinog	Numeric	4	0	Missing Values Imputed	Missing indicator treated as feature, Imputed value: 377
crea	Numeric	3	0	Missing Values Imputed	Missing indicator treated as feature, Imputed value: 0.9
tpaant	Numeric	3	0	Missing Values Imputed	Missing indicator treated as feature, Imputed value: 11.9
elpa	Numeric	3	0	Missing Values Imputed	Missing indicator treated as feature, Imputed value: 16
urea	Numeric	2	0	Missing Values Imputed	Missing indicator treated as feature, Imputed value: 36
ldltg	Numeric	2	0	Missing Values Imputed	Missing indicator treated as feature, Imputed value: 30
uricacid	Numeric	1	0	Missing Values Imputed	Missing indicator treated as feature, Imputed value: 4.9
ldh	Numeric	1	0	Missing Values Imputed	Missing indicator treated as feature, Imputed value: 168
folicac	Numeric	1	0	Missing Values Imputed	Missing indicator treated as feature, Imputed value: 7.9
eapoa1	Numeric	1	0	Missing Values Imputed	Missing indicator treated as feature, Imputed value: 127
eapoa2	Numeric	1	0	Missing Values Imputed	Imputed value: 41.2
eapob	Numeric	1	0	Missing Values Imputed	Imputed value: 103
eapoe	Numeric	1	0	Missing Values Imputed	Imputed value: 8.6
vldlch	Numeric	1	0	Missing Values Imputed	Imputed value: 31
vldltg	Numeric	1	0	Missing Values Imputed	Imputed value: 99
ldlch	Numeric	1	0	Missing Values Imputed	Imputed value: 114
sex	Numeric	0	0	Missing Values Imputed	Imputed value: 1

cadyn	Numeric	0	0	Missing Values Imputed	Imputed value: 1
pvdyn	Numeric	0	0	Missing Values Imputed	Imputed value: 0
dm1yn	Numeric	0	0	Missing Values Imputed	Imputed value: 0
dm2yn	Numeric	0	0	Missing Values Imputed	Imputed value: 0
cmpyn	Numeric	0	0	Missing Values Imputed	Imputed value: 0
vdyn	Numeric	0	0	Missing Values Imputed	Imputed value: 0
ferritin	Numeric	0	0	Missing Values Imputed	Imputed value: 156
hb	Numeric	0	0	Missing Values Imputed	Imputed value: 13.9
age	Numeric	0	0	Missing Values Imputed	Imputed value: 63.5418
bmi	Numeric	0	0	Missing Values Imputed	Imputed value: 27.0602
ishypert	Numeric	0	0	Missing Values Imputed	Imputed value: 0
smoclass	Numeric	0	0	Missing Values Imputed	Imputed value: 1
miyn	Numeric	0	0	Missing Values Imputed	Imputed value: 0
death2010	Numeric	0	0	Missing Values Imputed	Imputed value: 0

Target: PAD-L

Data Partitioning Methodology

Data partitions were selected by means of random sampling.

Model Features and Summary Statistics

Feature Name	Var Type	Unique	Missing	Mean	Std Dev	Median	Min	Max	Target Leakage
sex	Numeric	2	0	1.301	0.46	1.0	1.0	2.0	Low
cadyn	Numeric	2	0	0.78	0.41	1.0	0.0	1.0	Low
strokeyn	Numeric	2	0	0.092	0.29	0.0	0.0	1.0	Low
carosten	Numeric	2	3	0.0502	0.22	0.0	0.0	1.0	Low
pvdyn	Numeric	2	0	0.093	0.29	0.0	0.0	1.0	N/A
dm1yn	Numeric	2	0	0.0026	0.051	0.0	0.0	1.0	Low
dm2yn	Numeric	2	0	0.18	0.38	0.0	0.0	1.0	Low
canceryn	Numeric	2	4	0.069	0.25	0.0	0.0	1.0	Low
venthrom	Numeric	2	6	0.063	0.24	0.0	0.0	1.0	Low
infectyn	Numeric	2	4	0.096	0.29	0.0	0.0	1.0	Low
cmpyn	Numeric	2	0	0.097	0.3	0.0	0.0	1.0	Low
vdyn	Numeric	2	0	0.18	0.38	0.0	0.0	1.0	Low
rhythyn	Numeric	2	32	0.15	0.35	0.0	0.0	1.0	Low
immunyn	Numeric	2	38	0.0207	0.14	0.0	0.0	1.0	Low
anginayn	Numeric	3	105	0.54	0.5	1.0	0.0	2.0	Low
dyspneyn	Numeric	3	121	0.39	0.49	0.0	0.0	2.0	Low
exercise	Numeric	11	58	5.81	1.77	6.0	1.0	11.0	Low
crea	Numeric	33	3	0.98	0.41	0.9	0.5	9.6	Low
urea	Numeric	102	2	39.56	15.88	36.0	7.0	221.0	Low
uricacid	Numeric	109	0	5.14	1.72	4.9	0.9	17.8	Low
ldlch	N/A	0	3316	N/A	N/A	N/A	N/A	N/A	N/A
iron	Numeric	210	3	92.57	39.07	88.0	10.0	364.0	Low
ferritin	Numeric	611	0	216.35	210.42	156.0	5.0	3316.0	Low
ldh	Numeric	290	1	182.069	61.4003	169.0	31.0	731.0	Low
hba1c	Numeric	85	3	6.32	1.25	6.0	3.9	18.6	Low
ace	Numeric	92	37	25.24	15.92	24.0	1.0	118.0	Low
vitb12	Numeric	696	3	394.38	221.43	343.0	69.0	2000.0	Low
folicac	Numeric	148	0	8.17	2.83	7.9	1.4	21.1	Low
tsh	Numeric	467	29	1.75	4.4	1.24	0.01	134.9	Low
vitd25	Numeric	399	14	17.24	9.24	15.5	1.9	75.6	Low
PTH	N/A	0	3316	N/A	N/A	N/A	N/A	N/A	N/A

hb	Numeric	94	0	13.83	1.49	13.9	7.9	18.5	Low
quick	Numeric	75	4	90.4	13.83	95.0	19.0	100.0	Low
aptt	Numeric	115	18	37.92	19.7	33.0	24.0	201.0	Low
fibrinog	Numeric	373	4	397.14	108.409	378.0	10.0	930.0	Low
at3	Numeric	88	32	97.085	13.48	97.0	40.0	147.0	Low
fii	Numeric	154	526	104.055	26.0024	107.0	9.0	199.0	Low
vwfag	Numeric	305	12	170.25	72.69	158.0	21.0	552.0	Low
ddimer	Numeric	279	31	0.63	1.075	0.36	0.0	21.0	Low
tpaant	Numeric	312	3	13.53	7.059	11.9	1.3	96.0	Low
crp	Numeric	425	5	1.0	2.036	0.4	0.01	28.24	Low
haptoglo	Numeric	361	11	158.62	76.6	149.0	8.0	1137.0	Low
eapoa1	Numeric	149	1	129.53	24.97	127.0	52.0	241.0	Low
eapoa2	Numeric	454	1	41.58	9.42	41.2	2.1	85.6	Low
eapob	Numeric	148	1	104.014	24.93	102.0	32.0	245.0	Low
eapoe	Numeric	220	1	8.98	3.34	8.6	0.0	39.0	Low
elpa	Numeric	478	2	28.67	34.093	16.0	0.0	236.0	Low
vldlch	Numeric	146	1	36.52	25.35	31.0	0.0	350.0	Low
vldltg	Numeric	360	1	123.98	108.99	99.0	10.0	2379.0	Low
ldlch	Numeric	198	1	116.204	34.37	113.0	15.0	361.0	Low
ldltg	Numeric	80	1	31.47	11.64	30.0	2.0	133.0	Low
supercrp	Numeric	1049	4	9.17	18.84	3.34	0.17	269.0	Low
cystatc	Numeric	186	4	1.0019	0.42	0.92	0.35	7.44	Low
age	Numeric	2417	0	62.63	10.62	63.58	17.25	92.101	Low
bmi	Numeric	1660	0	27.5	4.072	27.055	16.35	48.34	Low
ishypert	Numeric	2	0	0.31	0.46	0.0	0.0	1.0	Low
smoclass	Numeric	3	0	0.84	0.73	1.0	0.0	2.0	Low
miyn	Numeric	2	0	0.42	0.49	0.0	0.0	1.0	Low
pbnpl1	Numeric	1242	33	930.77	2179.35	294.0	5.0	35000.0	Low
death2010	Numeric	2	0	0.3	0.46	0.0	0.0	1.0	Low
TnThs	Numeric	849	74	101.93	426.83	11.0	1.5	6640.0	Low
Galectin3	Numeric	300	595	15.71	7.24	14.4	1.7	100.1	Low

Data Quality Handling Report

Feature Name	Var Type	Missing Count	Missing Percentage	Imputation Name	Imputation Description
ldlch	Numeric	3316	100	Missing Values Imputed	Imputed value: 0
PTH	Numeric	3316	100	Missing Values Imputed	Imputed value: 0
Galectin3	Numeric	737	22	Missing Values Imputed	Missing indicator treated as feature, Imputed value: 14.5

fii	Numeric	650	20	Missing Values Imputed	Missing indicator treated as feature, Imputed value: 107
dyspneyn	Numeric	153	5	Missing Values Imputed	Missing indicator treated as feature, Imputed value: 0
anginayn	Numeric	125	4	Missing Values Imputed	Missing indicator treated as feature, Imputed value: 1
TnThs	Numeric	99	3	Missing Values Imputed	Missing indicator treated as feature, Imputed value: 11
exercise	Numeric	68	2	Missing Values Imputed	Missing indicator treated as feature, Imputed value: 6
pbnpl1	Numeric	51	2	Missing Values Imputed	Missing indicator treated as feature, Imputed value: 293
immunyn	Numeric	48	1	Missing Values Imputed	Missing indicator treated as feature, Imputed value: 0
ace	Numeric	48	1	Missing Values Imputed	Missing indicator treated as feature, Imputed value: 24
tsh	Numeric	45	1	Missing Values Imputed	Missing indicator treated as feature, Imputed value: 1.24
rhythyn	Numeric	42	1	Missing Values Imputed	Missing indicator treated as feature, Imputed value: 0
at3	Numeric	36	1	Missing Values Imputed	Missing indicator treated as feature, Imputed value: 97
ddimer	Numeric	35	1	Missing Values Imputed	Missing indicator treated as feature, Imputed value: 0.35
aptt	Numeric	21	1	Missing Values Imputed	Missing indicator treated as feature, Imputed value: 33
vitd25	Numeric	17	1	Missing Values Imputed	Missing indicator treated as feature, Imputed value: 15.6
haptoglo	Numeric	16	0	Missing Values Imputed	Missing indicator treated as feature, Imputed value: 150
vwfag	Numeric	13	0	Missing Values Imputed	Missing indicator treated as feature, Imputed value: 158
venthrom	Numeric	8	0	Missing Values Imputed	Missing indicator treated as feature, Imputed value: 0
infectyn	Numeric	6	0	Missing Values Imputed	Missing indicator treated as feature, Imputed value: 0
crp	Numeric	6	0	Missing Values Imputed	Missing indicator treated as feature, Imputed value: 0.41
supercrp	Numeric	6	0	Missing Values Imputed	Missing indicator treated as feature, Imputed value: 3.39

cystatc	Numeric	6	0	Missing Values Imputed	Imputed value: 0.92
carosten	Numeric	4	0	Missing Values Imputed	Missing indicator treated as feature, Imputed value: 0
canceryn	Numeric	4	0	Missing Values Imputed	Missing indicator treated as feature, Imputed value: 0
iron	Numeric	4	0	Missing Values Imputed	Missing indicator treated as feature, Imputed value: 89
hba1c	Numeric	4	0	Missing Values Imputed	Missing indicator treated as feature, Imputed value: 6
vitb12	Numeric	4	0	Missing Values Imputed	Missing indicator treated as feature, Imputed value: 344
quick	Numeric	4	0	Missing Values Imputed	Missing indicator treated as feature, Imputed value: 95
fibrinog	Numeric	4	0	Missing Values Imputed	Missing indicator treated as feature, Imputed value: 377
crea	Numeric	3	0	Missing Values Imputed	Missing indicator treated as feature, Imputed value: 0.9
tpaant	Numeric	3	0	Missing Values Imputed	Missing indicator treated as feature, Imputed value: 11.9
elpa	Numeric	3	0	Missing Values Imputed	Missing indicator treated as feature, Imputed value: 16
urea	Numeric	2	0	Missing Values Imputed	Missing indicator treated as feature, Imputed value: 36
ldltg	Numeric	2	0	Missing Values Imputed	Missing indicator treated as feature, Imputed value: 30
uricacid	Numeric	1	0	Missing Values Imputed	Missing indicator treated as feature, Imputed value: 4.9
ldh	Numeric	1	0	Missing Values Imputed	Missing indicator treated as feature, Imputed value: 168
folicac	Numeric	1	0	Missing Values Imputed	Missing indicator treated as feature, Imputed value: 7.9
eapoa1	Numeric	1	0	Missing Values Imputed	Missing indicator treated as feature, Imputed value: 127
eapoa2	Numeric	1	0	Missing Values Imputed	Imputed value: 41.2
eapob	Numeric	1	0	Missing Values Imputed	Imputed value: 103
eapoe	Numeric	1	0	Missing Values Imputed	Imputed value: 8.6
vldlch	Numeric	1	0	Missing Values Imputed	Imputed value: 31
vldltg	Numeric	1	0	Missing Values Imputed	Imputed value: 99
ldlch	Numeric	1	0	Missing Values Imputed	Imputed value: 114
sex	Numeric	0	0	Missing Values Imputed	Imputed value: 1

cadyn	Numeric	0	0	Missing Values Imputed	Imputed value: 1
strokeyn	Numeric	0	0	Missing Values Imputed	Imputed value: 0
dm1yn	Numeric	0	0	Missing Values Imputed	Imputed value: 0
dm2yn	Numeric	0	0	Missing Values Imputed	Imputed value: 0
cmpyn	Numeric	0	0	Missing Values Imputed	Imputed value: 0
vdyn	Numeric	0	0	Missing Values Imputed	Imputed value: 0
ferritin	Numeric	0	0	Missing Values Imputed	Imputed value: 156
hb	Numeric	0	0	Missing Values Imputed	Imputed value: 13.9
age	Numeric	0	0	Missing Values Imputed	Imputed value: 63.5418
bmi	Numeric	0	0	Missing Values Imputed	Imputed value: 27.0602
ishypert	Numeric	0	0	Missing Values Imputed	Imputed value: 0
smoclass	Numeric	0	0	Missing Values Imputed	Imputed value: 1
miyn	Numeric	0	0	Missing Values Imputed	Imputed value: 0
death2010	Numeric	0	0	Missing Values Imputed	Imputed value: 0

Target: LPA-U

Data Partitioning Methodology

The modeling partitions were randomly selected using a stratified sample to preserve the distribution of the target for each partition.

Model Features and Summary Statistics

Feature Name	Var Type	Unique	Missing	Mean	Std Dev	Median	Min	Max	Target Leakage
sex	Categorical	3	1	N/A	N/A	N/A	N/A	N/A	Low
BMI cat	Categorical	3	107	N/A	N/A	N/A	N/A	N/A	Low
statin_pt0	Categorical	6	164	N/A	N/A	N/A	N/A	N/A	Low
statin_at0	Categorical	6	118	N/A	N/A	N/A	N/A	N/A	Low
ezetimibe	Categorical	5	2	N/A	N/A	N/A	N/A	N/A	Low
ezetimibe_pt0	Numeric	2	222	0.99	0.12	1.0	0.0	1.0	Low
ezetimibe_at0	Numeric	2	195	0.96	0.2008	1.0	0.0	1.0	Low
PSCK9	Categorical	4	2	N/A	N/A	N/A	N/A	N/A	Low
otherlipidpharm	Categorical	34	205	N/A	N/A	N/A	N/A	N/A	Low
bypass_surgery	Numeric	2	0	0.097	0.3	0.0	0.0	1.0	Low
COPD	Numeric	2	0	0.0103	0.101	0.0	0.0	1.0	Low
LVF	Text	100	106	N/A	N/A	N/A	N/A	N/A	N/A
LVFhireduced	Numeric	2	0	0.024	0.15	0.0	0.0	1.0	Low
ddysfct	Numeric	2	0	0.19	0.39	0.0	0.0	1.0	Low
aht	Numeric	2	0	0.6	0.49	1.0	0.0	1.0	Low
dm1	Numeric	2	0	0.0034	0.059	0.0	0.0	1.0	Low
HbA1c cat	Categorical	3	85	N/A	N/A	N/A	N/A	N/A	Low
pos_FA	Numeric	2	0	0.49	0.5	0.0	0.0	1.0	Low
earlycvevent	Numeric	2	0	0.031	0.17	0.0	0.0	1.0	Low
LDL_C	Numeric	5	139	2.85	2.0803	3.0	0.0	8.0	Low
FHscore	Numeric	13	1	2.53	2.59	2.0	0.0	13.0	Low
CHA2DS2_VASc_Score	Numeric	8	1	2.77	1.48	3.0	0.0	7.0	Low
SGLT2pt0	Numeric	2	0	0.017	0.13	0.0	0.0	1.0	Low
SGLT2at0	Numeric	2	0	0.0069	0.083	0.0	0.0	1.0	Low
1_alipo2	Numeric	51	181	50.34	15.26	48.0	25.0	91.0	Low
1_blipo2	Numeric	86	181	141.29	65.39	132.0	24.0	378.0	Low
1_LDL	Numeric	133	85	138.28	60.15	134.0	32.0	364.0	Low
1_LDL_categorical	Categorical	5	85	N/A	N/A	N/A	N/A	N/A	Low
LDLcatyn	Numeric	2	85	0.32	0.47	0.0	0.0	1.0	Low
1_HDL	Numeric	62	86	53.59	16.86	53.0	16.0	144.0	Low

1_TG2	Numeric	182	31	230.51	258.77	149.0	32.0	1928.0	Low
1_CK	Numeric	103	160	214.83	447.62	142.5	35.0	4972.0	Low
1_CK2	Numeric	45	236	203.96	567.73	92.0	46.0	4181.0	Low
1_LPAov50	Numeric	2	0	0.42	0.49	0.0	0.0	1.0	N/A
1_ApoB	Categorical	74	0	N/A	N/A	N/A	N/A	N/A	Low
1_CRP	Categorical	41	0	N/A	N/A	N/A	N/A	N/A	Low
2_NPPB_GFR	Numeric	43	245	280.22	377.95	102.0	12.0	1568.0	Low
3_LDL	Numeric	15	273	52.53	28.43	46.0	21.0	115.0	Low
3_TG2	Numeric	15	273	200.35	98.53	213.0	85.0	412.0	Low
3_TG2	Numeric	48	234	179.089	208.202	135.0	45.0	1601.0	Low
3_CK2	Numeric	32	255	157.89	100.29	143.0	46.0	564.0	Low
3_NPPB_GFR	Numeric	34	253	256.59	367.7	100.0	13.0	1543.0	Low
4_LDL2	Numeric	17	272	67.89	33.38	60.0	24.0	144.0	Low

Data Quality Handling Report

**Feature Name**	**Var Type**	**Missing Count**	**Missing Percentage**	**Imputation Name**	**Imputation Description**
3_TG	Numeric	342	94	Missing Values Imputed	Missing indicator treated as feature, Imputed value: 151
3_LDL	Numeric	341	94	Missing Values Imputed	Missing indicator treated as feature, Imputed value: 50
4_LDL2	Numeric	341	94	Missing Values Imputed	Missing indicator treated as feature, Imputed value: 60
3_CK	Numeric	317	87	Missing Values Imputed	Missing indicator treated as feature, Imputed value: 116
3_NPPB_GFR	Numeric	315	87	Missing Values Imputed	Missing indicator treated as feature, Imputed value: 111
2_NPPB_GFR	Numeric	310	85	Missing Values Imputed	Missing indicator treated as feature, Imputed value: 127
1_CK2	Numeric	295	81	Missing Values Imputed	Missing indicator treated as feature, Imputed value: 92
3_TG2	Numeric	288	79	Missing Values Imputed	Missing indicator treated as feature, Imputed value: 128
ezetimibe_pt0	Numeric	284	78	Missing Values Imputed	Missing indicator treated as feature, Imputed value: 1
otherlipidpharm	Categorical	257	71	One-Hot Encoding	Missing indicator treated as feature
ezetimibe_at0	Numeric	247	68	Missing Values Imputed	Missing indicator treated as feature, Imputed value: 1
1_alipo2	Numeric	237	65	Missing Values Imputed	Missing indicator treated as feature, Imputed value: 47

1_blipo2	Numeric	237	65	Missing Values Imputed	Imputed value: 134
statin_pt0	Categorical	206	57	One-Hot Encoding	Missing indicator treated as feature
1_CK	Numeric	205	56	Missing Values Imputed	Missing indicator treated as feature, Imputed value: 142
LDL_C	Numeric	171	47	Missing Values Imputed	Missing indicator treated as feature, Imputed value: 3
statin_at0	Categorical	149	41	One-Hot Encoding	Missing indicator treated as feature
BMI cat	Categorical	127	35	One-Hot Encoding	Missing indicator treated as feature
1_HDL	Numeric	111	31	Missing Values Imputed	Missing indicator treated as feature, Imputed value: 50
1_LDL_categorical	Categorical	110	30	One-Hot Encoding	Missing indicator treated as feature
1_LDL	Numeric	110	30	Missing Values Imputed	Missing indicator treated as feature, Imputed value: 132
LDLcatyn	Numeric	110	30	Missing Values Imputed	Imputed value: 0
HbA1c cat	Categorical	109	30	One-Hot Encoding	Missing indicator treated as feature
1_TG2	Numeric	47	13	Missing Values Imputed	Missing indicator treated as feature, Imputed value: 154
sex	Categorical	2	1	One-Hot Encoding	Missing values treated as infrequent
ezetimibe	Categorical	2	1	One-Hot Encoding	Missing values treated as infrequent
PSCK9	Categorical	2	1	One-Hot Encoding	Missing values treated as infrequent
FHscore	Numeric	1	0	Missing Values Imputed	Missing indicator treated as feature, Imputed value: 2
CHA2DS2_VASc_Score	Numeric	1	0	Missing Values Imputed	Missing indicator treated as feature, Imputed value: 3
1_ApoB	Categorical	0	0	One-Hot Encoding	Missing values treated as infrequent
1_CRP	Categorical	0	0	One-Hot Encoding	Missing values treated as infrequent
bypass_surgery	Numeric	0	0	Missing Values Imputed	Imputed value: 0
COPD	Numeric	0	0	Missing Values Imputed	Imputed value: 0
LVFhireduced	Numeric	0	0	Missing Values Imputed	Imputed value: 0
ddysfct	Numeric	0	0	Missing Values Imputed	Imputed value: 0

aht	Numeric	0	0	Missing Values Imputed	Imputed value: 1
dm1	Numeric	0	0	Missing Values Imputed	Imputed value: 0
pos_FA	Numeric	0	0	Missing Values Imputed	Imputed value: 0
earlycvevent	Numeric	0	0	Missing Values Imputed	Imputed value: 0
SGLT2pt0	Numeric	0	0	Missing Values Imputed	Imputed value: 0
SGLT2at0	Numeric	0	0	Missing Values Imputed	Imputed value: 0

Target: CAD-U

Data Partitioning Methodology

The modeling partitions were randomly selected using a stratified sample to preserve the distribution of the target for each partition.

Model Features and Summary Statistics

Feature Name	Var Type	Unique	Missing	Mean	Std Dev	Median	Min	Max	Target Leakage
sex	Categorical	4	2	N/A	N/A	N/A	N/A	N/A	Low
age	Numeric	66	4	56.22	13.97	57.0	13.0	88.0	Low
BMI	Numeric	119	140	27.89	5.61	27.3	18.8	57.8	Low
HbA1c	Numeric	39	108	5.83	0.809	5.7	4.4	8.8	Low
HbA1c_at0	Numeric	27	294	20.25	92.98	6.0	4.4	637.0	Low
1_LDL	Numeric	153	107	139.29	60.19	133.5	32.0	364.0	Low
1_HDL	Numeric	65	108	52.52	17.35	49.0	18.0	144.0	Low
1_chol	Numeric	151	108	230.91	73.37	224.0	83.0	471.0	Low
1_TG2	Numeric	176	108	262.606	304.014	164.0	38.0	2418.0	Low
1_CK	Numeric	105	204	221.32	448.021	147.0	20.0	4972.0	Low
1_LPA	Numeric	223	41	50.84	44.41	40.4	2.0	252.0	Low
1_ApoA1	Numeric	134	141	153.48	31.14	148.0	95.4	285.4	Low
1_ApoB	Categorical	81	0	N/A	N/A	N/A	N/A	N/A	Low
1_hsCRP	Categorical	101	0	N/A	N/A	N/A	N/A	N/A	Low
1_NPPB	Numeric	116	169	145.61	235.9	66.0	5.0	2024.0	Low
1_homocys	Categorical	121	0	N/A	N/A	N/A	N/A	N/A	Low
1_vitD	Categorical	45	0	N/A	N/A	N/A	N/A	N/A	Low
2_LDL2	Numeric	89	197	84.19	48.37	72.5	11.0	248.0	Low
2_HDL2	Numeric	59	202	56.099	16.33	55.0	21.0	106.0	Low
2_chol2	Numeric	96	200	159.6	55.23	146.0	57.0	340.0	Low
2_TG2	Numeric	104	197	163.89	165.308	120.5	29.0	1466.0	Low
2_CK2	Numeric	60	271	148.809	105.22	118.0	50.0	679.0	Low
2_LPA	Numeric	49	288	67.35	50.65	65.2	4.9	237.0	Low
2_ApoA1	Numeric	23	316	172.36	33.78	169.0	105.3	236.8	Low
2_ApoB	Numeric	20	316	64.17	27.71	60.0	29.6	161.0	Low
2_CRP	Categorical	47	0	N/A	N/A	N/A	N/A	N/A	Low
2_NPPB	Categorical	47	0	N/A	N/A	N/A	N/A	N/A	Low
2_homocys	Categorical	37	0	N/A	N/A	N/A	N/A	N/A	Low
2_vitD	Numeric	32	287	24.94	10.42	25.0	6.0	51.0	Low

Data Quality Handling Report

Feature Name	Var Type	Missing Count	Missing Percentage	Imputation Name	Imputation Description
2_ApoA1	Numeric	393	93	Missing Values Imputed	Missing indicator treated as feature, Imputed value: 168
2_ApoB	Numeric	393	93	Missing Values Imputed	Imputed value: 68.9
HbA1c_at0	Numeric	371	88	Missing Values Imputed	Missing indicator treated as feature, Imputed value: 6.1
2_LPA	Numeric	362	86	Missing Values Imputed	Missing indicator treated as feature, Imputed value: 53.2
2_vitD	Numeric	360	85	Missing Values Imputed	Missing indicator treated as feature, Imputed value: 25
2_CK2	Numeric	341	81	Missing Values Imputed	Missing indicator treated as feature, Imputed value: 127
2_HDL2	Numeric	259	61	Missing Values Imputed	Missing indicator treated as feature, Imputed value: 54
2_chol2	Numeric	256	61	Missing Values Imputed	Missing indicator treated as feature, Imputed value: 144
2_LDL2	Numeric	253	60	Missing Values Imputed	Missing indicator treated as feature, Imputed value: 74
2_TG2	Numeric	253	60	Missing Values Imputed	Missing indicator treated as feature, Imputed value: 122
1_CK	Numeric	245	58	Missing Values Imputed	Missing indicator treated as feature, Imputed value: 142
1_NPPB	Numeric	210	50	Missing Values Imputed	Missing indicator treated as feature, Imputed value: 70
1_ApoA1	Numeric	179	42	Missing Values Imputed	Missing indicator treated as feature, Imputed value: 148
BMI	Numeric	170	40	Missing Values Imputed	Missing indicator treated as feature, Imputed value: 27.2
1_HDL	Numeric	145	34	Missing Values Imputed	Missing indicator treated as feature, Imputed value: 49
1_chol	Numeric	145	34	Missing Values Imputed	Imputed value: 225
1_TG	Numeric	145	34	Missing Values Imputed	Imputed value: 163
1_LDL	Numeric	143	34	Missing Values Imputed	Missing indicator treated as feature, Imputed value: 133
HbA1c	Numeric	138	33	Missing Values Imputed	Missing indicator treated as feature, Imputed value: 5.6
1_LPA	Numeric	60	14	Missing Values Imputed	Missing indicator treated as feature, Imputed value: 40.2

age	Numeric	6	1	Missing Values Imputed	Missing indicator treated as feature, Imputed value: 57
sex	Categorical	3	1	One-Hot Encoding	Missing values treated as infrequent
1_ApoB	Categorical	0	0	One-Hot Encoding	Missing values treated as infrequent
1_hsCRP	Categorical	0	0	One-Hot Encoding	Missing values treated as infrequent
1_homocys	Categorical	0	0	One-Hot Encoding	Missing values treated as infrequent
1_vitD	Categorical	0	0	One-Hot Encoding	Missing values treated as infrequent
2_hsCRP	Categorical	0	0	One-Hot Encoding	Missing values ignored
2_NPPB	Categorical	0	0	One-Hot Encoding	Missing values ignored
2_homocys	Categorical	0	0	One-Hot Encoding	Missing values ignored

Target: Early CV conditions-U

Data Partitioning Methodology

The modeling partitions were randomly selected using a stratified sample to preserve the distribution of the target for each partition.

Model Features and Summary Statistics

Feature Name	Var Type	Unique	Missing	Mean	Std Dev	Median	Min	Max	Target Leakage
1_LDLbetafract	Numeric	92	230	48.8	13.06	49.1	8.4	78.6	Low
1_HDLalphafract	Numeric	94	230	24.14	9.28	24.1	6.9	47.8	Low
1_VLDLprebetafract	Numeric	99	230	27.064	16.12	22.8	1.6	73.7	Low
1_blipo	Categorical	63	0	N/A	N/A	N/A	N/A	N/A	Low
1_alipo	Categorical	62	0	N/A	N/A	N/A	N/A	N/A	Low
1_preblipo	Categorical	56	0	N/A	N/A	N/A	N/A	N/A	Low
1_alipo2	Numeric	45	233	48.104	14.43	45.0	25.0	91.0	Low
1_blipo2	Numeric	82	233	141.97	69.59	131.5	24.0	378.0	Low
1_LDL	Numeric	144	118	138.16	59.82	132.0	33.0	364.0	Low
1_HDL	Numeric	64	120	51.54	17.18	48.0	16.0	144.0	Low
1_chol	Numeric	153	120	231.34	76.91	224.0	79.0	540.0	Low
1_TG2	Numeric	168	120	276.39	338.75	162.0	38.0	2418.0	Low
1_LDL2	Numeric	122	130	130.7	55.62	122.0	25.0	313.0	Low
1_HDL2	Numeric	67	133	53.19	18.13	50.0	16.0	141.0	Low
1_chol2	Numeric	179	58	217.92	72.37	212.0	76.0	452.0	Low
1_TG2	Numeric	193	59	245.59	329.16	146.5	37.0	3414.0	Low
1_CK	Numeric	100	202	240.53	520.208	138.0	20.0	4972.0	Low
1_CK2	Numeric	48	277	197.58	533.015	94.5	43.0	4181.0	Low
1_LPA	Categorical	230	0	N/A	N/A	N/A	N/A	N/A	Low
1_ApoA1	Numeric	130	145	152.86	31.71	146.0	85.9	285.4	Low
1_ApoB	Categorical	80	0	N/A	N/A	N/A	N/A	N/A	Low
1_hsCRP	Categorical	105	0	N/A	N/A	N/A	N/A	N/A	Low
1_CRP	Categorical	58	0	N/A	N/A	N/A	N/A	N/A	Low
1_NPPB	Numeric	123	168	159.56	259.99	70.0	5.0	2024.0	Low
1_NPPB_GFR	Numeric	130	170	290.047	646.901	109.0	9.0	5208.0	Low
1_homocys	Categorical	116	0	N/A	N/A	N/A	N/A	N/A	Low
1_vitD	Categorical	42	0	N/A	N/A	N/A	N/A	N/A	Low
2_LDLbetafract	Numeric	9	330	37.42	11.34	36.4	20.2	55.2	Low
2_HDLalphafract	Numeric	9	330	27.44	10.69	32.6	10.3	42.9	Low
2_VLDLprebetafract	Numeric	9	330	35.13	14.29	30.6	20.7	60.3	Low

2_blipo	Categorical	14	0	N/A	N/A	N/A	N/A	N/A	Low
2_alipo	Categorical	14	0	N/A	N/A	N/A	N/A	N/A	Low
2_preblipo	Categorical	13	0	N/A	N/A	N/A	N/A	N/A	Low
2_alipo2	Numeric	7	330	51.44	16.53	51.0	30.0	92.0	Low
2_blipo2	Numeric	8	330	87.44	51.7	74.0	34.0	222.0	Low
2_LDL	Numeric	35	301	70.58	38.7	66.0	17.0	196.0	Low
2_HDL	Numeric	27	304	55.8	16.94	56.0	22.0	96.0	Low
2_chol	Categorical	35	0	N/A	N/A	N/A	N/A	N/A	Low
2_TG2	Categorical	37	0	N/A	N/A	N/A	N/A	N/A	Low
2_LDL2	Numeric	87	207	85.71	47.68	76.5	11.0	248.0	Low
2_HDL2	Numeric	59	213	55.5	16.36	54.0	21.0	106.0	Low
2_chol2	Numeric	96	209	160.37	55.49	146.5	63.0	340.0	Low
2_TG2	Numeric	100	207	168.35	170.0	126.5	29.0	1466.0	Low
2_CK	Numeric	65	270	159.65	122.202	125.0	38.0	679.0	Low
2_CK2	Numeric	23	315	108.083	48.61	104.5	49.0	273.0	Low
2_LPA	Categorical	48	0	N/A	N/A	N/A	N/A	N/A	Low
2_ApoA1	Numeric	23	314	163.72	33.43	158.0	105.3	236.8	Low
2_ApoB	Numeric	19	314	65.66	25.56	66.9	29.6	161.0	Low
2_hsCRP	Categorical	48	0	N/A	N/A	N/A	N/A	N/A	Low
2_CRP	Categorical	22	0	N/A	N/A	N/A	N/A	N/A	Low
2_NPPB	Categorical	56	0	N/A	N/A	N/A	N/A	N/A	Low
2_NPPB_GFR	Numeric	57	279	279.33	371.67	140.5	12.0	1568.0	Low
2_homocys	Categorical	38	0	N/A	N/A	N/A	N/A	N/A	Low
2_vitD	Numeric	30	288	24.86	10.088	24.0	6.0	51.0	Low
3_LDLbetafract	Numeric	2	337	34.85	4.25	34.85	30.6	39.1	Low
3_HDLalphafract	Numeric	2	337	45.75	4.15	45.75	41.6	49.9	Low
3_VLDLprebetafract	Numeric	2	337	19.4	0.1	19.4	19.3	19.5	Low
3_blipo	Categorical	19	0	N/A	N/A	N/A	N/A	N/A	Low
3_alipo	Categorical	19	0	N/A	N/A	N/A	N/A	N/A	Low
3_preblipo	Categorical	18	0	N/A	N/A	N/A	N/A	N/A	Low
3_alipo2	Numeric	2	337	65.0	2.0	65.0	63.0	67.0	Low
3_blipo2	Numeric	2	337	60.5	11.5	60.5	49.0	72.0	Low
3_LDL	Numeric	23	314	59.32	30.17	50.0	21.0	126.0	Low
3_HDL	Numeric	22	314	58.8	14.0057	56.0	42.0	108.0	Low
3_chol	Numeric	21	316	137.7	35.107	142.0	80.0	203.0	Low
3_TG2	Numeric	24	314	184.52	92.51	165.0	43.0	412.0	Low
3_LDL2	Numeric	56	265	72.47	40.9	67.0	17.0	212.0	Low
3_HDL2	Numeric	40	268	54.63	15.809	53.0	29.0	109.0	Low

3_chol2	Numeric	57	262	148.95	52.29	142.0	78.0	299.0	Low
3_TG2	Numeric	64	264	182.67	192.11	142.0	48.0	1601.0	Low
3_CK	Numeric	38	296	167.65	131.43	119.0	44.0	664.0	Low
3_CK2	Numeric	16	321	107.83	50.39	89.0	51.0	217.0	Low
3_LPA	Categorical	35	0	N/A	N/A	N/A	N/A	N/A	Low
3_ApoA1	Numeric	13	326	166.708	24.14	172.9	121.0	207.1	Low
3_ApoB	Numeric	13	326	56.85	23.28	56.0	24.6	96.7	Low
3_hsCRP	Categorical	37	0	N/A	N/A	N/A	N/A	N/A	Low
3_CRP	Categorical	19	0	N/A	N/A	N/A	N/A	N/A	Low
3_NPPB	Numeric	41	295	239.045	341.8	118.5	20.0	2092.0	Low
3_NPPB_GFR	Numeric	43	292	270.28	348.67	110.0	13.0	1543.0	Low
3_homocys	Categorical	39	0	N/A	N/A	N/A	N/A	N/A	Low
3_vitD	Numeric	27	294	30.13	16.26	27.8	7.0	105.0	Low
4_LDLbetafract	Categorical	1	0	N/A	N/A	N/A	N/A	N/A	N/A
4_HDLalphafract	Categorical	1	0	N/A	N/A	N/A	N/A	N/A	Low
4_VLDLprebetafract	Categorical	1	0	N/A	N/A	N/A	N/A	N/A	N/A
4_blipo	Categorical	3	0	N/A	N/A	N/A	N/A	N/A	Low
4_alipo	Categorical	3	0	N/A	N/A	N/A	N/A	N/A	Low
4_preblipo	Categorical	3	0	N/A	N/A	N/A	N/A	N/A	Low
4_alipo2	Categorical	1	0	N/A	N/A	N/A	N/A	N/A	N/A
4_blipo2	Categorical	1	0	N/A	N/A	N/A	N/A	N/A	N/A
4_LDL	Numeric	5	334	65.4	23.95	64.0	34.0	97.0	Low
4_HDL	Numeric	4	335	66.3	22.63	62.0	39.2	102.0	Low
4_chol	Numeric	5	334	152.6	24.98	167.0	113.0	179.0	Low
4_TG2	Numeric	5	334	160.4	131.91	86.0	46.0	410.0	Low
4_LDL2	Numeric	21	317	76.23	41.0	62.0	24.0	188.0	Low
4_HDL2	Numeric	16	318	57.19	17.95	59.0	29.0	122.0	Low
4_chol2	Numeric	21	317	146.59	38.96	135.0	95.0	239.0	Low
4_TG2	Categorical	24	0	N/A	N/A	N/A	N/A	N/A	Low
4_CK	Numeric	7	331	184.5	123.69	142.5	60.0	494.0	Low
4_CK2	Categorical	6	0	N/A	N/A	N/A	N/A	N/A	Low
4_LPA	Categorical	9	0	N/A	N/A	N/A	N/A	N/A	Low
4_ApoA1	Numeric	2	337	153.5	2.5	153.5	151.0	156.0	Low
4_ApoB	Numeric	2	337	345.0	265.0	345.0	80.0	610.0	Low
4_hsCRP	Categorical	12	0	N/A	N/A	N/A	N/A	N/A	Low
4_CRP	Categorical	4	0	N/A	N/A	N/A	N/A	N/A	Low
4_NPPB	Categorical	15	0	N/A	N/A	N/A	N/A	N/A	Low
4_NPPB_GFR	Numeric	14	325	312.21	310.26	182.0	38.0	1075.0	Low

4_homocys	Categorical	12	0	N/A	N/A	N/A	N/A	N/A	Low
4_vitD	Numeric	9	328	26.45	10.43	27.0	13.0	46.0	Low
5_LDLbetafract	Categorical	1	0	N/A	N/A	N/A	N/A	N/A	N/A
5_HDLalphafract	Categorical	1	0	N/A	N/A	N/A	N/A	N/A	N/A
5_VLDLprebetafract	Categorical	1	0	N/A	N/A	N/A	N/A	N/A	N/A
5_blipo	Categorical	2	0	N/A	N/A	N/A	N/A	N/A	Low
5_alipo	Categorical	2	0	N/A	N/A	N/A	N/A	N/A	Low
5_preblipo	Categorical	2	0	N/A	N/A	N/A	N/A	N/A	Low
5_alipo2	Categorical	1	0	N/A	N/A	N/A	N/A	N/A	N/A
5_blipo2	Categorical	1	0	N/A	N/A	N/A	N/A	N/A	N/A
5_LDL	Numeric	2	337	37.5	14.5	37.5	23.0	52.0	Low
5_HDL	Numeric	2	337	90.5	2.5	90.5	88.0	93.0	Low
5_chol	Numeric	2	337	148.0	10.0	148.0	138.0	158.0	Low
5_TG2	Numeric	2	337	79.5	42.5	79.5	37.0	122.0	Low
5_LDL2	Numeric	10	329	64.4	22.96	53.5	46.0	123.0	Low
5_HDL2	Numeric	8	331	53.38	16.109	51.5	31.0	89.0	Low
5_chol2	Numeric	9	330	138.33	34.97	123.0	91.0	205.0	Low
5_TG2	Numeric	9	330	163.78	69.79	172.0	44.0	264.0	Low
5_CK	Numeric	5	334	180.2	95.48	132.0	85.0	357.0	Low
5_CK2	Numeric	2	337	192.0	87.0	192.0	105.0	279.0	Low
5_LPA	Categorical	4	0	N/A	N/A	N/A	N/A	N/A	Low
5_ApoA1	Boolean	1	338	228.6	0.0	228.6	228.6	228.6	Low
5_ApoB	Numeric	2	337	46.8	6.8	46.8	40.0	53.6	Low
5_hsCRP	Categorical	6	0	N/A	N/A	N/A	N/A	N/A	Low
5_CRP	Categorical	4	0	N/A	N/A	N/A	N/A	N/A	Low
5_NPPB	Numeric	4	335	111.5	75.19	79.5	51.0	236.0	Low
5_NPPB_GFR	Numeric	6	333	374.5	354.12	186.5	82.0	1018.0	Low
5_homocys	Categorical	8	0	N/A	N/A	N/A	N/A	N/A	Low
5_vitD	Numeric	5	333	30.17	12.94	29.0	14.0	55.0	Low
6_LDLbetafract	Categorical	1	0	N/A	N/A	N/A	N/A	N/A	N/A
6_HDLalphafract	Categorical	1	0	N/A	N/A	N/A	N/A	N/A	N/A
6_VLDLprebetafract	Categorical	1	0	N/A	N/A	N/A	N/A	N/A	N/A
6_blipo	Categorical	3	0	N/A	N/A	N/A	N/A	N/A	Low
6_alipo	Categorical	3	0	N/A	N/A	N/A	N/A	N/A	Low
6_preblipo	Categorical	3	0	N/A	N/A	N/A	N/A	N/A	Low
6_alipo2	Categorical	1	0	N/A	N/A	N/A	N/A	N/A	N/A
6_blipo2	Categorical	1	0	N/A	N/A	N/A	N/A	N/A	N/A
6_LDL	Numeric	2	337	56.0	32.0	56.0	24.0	88.0	Low

6_HDL	Boolean	1	337	53.0	0.0	53.0	53.0	53.0	Low
6_chol	Numeric	2	337	126.0	35.0	126.0	91.0	161.0	Low
6_TG2	Numeric	2	337	138.0	24.0	138.0	114.0	162.0	Low
6_LDL2	Numeric	6	333	64.17	27.44	69.5	24.0	99.0	Low
6_HDL2	Numeric	4	334	53.0	12.13	51.0	35.0	73.0	Low
6_chol2	Numeric	6	333	127.67	33.89	132.0	86.0	168.0	Low
6_TG2	Numeric	5	334	113.6	33.0006	116.0	58.0	161.0	Low
6_CK	Numeric	4	335	154.0	68.22	151.0	68.0	246.0	Low
6_CK2	Boolean	1	338	68.0	0.0	68.0	68.0	68.0	Low
6_LPA	Boolean	1	338	47.0	0.0	47.0	47.0	47.0	Low
6_ApoA1	Boolean	1	338	154.2	0.0	154.2	154.2	154.2	Low
6_ApoB	Boolean	1	338	28.8	0.0	28.8	28.8	28.8	Low
6_hsCRP	Categorical	4	0	N/A	N/A	N/A	N/A	N/A	Low
6_CRP	Categorical	4	0	N/A	N/A	N/A	N/A	N/A	Low
6_NPPB	Numeric	3	336	145.0	69.88	183.0	47.0	205.0	Low
6_NPPB_GFR	Numeric	4	335	311.5	204.14	291.5	52.0	611.0	Low
6_homocys	Categorical	4	0	N/A	N/A	N/A	N/A	N/A	Low
6_vitD	Numeric	3	336	32.0	6.68	30.0	25.0	41.0	Low

Data Quality Handling Report

Feature Name	Var Type	Missing Count	Missing Percentage	Imputation Name	Imputation Description
4_ApoA1	Numeric	270	100	Missing Values Imputed	Imputed value: 155
4_ApoB	Numeric	270	100	Missing Values Imputed	Imputed value: 79
5_ApoA1	Numeric	270	100	Missing Values Imputed	Imputed value: 227.6
6_CK2	Numeric	270	100	Missing Values Imputed	Imputed value: 67
6_LPA	Numeric	270	100	Missing Values Imputed	Imputed value: 46
6_ApoA1	Numeric	270	100	Missing Values Imputed	Imputed value: 153.2
6_ApoB	Numeric	270	100	Missing Values Imputed	Imputed value: 27.8
3_LDLbetafract	Numeric	269	99	Missing Values Imputed	Imputed value: 39.1
3_HDLalphafract	Numeric	269	99	Missing Values Imputed	Imputed value: 49.9
3_VLDLprebetafract	Numeric	269	99	Missing Values Imputed	Imputed value: 19.5
3_alipo2	Numeric	269	99	Missing Values Imputed	Imputed value: 67
3_blipo2	Numeric	269	99	Missing Values Imputed	Imputed value: 72
5_LDL	Numeric	269	99	Missing Values Imputed	Imputed value: 52
5_HDL	Numeric	269	99	Missing Values Imputed	Imputed value: 93
5_chol	Numeric	269	99	Missing Values Imputed	Imputed value: 158
5_TG	Numeric	269	99	Missing Values Imputed	Imputed value: 122

5_CK2	Numeric	269	99	Missing Values Imputed	Imputed value: 279
5_ApoB	Numeric	269	99	Missing Values Imputed	Imputed value: 53.6
6_LDL	Numeric	269	99	Missing Values Imputed	Imputed value: 88
6_HDL	Numeric	269	99	Missing Values Imputed	Imputed value: 52
6_chol	Numeric	269	99	Missing Values Imputed	Imputed value: 161
6_TG	Numeric	269	99	Missing Values Imputed	Imputed value: 162
6_NPPB	Numeric	269	99	Missing Values Imputed	Imputed value: 205
6_NPPB_GFR	Numeric	269	99	Missing Values Imputed	Imputed value: 358
6_TG2	Numeric	268	99	Missing Values Imputed	Imputed value: 123
6_CK	Numeric	268	99	Missing Values Imputed	Imputed value: 114
6_vitD	Numeric	268	99	Missing Values Imputed	Imputed value: 30
4_HDL	Numeric	267	99	Missing Values Imputed	Imputed value: 63
5_NPPB	Numeric	267	99	Missing Values Imputed	Imputed value: 106
6_LDL2	Numeric	267	99	Missing Values Imputed	Imputed value: 84
6_HDL2	Numeric	267	99	Missing Values Imputed	Imputed value: 55
6_Chol2	Numeric	267	99	Missing Values Imputed	Imputed value: 156
4_LDL	Numeric	266	98	Missing Values Imputed	Imputed value: 64
4_chol	Numeric	266	98	Missing Values Imputed	Imputed value: 167
4_TG	Numeric	266	98	Missing Values Imputed	Imputed value: 86
5_CK	Numeric	266	98	Missing Values Imputed	Imputed value: 132
5_NPPB_GFR	Numeric	266	98	Missing Values Imputed	Imputed value: 141
5_vitD	Numeric	265	98	Missing Values Imputed	Imputed value: 33
2_LDLbetafract	Numeric	264	97	Missing Values Imputed	Imputed value: 36.4
2_HDLalphafract	Numeric	264	97	Missing Values Imputed	Imputed value: 32.6
2_VLDLprebetafract	Numeric	264	97	Missing Values Imputed	Imputed value: 30.6
2_alipo2	Numeric	264	97	Missing Values Imputed	Imputed value: 54
2_blipo2	Numeric	264	97	Missing Values Imputed	Imputed value: 74
4_CK	Numeric	264	97	Missing Values Imputed	Imputed value: 147
5_HDL2	Numeric	264	97	Missing Values Imputed	Imputed value: 52
5_LDL2	Numeric	263	97	Missing Values Imputed	Imputed value: 54
5_Chol2	Numeric	263	97	Missing Values Imputed	Imputed value: 161
5_TG2	Numeric	263	97	Missing Values Imputed	Imputed value: 175
4_NPPB_GFR	Numeric	260	96	Missing Values Imputed	Missing indicator treated as feature, Imputed value: 116
4_vitD	Numeric	260	96	Missing Values Imputed	Missing indicator treated as feature, Imputed value: 27
3_ApoA1	Numeric	258	95	Missing Values Imputed	Missing indicator treated as feature, Imputed value: 172.9

3_ApoB	Numeric	258	95	Missing Values Imputed	Imputed value: 56
3_CK2	Numeric	254	94	Missing Values Imputed	Missing indicator treated as feature, Imputed value: 84
4_HDL2	Numeric	253	93	Missing Values Imputed	Missing indicator treated as feature, Imputed value: 59
4_Chol2	Numeric	253	93	Missing Values Imputed	Imputed value: 139
2_ApoA1	Numeric	252	93	Missing Values Imputed	Missing indicator treated as feature, Imputed value: 158
2_ApoB	Numeric	252	93	Missing Values Imputed	Imputed value: 60.3
4_LDL2	Numeric	252	93	Missing Values Imputed	Missing indicator treated as feature, Imputed value: 74
2_CK2	Numeric	249	92	Missing Values Imputed	Missing indicator treated as feature, Imputed value: 110
3_chol	Numeric	249	92	Missing Values Imputed	Missing indicator treated as feature, Imputed value: 142
3_LDL	Numeric	247	91	Missing Values Imputed	Missing indicator treated as feature, Imputed value: 50
3_HDL	Numeric	247	91	Missing Values Imputed	Imputed value: 57
3_TG	Numeric	247	91	Missing Values Imputed	Imputed value: 165
2_HDL	Numeric	242	89	Missing Values Imputed	Missing indicator treated as feature, Imputed value: 56
2_LDL	Numeric	240	89	Missing Values Imputed	Missing indicator treated as feature, Imputed value: 64
3_CK	Numeric	234	86	Missing Values Imputed	Missing indicator treated as feature, Imputed value: 112
3_NPPB	Numeric	231	85	Missing Values Imputed	Missing indicator treated as feature, Imputed value: 106
2_vitD	Numeric	230	85	Missing Values Imputed	Missing indicator treated as feature, Imputed value: 24
3_NPPB_GFR	Numeric	229	84	Missing Values Imputed	Missing indicator treated as feature, Imputed value: 110
3_vitD	Numeric	229	84	Missing Values Imputed	Missing indicator treated as feature, Imputed value: 28
2_NPPB_GFR	Numeric	225	83	Missing Values Imputed	Missing indicator treated as feature, Imputed value: 116
1_CK2	Numeric	220	81	Missing Values Imputed	Missing indicator treated as feature, Imputed value: 92
2_CK	Numeric	216	80	Missing Values Imputed	Missing indicator treated as feature, Imputed value: 125

3_HDL2	Numeric	209	77	Missing Values Imputed	Missing indicator treated as feature, Imputed value: 54
3_TG2	Numeric	208	77	Missing Values Imputed	Missing indicator treated as feature, Imputed value: 147
3_LDL2	Numeric	207	76	Missing Values Imputed	Missing indicator treated as feature, Imputed value: 71
3_Chol2	Numeric	206	76	Missing Values Imputed	Missing indicator treated as feature, Imputed value: 147
1_alipo2	Numeric	186	69	Missing Values Imputed	Missing indicator treated as feature, Imputed value: 44
1_blipo2	Numeric	186	69	Missing Values Imputed	Imputed value: 135
1_LDLbetafract	Numeric	182	67	Missing Values Imputed	Missing indicator treated as feature, Imputed value: 48.9
1_HDLalphafract	Numeric	182	67	Missing Values Imputed	Imputed value: 23.7
1_VLDLprebetafract	Numeric	182	67	Missing Values Imputed	Imputed value: 22.4
2_HDL2	Numeric	170	63	Missing Values Imputed	Missing indicator treated as feature, Imputed value: 54
1_CK	Numeric	167	62	Missing Values Imputed	Missing indicator treated as feature, Imputed value: 128
2_chol2	Numeric	166	61	Missing Values Imputed	Missing indicator treated as feature, Imputed value: 151
2_LDL2	Numeric	165	61	Missing Values Imputed	Missing indicator treated as feature, Imputed value: 77
2_TG2	Numeric	165	61	Missing Values Imputed	Missing indicator treated as feature, Imputed value: 128
1_NPPB_GFR	Numeric	138	51	Missing Values Imputed	Missing indicator treated as feature, Imputed value: 105
1_NPPB	Numeric	135	50	Missing Values Imputed	Missing indicator treated as feature, Imputed value: 73
1_ApoA1	Numeric	121	45	Missing Values Imputed	Missing indicator treated as feature, Imputed value: 147.4
1_HDL2	Numeric	107	39	Missing Values Imputed	Missing indicator treated as feature, Imputed value: 51
1_LDL2	Numeric	103	38	Missing Values Imputed	Missing indicator treated as feature, Imputed value: 127
1_HDL	Numeric	98	36	Missing Values Imputed	Missing indicator treated as feature, Imputed value: 48
1_chol	Numeric	98	36	Missing Values Imputed	Imputed value: 225
1_TG	Numeric	98	36	Missing Values Imputed	Imputed value: 164

1_LDL	Numeric	97	36	Missing Values Imputed	Missing indicator treated as feature, Imputed value: 135
1_TG2	Numeric	49	18	Missing Values Imputed	Missing indicator treated as feature, Imputed value: 154
1_Chol2	Numeric	48	18	Missing Values Imputed	Missing indicator treated as feature, Imputed value: 213
1_blipo	Categorical	0	0	Ordinal encoding of categorical variables	Imputed value: -2
1_alipo	Categorical	0	0	Ordinal encoding of categorical variables	Imputed value: -2
1_preblipo	Categorical	0	0	Ordinal encoding of categorical variables	Imputed value: -2
1_LPA	Categorical	0	0	Ordinal encoding of categorical variables	Imputed value: -2
1_ApoB	Categorical	0	0	Ordinal encoding of categorical variables	Imputed value: -2
1_hsCRP	Categorical	0	0	Ordinal encoding of categorical variables	Imputed value: -2
1_CRP	Categorical	0	0	Ordinal encoding of categorical variables	Imputed value: -2
1_homocys	Categorical	0	0	Ordinal encoding of categorical variables	Imputed value: -2
1_vitD	Categorical	0	0	Ordinal encoding of categorical variables	Imputed value: -2
2_blipo	Categorical	0	0	Ordinal encoding of categorical variables	Imputed value: -2
2_alipo	Categorical	0	0	Ordinal encoding of categorical variables	Imputed value: -2
2_preblipo	Categorical	0	0	Ordinal encoding of categorical variables	Imputed value: -2
2_chol	Categorical	0	0	Ordinal encoding of categorical variables	Imputed value: -2
2_TG	Categorical	0	0	Ordinal encoding of categorical variables	Imputed value: -2
2_LPA	Categorical	0	0	Ordinal encoding of categorical variables	Imputed value: -2
2_hsCRP	Categorical	0	0	Ordinal encoding of categorical variables	Imputed value: -2
2_CRP	Categorical	0	0	Ordinal encoding of categorical variables	Imputed value: -2
2_NPPB	Categorical	0	0	Ordinal encoding of categorical variables	Imputed value: -2
2_homocys	Categorical	0	0	Ordinal encoding of categorical variables	Imputed value: -2
3_blipo	Categorical	0	0	Ordinal encoding of categorical variables	Imputed value: -2
3_alipo	Categorical	0	0	Ordinal encoding of categorical variables	Imputed value: -2
3_preblipo	Categorical	0	0	Ordinal encoding of categorical variables	Imputed value: -2

3_LPA	Categorical	0	0	Ordinal encoding of categorical variables	Imputed value: -2
3_hsCRP	Categorical	0	0	Ordinal encoding of categorical variables	Imputed value: -2
3_CRP	Categorical	0	0	Ordinal encoding of categorical variables	Imputed value: -2
3_homocys	Categorical	0	0	Ordinal encoding of categorical variables	Imputed value: -2
4_LDLbetafract	Categorical	0	0	Ordinal encoding of categorical variables	Imputed value: -2
4_HDLalphafract	Categorical	0	0	Ordinal encoding of categorical variables	Imputed value: -2
4_VLDLprebetafract	Categorical	0	0	Ordinal encoding of categorical variables	Imputed value: -2
4_blipo	Categorical	0	0	Ordinal encoding of categorical variables	Imputed value: -2
4_alipo	Categorical	0	0	Ordinal encoding of categorical variables	Imputed value: -2
4_preblipo	Categorical	0	0	Ordinal encoding of categorical variables	Imputed value: -2
4_alipo2	Categorical	0	0	Ordinal encoding of categorical variables	Imputed value: -2
4_blipo2	Categorical	0	0	Ordinal encoding of categorical variables	Imputed value: -2
4_TG2	Categorical	0	0	Ordinal encoding of categorical variables	Imputed value: -2
4_CK2	Categorical	0	0	Ordinal encoding of categorical variables	Imputed value: -2
4_LPA	Categorical	0	0	Ordinal encoding of categorical variables	Imputed value: -2
4_hsCRP	Categorical	0	0	Ordinal encoding of categorical variables	Imputed value: -2
4_CRP	Categorical	0	0	Ordinal encoding of categorical variables	Imputed value: -2
4_NPPB	Categorical	0	0	Ordinal encoding of categorical variables	Imputed value: -2
4_homocys	Categorical	0	0	Ordinal encoding of categorical variables	Imputed value: -2
5_LDLbetafract	Categorical	0	0	Ordinal encoding of categorical variables	Imputed value: -2
5_HDLalphafract	Categorical	0	0	Ordinal encoding of categorical variables	Imputed value: -2
5_VLDLprebetafract	Categorical	0	0	Ordinal encoding of categorical variables	Imputed value: -2
5_blipo	Categorical	0	0	Ordinal encoding of categorical variables	Imputed value: -2
5_alipo	Categorical	0	0	Ordinal encoding of categorical variables	Imputed value: -2
5_preblipo	Categorical	0	0	Ordinal encoding of categorical variables	Imputed value: -2
5_alipo2	Categorical	0	0	Ordinal encoding of categorical variables	Imputed value: -2

5_blipo2	Categorical	0	0	Ordinal encoding of categorical variables	Imputed value: -2
5_LPA	Categorical	0	0	Ordinal encoding of categorical variables	Imputed value: -2
5_hsCRP	Categorical	0	0	Ordinal encoding of categorical variables	Imputed value: -2
5_CRP	Categorical	0	0	Ordinal encoding of categorical variables	Imputed value: -2
5_homocys	Categorical	0	0	Ordinal encoding of categorical variables	Imputed value: -2
6_LDLbetafract	Categorical	0	0	Ordinal encoding of categorical variables	Imputed value: -2
6_HDLalphafract	Categorical	0	0	Ordinal encoding of categorical variables	Imputed value: -2
6_VLDLprebetafract	Categorical	0	0	Ordinal encoding of categorical variables	Imputed value: -2
6_blipo	Categorical	0	0	Ordinal encoding of categorical variables	Imputed value: -2
6_alipo	Categorical	0	0	Ordinal encoding of categorical variables	Imputed value: -2
6_preblipo	Categorical	0	0	Ordinal encoding of categorical variables	Imputed value: -2
6_alipo2	Categorical	0	0	Ordinal encoding of categorical variables	Imputed value: -2
6_blipo2	Categorical	0	0	Ordinal encoding of categorical variables	Imputed value: -2
6_hsCRP	Categorical	0	0	Ordinal encoding of categorical variables	Imputed value: -2
6_CRP	Categorical	0	0	Ordinal encoding of categorical variables	Imputed value: -2
6_homocys	Categorical	0	0	Ordinal encoding of categorical variables	Imputed value: -2

Target: ACS-U

Data Partitioning Methodology

The modeling partitions were randomly selected using a stratified sample to preserve the distribution of the target for each partition.

Model Features and Summary Statistics

Feature Name	Var Type	Unique	Missing	Mean	Std Dev	Median	Min	Max	Target Leakage
sex	Categorical	3	2	N/A	N/A	N/A	N/A	N/A	Low
age	Numeric	68	3	55.82	13.62	56.5	13.0	88.0	Low
BMI	Numeric	117	132	27.75	5.48	26.9	18.9	57.8	Low
HbA1c_cat	Categorical	3	109	N/A	N/A	N/A	N/A	N/A	Low
HbA1c_at0	Categorical	3	296	N/A	N/A	N/A	N/A	N/A	Low
pos_FA	Numeric	2	0	0.48	0.5	0.0	0.0	1.0	Low
smoking	Numeric	2	0	0.43	0.5	0.0	0.0	1.0	Low
1_LDL	Numeric	150	109	141.604	61.85	135.5	32.0	364.0	Low
1_HDL	Numeric	63	111	51.49	15.92	49.0	16.0	99.0	Low
1_chol	Numeric	152	111	233.43	76.83	226.0	79.0	540.0	Low
1_TG2	Numeric	173	111	270.64	327.33	162.0	38.0	2418.0	Low
1_LDL2	Numeric	135	128	132.58	57.28	127.0	25.0	313.0	Low
1_HDL2	Numeric	62	131	52.208	16.48	50.5	16.0	96.0	Low
1_chol2	Numeric	183	50	218.78	72.22	212.0	76.0	452.0	Low
1_TG2	Numeric	195	52	239.51	266.606	154.0	12.0	1994.0	Low
1_CK	Numeric	114	193	211.205	326.47	146.0	20.0	3507.0	Low
1_CK2	Numeric	49	278	129.15	155.2	91.0	43.0	1183.0	Low
1_LPA	Numeric	215	52	50.507	45.19	40.1	2.4	252.0	Low
1_ApoA1	Numeric	129	143	151.14	27.91	147.4	85.9	231.0	Low
1_ApoB	Categorical	83	0	N/A	N/A	N/A	N/A	N/A	Low
1_hsCRP	Categorical	111	0	N/A	N/A	N/A	N/A	N/A	Low
1_NPPB	Numeric	120	170	134.39	181.0	70.0	5.0	1043.0	Low
1_homocys	Categorical	118	0	N/A	N/A	N/A	N/A	N/A	Low
1_vitD	Categorical	41	0	N/A	N/A	N/A	N/A	N/A	Low
2_LDL	Numeric	33	305	70.059	32.62	68.5	20.0	166.0	Low
2_HDL	Numeric	26	308	56.23	17.82	56.0	29.0	96.0	Low
2_chol	Categorical	33	0	N/A	N/A	N/A	N/A	N/A	Low
2_TG2	Categorical	34	0	N/A	N/A	N/A	N/A	N/A	Low
2_LDL2	Numeric	89	199	85.93	47.93	75.0	11.0	248.0	Low
2_HDL2	Numeric	60	204	55.049	16.79	54.0	21.0	106.0	Low

2_chol2	Numeric	99	199	159.84	55.16	145.5	57.0	340.0	Low
2_TG2	Numeric	109	197	169.46	167.41	124.0	29.0	1466.0	Low
2_CK	Numeric	62	272	166.015	118.59	128.0	38.0	679.0	Low
2_CK2	Numeric	20	318	96.095	31.81	102.0	49.0	174.0	Low
2_LPA	Numeric	46	289	65.108	49.909	59.2	2.4	237.0	Low
2_ApoA1	Numeric	24	314	167.064	35.35	158.0	105.3	236.8	Low
2_ApoB	Numeric	20	314	70.13	25.92	70.0	29.6	161.0	Low
2_hsCRP	Categorical	44	0	N/A	N/A	N/A	N/A	N/A	Low
2_NPPB	Categorical	48	0	N/A	N/A	N/A	N/A	N/A	Low
2_homocys	Categorical	39	0	N/A	N/A	N/A	N/A	N/A	Low
2_vitD	Numeric	32	289	24.85	9.89	25.0	6.0	51.0	Low

Data Quality Handling Report

**Feature Name**	**Var Type**	**Missing Count**	**Missing Percentage**	**Imputation Name**	**Imputation Description**
2_CK2	Numeric	252	93	Missing Values Imputed	Missing indicator treated as feature, Imputed value: 102
2_ApoA1	Numeric	248	92	Missing Values Imputed	Missing indicator treated as feature, Imputed value: 158
2_ApoB	Numeric	248	92	Missing Values Imputed	Imputed value: 70
2_HDL	Numeric	242	89	Missing Values Imputed	Missing indicator treated as feature, Imputed value: 56
2_LDL	Numeric	240	89	Missing Values Imputed	Missing indicator treated as feature, Imputed value: 69
HbA1c_at0	Categorical	236	87	One-Hot Encoding	Missing indicator treated as feature
2_LPA	Numeric	233	86	Missing Values Imputed	Missing indicator treated as feature, Imputed value: 53.2
2_vitD	Numeric	230	85	Missing Values Imputed	Missing indicator treated as feature, Imputed value: 25
1_CK2	Numeric	225	83	Missing Values Imputed	Missing indicator treated as feature, Imputed value: 94
2_CK	Numeric	218	80	Missing Values Imputed	Missing indicator treated as feature, Imputed value: 136
2_HDL2	Numeric	165	61	Missing Values Imputed	Missing indicator treated as feature, Imputed value: 54
2_LDL2	Numeric	162	60	Missing Values Imputed	Missing indicator treated as feature, Imputed value: 72
2_chol2	Numeric	161	59	Missing Values Imputed	Missing indicator treated as feature, Imputed value: 145

2_TG2	Numeric	158	58	Missing Values Imputed	Missing indicator treated as feature, Imputed value: 132
1_CK	Numeric	149	55	Missing Values Imputed	Missing indicator treated as feature, Imputed value: 137
1_NPPB	Numeric	139	51	Missing Values Imputed	Missing indicator treated as feature, Imputed value: 72
1_ApoA1	Numeric	113	42	Missing Values Imputed	Missing indicator treated as feature, Imputed value: 148.2
BMI	Numeric	112	41	Missing Values Imputed	Missing indicator treated as feature, Imputed value: 26.9
1_HDL2	Numeric	106	39	Missing Values Imputed	Missing indicator treated as feature, Imputed value: 51
1_LDL2	Numeric	103	38	Missing Values Imputed	Missing indicator treated as feature, Imputed value: 126
HbA1c_cat	Categorical	90	33	One-Hot Encoding	Missing indicator treated as feature
1_HDL	Numeric	86	32	Missing Values Imputed	Missing indicator treated as feature, Imputed value: 50
1_chol	Numeric	86	32	Missing Values Imputed	Imputed value: 226
1_TG2	Numeric	86	32	Missing Values Imputed	Imputed value: 165
1_LDL	Numeric	85	31	Missing Values Imputed	Missing indicator treated as feature, Imputed value: 133
1_TG2	Numeric	40	15	Missing Values Imputed	Missing indicator treated as feature, Imputed value: 160
1_chol2	Numeric	38	14	Missing Values Imputed	Missing indicator treated as feature, Imputed value: 211
1_LPA	Numeric	37	14	Missing Values Imputed	Missing indicator treated as feature, Imputed value: 38
age	Numeric	3	1	Missing Values Imputed	Missing indicator treated as feature, Imputed value: 57
sex	Categorical	1	0	One-Hot Encoding	Missing values treated as infrequent
1_ApoB	Categorical	0	0	One-Hot Encoding	Missing values treated as infrequent
1_hsCRP	Categorical	0	0	One-Hot Encoding	Missing values ignored
1_homocys	Categorical	0	0	One-Hot Encoding	Missing values ignored
1_vitD	Categorical	0	0	One-Hot Encoding	Missing values treated as infrequent
2_chol	Categorical	0	0	One-Hot Encoding	Missing values ignored
2_TG2	Categorical	0	0	One-Hot Encoding	Missing values ignored
2_hsCRP	Categorical	0	0	One-Hot Encoding	Missing values ignored
2_NPPB	Categorical	0	0	One-Hot Encoding	Missing values ignored

2_homocys	Categorical	0	0	One-Hot Encoding	Missing values ignored
pos_FA	Numeric	0	0	Missing Values Imputed	Imputed value: 0
smoking	Numeric	0	0	Missing Values Imputed	Imputed value: 0

Phase 2

Target: CAD Common

Data Partitioning Methodology

The modeling partitions were randomly selected using a stratified sample to preserve the distribution of the target for each partition.

Model Features and Summary Statistics

Feature Name	Var Type	Unique	Missing	Mean	Std Dev	Median	Min	Max	Target Leakage
weight	Numeric	453	0	79.909	14.15	80.0	41.0	145.0	Low
vldlch	Numeric	150	0	37.26	26.69	31.0	0.0	350.0	Low
vitd25	Numeric	404	15	17.44	9.84	15.5	2.5	170.1	Low
supercrp	Numeric	1039	5	8.84	17.61	3.4	0.17	199.0	Low
strokeyn	Numeric	2	0	0.09	0.29	0.0	0.0	1.0	Low
statinyn	Numeric	2	0	0.46	0.5	0.0	0.0	1.0	Low
smoclass	Numeric	3	0	0.84	0.73	1.0	0.0	2.0	Low
sex	Numeric	2	0	1.306	0.46	1.0	1.0	2.0	Low
pvdyn	Numeric	2	0	0.095	0.29	0.0	0.0	1.0	Low
pbnpl1	Numeric	1241	41	905.62	2056.24	293.0	5.0	35000.0	Low
lvangio	Numeric	4	264	1.56	0.94	1.0	1.0	4.0	Low
ldlch	Numeric	203	0	116.77	34.88	114.0	15.0	361.0	Low
insuthyn	Numeric	2	18	0.066	0.25	0.0	0.0	1.0	Low
hypten	Numeric	2	0	0.59	0.49	1.0	0.0	1.0	Low
homocys	Numeric	283	4	13.48	6.14	12.3	2.0	98.0	Low
height	Numeric	56	0	170.035	8.81	170.0	141.0	202.0	Low
hdlch	Numeric	72	0	38.63	10.77	37.0	2.0	104.0	Low
hba1c	Numeric	85	3	6.32	1.23	6.0	3.1	16.5	Low
etg	Numeric	399	0	175.86	123.54	147.0	39.0	2456.0	Low
LPA	Numeric	485	1	29.43	34.92	16.0	0.0	236.0	Low
eapob	Numeric	150	0	104.84	24.88	103.0	32.0	245.0	Low
eapoa1	Numeric	150	0	129.38	25.055	127.0	52.0	249.0	Low
dm2yn	Numeric	2	0	0.17	0.38	0.0	0.0	1.0	Low
dm1yn	Numeric	2	0	0.003	0.055	0.0	0.0	1.0	Low
crp	Numeric	416	6	9.65	19.18	4.0	0.0	234.6	Low
ck	Numeric	149	1	37.36	51.42	29.0	4.0	1698.0	Low
chol	Numeric	240	0	209.058	44.27	205.0	92.0	453.0	Low

carosten	Numeric	2	4	0.049	0.22	0.0	0.0	1.0	Low
cadyn	Numeric	2	0	0.78	0.41	1.0	0.0	1.0	N/A
bmi	Numeric	1679	0	27.58	4.098	27.17	16.34	48.34	Low
age	Numeric	2401	0	62.59	10.73	63.49	18.55	92.101	Low
afibyn	Numeric	2	24	0.12	0.33	0.0	0.0	1.0	Low
COPDyn	Numeric	2	0	0.0305	0.17	0.0	0.0	1.0	Low

Data Quality Handling Report

Feature Name	Var Type	Missing Count	Missing Percentage	Imputation Name	Imputation Description
lvangio	Numeric	334	10	Missing Values Imputed	Missing indicator treated as feature, Imputed value: 1
pbnpl1	Numeric	51	2	Missing Values Imputed	Missing indicator treated as feature, Imputed value: 293
afibyn	Numeric	32	1	Missing Values Imputed	Missing indicator treated as feature, Imputed value: 0
insuthyn	Numeric	18	1	Missing Values Imputed	Missing indicator treated as feature, Imputed value: 0
vitd25	Numeric	17	1	Missing Values Imputed	Missing indicator treated as feature, Imputed value: 15.6
supercrp	Numeric	6	0	Missing Values Imputed	Missing indicator treated as feature, Imputed value: 3.39
crp	Numeric	6	0	Missing Values Imputed	Missing indicator treated as feature, Imputed value: 4.1
homocys	Numeric	4	0	Missing Values Imputed	Missing indicator treated as feature, Imputed value: 12.4
hba1c	Numeric	4	0	Missing Values Imputed	Missing indicator treated as feature, Imputed value: 6
carosten	Numeric	4	0	Missing Values Imputed	Missing indicator treated as feature, Imputed value: 0
LPA	Numeric	3	0	Missing Values Imputed	Missing indicator treated as feature, Imputed value: 16
vldlch	Numeric	1	0	Missing Values Imputed	Missing indicator treated as feature, Imputed value: 31
ldlch	Numeric	1	0	Missing Values Imputed	Imputed value: 114
hdlch	Numeric	1	0	Missing Values Imputed	Imputed value: 37
etg	Numeric	1	0	Missing Values Imputed	Imputed value: 147
eapob	Numeric	1	0	Missing Values Imputed	Imputed value: 103
eapoa1	Numeric	1	0	Missing Values Imputed	Imputed value: 127
ck	Numeric	1	0	Missing Values Imputed	Missing indicator treated as feature, Imputed value: 29

weight	Numeric	0	0	Missing Values Imputed	Imputed value: 79.9
strokeyn	Numeric	0	0	Missing Values Imputed	Imputed value: 0
statinyn	Numeric	0	0	Missing Values Imputed	Imputed value: 0
smoclass	Numeric	0	0	Missing Values Imputed	Imputed value: 1
sex	Numeric	0	0	Missing Values Imputed	Imputed value: 1
pvdyn	Numeric	0	0	Missing Values Imputed	Imputed value: 0
hypten	Numeric	0	0	Missing Values Imputed	Imputed value: 1
height	Numeric	0	0	Missing Values Imputed	Imputed value: 170
dm2yn	Numeric	0	0	Missing Values Imputed	Imputed value: 0
dm1yn	Numeric	0	0	Missing Values Imputed	Imputed value: 0
chol	Numeric	0	0	Missing Values Imputed	Imputed value: 205
bmi	Numeric	0	0	Missing Values Imputed	Imputed value: 27.0602
age	Numeric	0	0	Missing Values Imputed	Imputed value: 63.5418
COPDyn	Numeric	0	0	Missing Values Imputed	Imputed value: 0

Target: MI Common

Data Partitioning Methodology

The modeling partitions were randomly selected using a stratified sample to preserve the distribution of the target for each partition.

Model Features and Summary Statistics

Feature Name	Var Type	Unique	Missing	Mean	Std Dev	Median	Min	Max	Target Leakage
weight	Numeric	447	0	79.76	14.24	79.6	41.0	185.0	Low
vldlch	Numeric	150	1	36.95	26.39	31.0	0.0	350.0	Low
vitd25	Numeric	406	13	17.44	9.77	15.7	1.9	170.1	Low
supercrp	Numeric	1051	4	8.801	17.502	3.33	0.17	199.0	Low
strokeyn	Numeric	2	0	0.0901	0.29	0.0	0.0	1.0	Low
statinyn	Numeric	2	0	0.47	0.5	0.0	0.0	1.0	Low
smoclass	Numeric	3	0	0.85	0.73	1.0	0.0	2.0	Low
sex	Numeric	2	0	1.307	0.46	1.0	1.0	2.0	Low
pvdyn	Numeric	2	0	0.094	0.29	0.0	0.0	1.0	Low
pbnpl1	Numeric	1220	41	892.33	1924.17	291.0	5.0	35000.0	Low
miyn	Numeric	2	0	0.41	0.49	0.0	0.0	1.0	N/A
lvangio	Numeric	4	274	1.54	0.94	1.0	1.0	4.0	Low
ldlch	Numeric	200	1	116.83	34.51	114.0	15.0	361.0	Low
insuthyn	Numeric	2	16	0.065	0.25	0.0	0.0	1.0	Low
hypten	Numeric	2	0	0.58	0.49	1.0	0.0	1.0	Low
homocys	Numeric	285	4	13.45	6.068	12.3	2.0	98.0	Low
height	Numeric	54	0	170.038	8.77	170.0	141.0	202.0	Low
hdlch	Numeric	73	1	38.79	10.79	37.0	2.0	91.0	Low
hba1c	Numeric	87	3	6.33	1.26	6.0	3.1	18.6	Low
etg	Numeric	405	1	173.66	117.86	147.0	39.0	2456.0	Low
LPA	Numeric	477	3	29.13	34.71	16.0	0.0	236.0	Low
eapob	Numeric	148	1	104.65	24.45	103.0	32.0	229.0	Low
eapoa1	Numeric	151	1	129.57	25.057	127.0	52.0	249.0	Low
dm2yn	Numeric	2	0	0.18	0.38	0.0	0.0	1.0	Low
dm1yn	Numeric	2	0	0.0026	0.051	0.0	0.0	1.0	Low
crp	Numeric	420	5	9.71	19.16	4.1	0.1	234.6	Low
ck	Numeric	152	1	37.306	51.039	29.0	2.0	1698.0	Low
chol	Numeric	240	0	208.92	44.12	205.0	79.0	453.0	Low
carosten	Numeric	2	3	0.049	0.22	0.0	0.0	1.0	Low
bmi	Numeric	1656	0	27.52	4.108	27.054	16.35	57.099	Low

age	Numeric	2398	0	62.53	10.64	63.37	17.25	92.101	Low
afibyn	Numeric	2	28	0.12	0.33	0.0	0.0	1.0	Low
COPDyn	Numeric	2	0	0.0302	0.17	0.0	0.0	1.0	Low

Data Quality Handling Report

Feature Name	Var Type	Missing Count	Missing Percentage	Imputation Name	Imputation Description
lvangio	Numeric	334	10	Missing Values Imputed	Missing indicator treated as feature, Imputed value: 1
pbnpl1	Numeric	51	2	Missing Values Imputed	Missing indicator treated as feature, Imputed value: 293
afibyn	Numeric	32	1	Missing Values Imputed	Missing indicator treated as feature, Imputed value: 0
insuthyn	Numeric	18	1	Missing Values Imputed	Missing indicator treated as feature, Imputed value: 0
vitd25	Numeric	17	1	Missing Values Imputed	Missing indicator treated as feature, Imputed value: 15.6
supercrp	Numeric	6	0	Missing Values Imputed	Missing indicator treated as feature, Imputed value: 3.39
crp	Numeric	6	0	Missing Values Imputed	Missing indicator treated as feature, Imputed value: 4.1
homocys	Numeric	4	0	Missing Values Imputed	Missing indicator treated as feature, Imputed value: 12.4
hba1c	Numeric	4	0	Missing Values Imputed	Missing indicator treated as feature, Imputed value: 6
carosten	Numeric	4	0	Missing Values Imputed	Missing indicator treated as feature, Imputed value: 0
LPA	Numeric	3	0	Missing Values Imputed	Missing indicator treated as feature, Imputed value: 16
vldlch	Numeric	1	0	Missing Values Imputed	Missing indicator treated as feature, Imputed value: 31
ldlch	Numeric	1	0	Missing Values Imputed	Imputed value: 114
hdlch	Numeric	1	0	Missing Values Imputed	Imputed value: 37
etg	Numeric	1	0	Missing Values Imputed	Imputed value: 147
eapob	Numeric	1	0	Missing Values Imputed	Imputed value: 103
eapoa1	Numeric	1	0	Missing Values Imputed	Imputed value: 127
ck	Numeric	1	0	Missing Values Imputed	Missing indicator treated as feature, Imputed value: 29
weight	Numeric	0	0	Missing Values Imputed	Imputed value: 79.9
strokeyn	Numeric	0	0	Missing Values Imputed	Imputed value: 0
statinyn	Numeric	0	0	Missing Values Imputed	Imputed value: 0

smoclass	Numeric	0	0	Missing Values Imputed	Imputed value: 1
sex	Numeric	0	0	Missing Values Imputed	Imputed value: 1
pvdyn	Numeric	0	0	Missing Values Imputed	Imputed value: 0
hypten	Numeric	0	0	Missing Values Imputed	Imputed value: 1
height	Numeric	0	0	Missing Values Imputed	Imputed value: 170
dm2yn	Numeric	0	0	Missing Values Imputed	Imputed value: 0
dm1yn	Numeric	0	0	Missing Values Imputed	Imputed value: 0
chol	Numeric	0	0	Missing Values Imputed	Imputed value: 205
bmi	Numeric	0	0	Missing Values Imputed	Imputed value: 27.0602
age	Numeric	0	0	Missing Values Imputed	Imputed value: 63.5418
COPDyn	Numeric	0	0	Missing Values Imputed	Imputed value: 0

Target: Stroke Common

Data Partitioning Methodology

The modeling partitions were randomly selected using a stratified sample to preserve the distribution of the target for each partition.

Model Features and Summary Statistics

Feature Name	Var Type	Unique	Missing	Mean	Std Dev	Median	Min	Max	Target Leakage
weight	Numeric	454	0	79.79	14.066	80.0	41.0	185.0	Low
vitd25	Numeric	408	16	17.56	9.43	15.9	1.9	75.6	Low
strokeyn	Numeric	2	0	0.092	0.29	0.0	0.0	1.0	N/A
statinyn	Numeric	2	0	0.46	0.5	0.0	0.0	1.0	Low
sex	Numeric	2	0	1.3	0.46	1.0	1.0	2.0	Low
miyn	Numeric	2	0	0.42	0.49	0.0	0.0	1.0	Low
lvangio	Numeric	4	262	1.54	0.94	1.0	1.0	4.0	Low
hypten	Numeric	2	0	0.59	0.49	1.0	0.0	1.0	Low
homocys	Numeric	279	4	13.39	5.98	12.3	2.0	98.0	Low
hba1c	Numeric	86	3	6.3009	1.25	6.0	3.9	18.6	Low
eapoa1	Numeric	147	1	129.46	24.72	127.0	52.0	249.0	Low
dm2yn	Numeric	2	0	0.17	0.38	0.0	0.0	1.0	Low
ck	Numeric	152	0	36.84	39.59	29.0	2.0	1090.0	Low
carosten	Numeric	2	4	0.0502	0.22	0.0	0.0	1.0	Low
age	Numeric	2404	0	62.48	10.75	63.42	17.25	92.101	Low
afibyn	Numeric	2	21	0.12	0.33	0.0	0.0	1.0	Low

Data Quality Handling Report

Feature Name	Var Type	Missing Count	Missing Percentage	Imputation Name	Imputation Description
lvangio	Numeric	334	10	Missing Values Imputed	Missing indicator treated as feature, Imputed value: 1
afibyn	Numeric	32	1	Missing Values Imputed	Missing indicator treated as feature, Imputed value: 0
vitd25	Numeric	17	1	Missing Values Imputed	Missing indicator treated as feature, Imputed value: 15.6
homocys	Numeric	4	0	Missing Values Imputed	Missing indicator treated as feature, Imputed value: 12.4
hba1c	Numeric	4	0	Missing Values Imputed	Missing indicator treated as feature, Imputed value: 6
carosten	Numeric	4	0	Missing Values Imputed	Missing indicator treated as feature, Imputed value: 0

eapoa1	Numeric	1	0	Missing Values Imputed	Missing indicator treated as feature, Imputed value: 127
ck	Numeric	1	0	Missing Values Imputed	Missing indicator treated as feature, Imputed value: 29
weight	Numeric	0	0	Missing Values Imputed	Imputed value: 79.9
statinyn	Numeric	0	0	Missing Values Imputed	Imputed value: 0
sex	Numeric	0	0	Missing Values Imputed	Imputed value: 1
miyn	Numeric	0	0	Missing Values Imputed	Imputed value: 0
hypten	Numeric	0	0	Missing Values Imputed	Imputed value: 1
dm2yn	Numeric	0	0	Missing Values Imputed	Imputed value: 0
age	Numeric	0	0	Missing Values Imputed	Imputed value: 63.5418

Target: PAD Common

Data Partitioning Methodology

The modeling partitions were randomly selected using a stratified sample to preserve the distribution of the target for each partition.

Model Features and Summary Statistics

Feature Name	Var Type	Unique	Missing	Mean	Std Dev	Median	Min	Max	Target Leakage
weight	Numeric	455	0	79.62	13.97	79.5	41.0	185.0	Low
vldlch	Numeric	149	1	37.021	26.48	31.0	0.0	350.0	Low
vitd25	Numeric	402	15	17.46	9.81	15.6	1.9	170.1	Low
supercrp	Numeric	1047	6	9.17	18.53	3.42	0.17	269.0	Low
strokeyn	Numeric	2	0	0.093	0.29	0.0	0.0	1.0	Low
statinyn	Numeric	2	0	0.47	0.5	0.0	0.0	1.0	Low
smoclass	Numeric	3	0	0.84	0.73	1.0	0.0	2.0	Low
sex	Numeric	2	0	1.306	0.46	1.0	1.0	2.0	Low
pvdyn	Numeric	2	0	0.096	0.29	0.0	0.0	1.0	N/A
pbnpl1	Numeric	1240	39	924.2	2082.0032	296.0	5.0	35000.0	Low
miyn	Numeric	2	0	0.42	0.49	0.0	0.0	1.0	Low
lvangio	Numeric	4	271	1.55	0.94	1.0	1.0	4.0	Low
ldlch	Numeric	196	1	116.24	33.86	114.0	15.0	329.0	Low
insuthyn	Numeric	2	16	0.063	0.24	0.0	0.0	1.0	Low
hypten	Numeric	2	0	0.59	0.49	1.0	0.0	1.0	Low
homocys	Numeric	282	3	13.48	6.15	12.3	3.3	98.0	Low
height	Numeric	56	0	170.16	8.66	170.0	141.0	202.0	Low
hdlch	Numeric	73	1	38.77	10.89	37.0	2.0	104.0	Low
hba1c	Numeric	84	3	6.3	1.24	6.0	3.1	18.6	Low
etg	Numeric	398	1	173.49	117.034	147.0	39.0	2456.0	Low
LPA	Numeric	474	2	29.0408	34.54	16.0	0.0	236.0	Low
earlycad	Numeric	2	588	0.21	0.409	0.0	0.0	1.0	Low
eapob	Numeric	143	1	104.29	24.405	103.0	32.0	225.0	Low
eapoa1	Numeric	147	1	129.53	25.14	126.0	62.0	249.0	Low
dm2yn	Numeric	2	0	0.18	0.38	0.0	0.0	1.0	Low
dm1yn	Numeric	2	0	0.0026	0.051	0.0	0.0	1.0	Low
crp	Numeric	423	5	10.0	19.88	4.1	0.0	282.4	Low
ck	Numeric	149	1	37.45	50.69	29.0	2.0	1698.0	Low
chol	Numeric	233	0	208.23	43.38	205.0	79.0	446.0	Low
carosten	Numeric	2	2	0.046	0.21	0.0	0.0	1.0	Low

cadyn	Numeric	2	0	0.78	0.42	1.0	0.0	1.0	Low
bmi	Numeric	1671	0	27.44	4.059	27.084	16.34	57.099	Low
age	Numeric	2413	0	62.55	10.7	63.44	17.25	92.101	Low
afibyn	Numeric	2	21	0.12	0.33	0.0	0.0	1.0	Low
acsyn	Numeric	2	0	0.32	0.46	0.0	0.0	1.0	Low
COPDyn	Numeric	2	0	0.0309	0.17	0.0	0.0	1.0	Low

Data Quality Handling Report

Feature Name	Var Type	Missing Count	Missing Percentage	Imputation Name	Imputation Description
earlycad	Numeric	461	22	Missing Values Imputed	Imputed value: -9999
lvangio	Numeric	218	10	Missing Values Imputed	Imputed value: -9999
pbnpl1	Numeric	32	2	Missing Values Imputed	Imputed value: -9999
afibyn	Numeric	17	1	Missing Values Imputed	Imputed value: -9999
vitd25	Numeric	12	1	Missing Values Imputed	Imputed value: -9999
insuthyn	Numeric	12	1	Missing Values Imputed	Imputed value: -9999
crp	Numeric	5	0	Missing Values Imputed	Imputed value: -9999
supercrp	Numeric	4	0	Missing Values Imputed	Missing indicator treated as feature, Imputed value: 3.46
hba1c	Numeric	3	0	Missing Values Imputed	Missing indicator treated as feature, Imputed value: 6
homocys	Numeric	2	0	Missing Values Imputed	Missing indicator treated as feature, Imputed value: 12.3
carosten	Numeric	2	0	Missing Values Imputed	Missing indicator treated as feature, Imputed value: 0
vldlch	Numeric	1	0	Missing Values Imputed	Missing indicator treated as feature, Imputed value: 31
ldlch	Numeric	1	0	Missing Values Imputed	Imputed value: 114
hdlch	Numeric	1	0	Missing Values Imputed	Imputed value: 37
etg	Numeric	1	0	Missing Values Imputed	Imputed value: 146
LPA	Numeric	1	0	Missing Values Imputed	Imputed value: 16
eapob	Numeric	1	0	Missing Values Imputed	Imputed value: 102
eapoa1	Numeric	1	0	Missing Values Imputed	Imputed value: 126
ck	Numeric	1	0	Missing Values Imputed	Missing indicator treated as feature, Imputed value: 29
weight	Numeric	0	0	Missing Values Imputed	Imputed value: 80
strokeyn	Numeric	0	0	Missing Values Imputed	Imputed value: 0
statinyn	Numeric	0	0	Missing Values Imputed	Imputed value: 0
smoclass	Numeric	0	0	Missing Values Imputed	Imputed value: 1
sex	Numeric	0	0	Missing Values Imputed	Imputed value: 1

miyn	Numeric	0	0	Missing Values Imputed	Imputed value: 0
hypten	Numeric	0	0	Missing Values Imputed	Imputed value: 1
height	Numeric	0	0	Missing Values Imputed	Imputed value: 170
dm2yn	Numeric	0	0	Missing Values Imputed	Imputed value: 0
dm1yn	Numeric	0	0	Missing Values Imputed	Imputed value: 0
chol	Numeric	0	0	Missing Values Imputed	Imputed value: 204
cadyn	Numeric	0	0	Missing Values Imputed	Imputed value: 1
bmi	Numeric	0	0	Missing Values Imputed	Imputed value: 27.1203
age	Numeric	0	0	Missing Values Imputed	Imputed value: 63.4186
acsyn	Numeric	0	0	Missing Values Imputed	Imputed value: 0
COPDyn	Numeric	0	0	Missing Values Imputed	Imputed value: 0

Target: ACS Common

Data Partitioning Methodology

Data partitions were selected by means of random sampling.

Model Features and Summary Statistics

Feature Name	Var Type	Unique	Missing	Mean	Std Dev	Median	Min	Max	Target Leakage
weight	Numeric	450	0	79.87	14.0083	80.0	41.0	143.0	Low
vldlch	Numeric	146	1	36.52	25.35	31.0	0.0	350.0	Low
vitd25	Numeric	399	14	17.24	9.24	15.5	1.9	75.6	Low
supercrp	Numeric	1049	4	9.17	18.84	3.34	0.17	269.0	Low
strokeyn	Numeric	2	0	0.092	0.29	0.0	0.0	1.0	Low
statinyn	Numeric	2	0	0.47	0.5	0.0	0.0	1.0	Low
smoclass	Numeric	3	0	0.84	0.73	1.0	0.0	2.0	Low
sex	Numeric	2	0	1.301	0.46	1.0	1.0	2.0	Low
pvdyn	Numeric	2	0	0.093	0.29	0.0	0.0	1.0	Low
pbnpl1	Numeric	1242	33	930.77	2179.35	294.0	5.0	35000.0	Low
lvangio	Numeric	4	284	1.56	0.95	1.0	1.0	4.0	Low
ldlch	Numeric	198	1	116.204	34.37	113.0	15.0	361.0	Low
insuthyn	Numeric	2	13	0.064	0.24	0.0	0.0	1.0	Low
hypten	Numeric	2	0	0.59	0.49	1.0	0.0	1.0	Low
homocys	Numeric	285	2	13.52	6.15	12.3	3.3	98.0	Low
height	Numeric	54	0	170.25	8.65	170.0	141.0	202.0	Low
hdlch	Numeric	72	1	38.84	10.77	37.0	11.0	104.0	Low
hba1c	Numeric	85	3	6.32	1.25	6.0	3.9	18.6	Low
etg	Numeric	392	1	171.33	113.66	146.0	39.0	2456.0	Low
LPA	Numeric	478	2	28.67	34.093	16.0	0.0	236.0	Low
eapob	Numeric	148	1	104.014	24.93	102.0	32.0	245.0	Low
eapoa1	Numeric	149	1	129.53	24.97	127.0	52.0	241.0	Low
dm2yn	Numeric	2	0	0.18	0.38	0.0	0.0	1.0	Low
dm1yn	Numeric	2	0	0.0026	0.051	0.0	0.0	1.0	Low
crp	Numeric	425	5	9.97	20.36	4.0	0.1	282.4	Low
ck	Numeric	147	1	37.18	50.65	29.0	2.0	1698.0	Low
chol	Numeric	242	0	207.85	44.11	205.0	79.0	453.0	Low
carosten	Numeric	2	3	0.0502	0.22	0.0	0.0	1.0	Low
bmi	Numeric	1660	0	27.5	4.072	27.055	16.35	48.34	Low
age	Numeric	2417	0	62.63	10.62	63.58	17.25	92.101	Low
afibyn	Numeric	2	26	0.12	0.33	0.0	0.0	1.0	Low

acsyn	Numeric	2	0	0.31	0.46	0.0	0.0	1.0	N/A
COPDyn	Numeric	2	0	0.0309	0.17	0.0	0.0	1.0	Low

Data Quality Handling Report

Feature Name	Var Type	Missing Count	Missing Percentage	Imputation Name	Imputation Description
lvangio	Numeric	226	11	Missing Values Imputed	Imputed value: -9999
pbnpl1	Numeric	26	1	Missing Values Imputed	Imputed value: -9999
afibyn	Numeric	19	1	Missing Values Imputed	Imputed value: -9999
insuthyn	Numeric	12	1	Missing Values Imputed	Imputed value: -9999
vitd25	Numeric	10	0	Missing Values Imputed	Imputed value: -9999
supercrp	Numeric	4	0	Missing Values Imputed	Missing indicator treated as feature, Imputed value: 3.42
crp	Numeric	4	0	Missing Values Imputed	Missing indicator treated as feature, Imputed value: 4.1
carosten	Numeric	3	0	Missing Values Imputed	Missing indicator treated as feature, Imputed value: 0
homocys	Numeric	2	0	Missing Values Imputed	Missing indicator treated as feature, Imputed value: 12.4
hba1c	Numeric	2	0	Missing Values Imputed	Missing indicator treated as feature, Imputed value: 6
LPA	Numeric	2	0	Missing Values Imputed	Missing indicator treated as feature, Imputed value: 16
vldlch	Numeric	1	0	Missing Values Imputed	Missing indicator treated as feature, Imputed value: 31
ldlch	Numeric	1	0	Missing Values Imputed	Imputed value: 114
hdlch	Numeric	1	0	Missing Values Imputed	Imputed value: 37
etg	Numeric	1	0	Missing Values Imputed	Imputed value: 146
eapob	Numeric	1	0	Missing Values Imputed	Imputed value: 102
eapoa1	Numeric	1	0	Missing Values Imputed	Imputed value: 127
weight	Numeric	0	0	Missing Values Imputed	Imputed value: 80
strokeyn	Numeric	0	0	Missing Values Imputed	Imputed value: 0
statinyn	Numeric	0	0	Missing Values Imputed	Imputed value: 0
smoclass	Numeric	0	0	Missing Values Imputed	Imputed value: 1
sex	Numeric	0	0	Missing Values Imputed	Imputed value: 1
pvdyn	Numeric	0	0	Missing Values Imputed	Imputed value: 0
hypten	Numeric	0	0	Missing Values Imputed	Imputed value: 1
height	Numeric	0	0	Missing Values Imputed	Imputed value: 170
dm2yn	Numeric	0	0	Missing Values Imputed	Imputed value: 0
dm1yn	Numeric	0	0	Missing Values Imputed	Imputed value: 0

ck	Numeric	0	0	Missing Values Imputed	Imputed value: 29
chol	Numeric	0	0	Missing Values Imputed	Imputed value: 205
bmi	Numeric	0	0	Missing Values Imputed	Imputed value: 27.1178
age	Numeric	0	0	Missing Values Imputed	Imputed value: 63.4925
COPDyn	Numeric	0	0	Missing Values Imputed	Imputed value: 0

Phase 3

Target: EoL-1

Data Partitioning Methodology

The modeling partitions were randomly selected using a stratified sample to preserve the distribution of the target for each partition.

Model Features and Summary Statistics

Feature Name	Var Type	Unique	Missing	Mean	Std Dev	Median	Min	Max	Target Leakage
weight	Numeric	432	0	79.909	13.99	80.0	41.0	185.0	Low
vitd25	Numeric	399	7	17.84	9.82	16.2	1.9	170.1	Low
supercrp	Numeric	977	6	8.808	18.41	3.2	0.17	269.0	Low
strokeyn	Numeric	2	0	0.083	0.28	0.0	0.0	1.0	Low
statinyn	Numeric	2	0	0.46	0.5	0.0	0.0	1.0	Low
smoclass	Numeric	3	0	0.82	0.73	1.0	0.0	2.0	Low
sex	Numeric	2	0	1.31	0.46	1.0	1.0	2.0	Low
pvdyn	Numeric	2	0	0.077	0.27	0.0	0.0	1.0	Low
pbnpl1	Numeric	1090	38	869.39	2205.66	263.0	5.0	35000.0	Low
miyn	Numeric	2	0	0.4008	0.49	0.0	0.0	1.0	Low
lvangio	Numeric	4	239	1.5	0.9	1.0	1.0	4.0	Low
ldlch	Numeric	196	1	116.84	34.19	115.0	15.0	361.0	Low
insuthyn	Numeric	2	15	0.057	0.23	0.0	0.0	1.0	Low
homocys	Numeric	264	3	13.204	5.84	12.1	2.0	98.0	Low
hdlch	Numeric	74	1	39.071	11.0803	37.5	2.0	104.0	Low
earlycad	Numeric	2	553	0.22	0.41	0.0	0.0	1.0	Low
eapob	Numeric	143	1	104.31	24.69	103.0	32.0	245.0	Low
dm2yn	Numeric	2	0	0.16	0.37	0.0	0.0	1.0	Low
crp	Numeric	388	2	9.6008	19.77	3.8	0.0	282.4	Low
carosten	Numeric	2	1	0.045	0.207	0.0	0.0	1.0	Low
cadyn	Numeric	2	0	0.77	0.42	1.0	0.0	1.0	Low
bmi	Numeric	1524	0	27.52	4.0085	27.14	16.34	57.099	Low
age	Numeric	2165	0	61.86	10.59	62.71	17.25	92.101	Low
afibyn	Numeric	2	19	0.12	0.32	0.0	0.0	1.0	Low
acsyn	Numeric	2	0	0.306	0.46	0.0	0.0	1.0	Low
death2010_c3	Numeric	2	0	0.21	0.408	0.0	0.0	1.0	N/A

Data Quality Handling Report

Feature Name	Var Type	Missing Count	Missing Percentage	Imputation Name	Imputation Description
earlycad (Categorical Int)	Categorical	682	23	One-Hot Encoding	Missing indicator treated as feature
lvangio (Categorical Int)	Categorical	302	10	One-Hot Encoding	Missing indicator treated as feature
pbnpl1	Numeric	46	2	Missing Values Imputed	Missing indicator treated as feature, Imputed value: 271
afibyn (Categorical Int)	Categorical	27	1	One-Hot Encoding	Missing indicator treated as feature
insuthyn (Categorical Int)	Categorical	18	1	One-Hot Encoding	Missing indicator treated as feature
vitd25	Numeric	13	0	Missing Values Imputed	Missing indicator treated as feature, Imputed value: 16.1
supercrp	Numeric	6	0	Missing Values Imputed	Missing indicator treated as feature, Imputed value: 3.27
crp	Numeric	6	0	Missing Values Imputed	Missing indicator treated as feature, Imputed value: 3.9
carosten (Categorical Int)	Categorical	4	0	One-Hot Encoding	Missing values treated as infrequent
homocys	Numeric	3	0	Missing Values Imputed	Missing indicator treated as feature, Imputed value: 12.2
ldlch	Numeric	1	0	Missing Values Imputed	Missing indicator treated as feature, Imputed value: 115
hdlch	Numeric	1	0	Missing Values Imputed	Imputed value: 37
eapob	Numeric	1	0	Missing Values Imputed	Imputed value: 103
strokeyn (Categorical Int)	Categorical	0	0	One-Hot Encoding	Missing values ignored
pvdyn (Categorical Int)	Categorical	0	0	One-Hot Encoding	Missing values ignored
smoclass (Categorical Int)	Categorical	0	0	One-Hot Encoding	Missing values ignored
statinyn (Categorical Int)	Categorical	0	0	One-Hot Encoding	Missing values ignored
sex (Categorical Int)	Categorical	0	0	One-Hot Encoding	Missing values ignored
miyn (Categorical Int)	Categorical	0	0	One-Hot Encoding	Missing values ignored
dm2yn (Categorical Int)	Categorical	0	0	One-Hot Encoding	Missing values ignored
acsyn (Categorical Int)	Categorical	0	0	One-Hot Encoding	Missing values ignored
cadyn (Categorical Int)	Categorical	0	0	One-Hot Encoding	Missing values ignored
weight	Numeric	0	0	Missing Values Imputed	Imputed value: 80
bmi	Numeric	0	0	Missing Values Imputed	Imputed value: 27.1416
age	Numeric	0	0	Missing Values Imputed	Imputed value: 62.8135

Target: EoL-2

Data Partitioning Methodology

Data partitions were selected by means of random sampling.

Model Features and Summary Statistics

Feature Name	Var Type	Unique	Missing	Mean	Std Dev	Median	Min	Max	Target Leakage
sex	Numeric	2	0	1.3	0.46	1.0	1.0	2.0	Low
hyptenyn	Numeric	2	0	0.59	0.49	1.0	0.0	1.0	Low
chol	Numeric	233	0	208.92	44.28	207.0	79.0	453.0	Low
hdlch	Numeric	73	1	38.85	11.016	37.0	2.0	104.0	Low
age	Numeric	2176	0	62.051	10.62	62.83	17.25	92.101	Low
smoclass	Numeric	3	0	0.83	0.73	1.0	0.0	2.0	Low

Data Quality Handling Report

Feature Name	Var Type	Missing Count	Missing Percentage	Imputation Name	Imputation Description
hdlch	Numeric	1	0	Missing Values Imputed	Missing indicator treated as feature, Imputed value: 37
smoclass (Categorical Int)	Categorical	0	0	One-Hot Encoding	Missing values ignored
sex (Categorical Int)	Categorical	0	0	One-Hot Encoding	Missing values ignored
hyptenyn (Categorical Int)	Categorical	0	0	One-Hot Encoding	Missing values ignored
chol	Numeric	0	0	Missing Values Imputed	Imputed value: 206
age	Numeric	0	0	Missing Values Imputed	Imputed value: 62.8135

Target: EoL-3

Data Partitioning Methodology

The modeling partitions were randomly selected using a stratified sample to preserve the distribution of the target for each partition.

Model Features and Summary Statistics

Feature Name	Var Type	Unique	Missing	Mean	Std Dev	Median	Min	Max	Target Leakage
sex	Numeric	2	0	1.31	0.46	1.0	1.0	2.0	Low
urea	Numeric	96	2	38.95	15.25	36.0	7.0	209.0	Low
uricacid	Numeric	110	1	5.082	1.7	4.8	0.9	17.8	Low
ferritin	Numeric	584	0	216.28	204.0094	157.0	5.0	2232.0	Low
hba1c	Numeric	83	3	6.28	1.21	6.0	3.9	18.6	Low
vitd25	Numeric	399	7	17.84	9.82	16.2	1.9	170.1	Low
vitd125	Numeric	581	0	35.43	14.0094	33.6	3.3	123.0	Low
fii	Numeric	151	462	104.66	25.72	107.0	9.0	199.0	Low
vwfag	Numeric	287	7	165.69	69.48	154.0	21.0	502.0	Low
ddimer	Numeric	256	27	0.602	1.12	0.34	0.0	22.0	Low
tpaant	Numeric	298	2	13.19	6.84	11.7	1.3	89.6	Low
elpa	Numeric	440	3	29.77	35.078	16.0	0.0	236.0	Low
cystatc	Numeric	170	6	0.98	0.38	0.9	0.35	7.44	Low
age	Numeric	2165	0	61.86	10.59	62.71	17.25	92.101	Low
smoclass	Numeric	3	0	0.82	0.73	1.0	0.0	2.0	Low
pbnpl1	Numeric	1090	38	869.39	2205.66	263.0	5.0	35000.0	Low
death2010_c3	Numeric	2	0	0.21	0.408	0.0	0.0	1.0	N/A
TnThs	Numeric	746	69	101.88	439.5	10.0	1.5	6640.0	Low

Data Quality Handling Report

Feature Name	Var Type	Missing Count	Missing Percentage	Imputation Name	Imputation Description
fii	Numeric	576	20	Missing Values Imputed	Missing indicator treated as feature, Imputed value: 107
TnThs	Numeric	86	3	Missing Values Imputed	Missing indicator treated as feature, Imputed value: 10
pbnpl1	Numeric	46	2	Missing Values Imputed	Missing indicator treated as feature, Imputed value: 271
ddimer	Numeric	33	1	Missing Values Imputed	Missing indicator treated as feature, Imputed value: 0.35
vitd25	Numeric	13	0	Missing Values Imputed	Missing indicator treated as feature, Imputed value: 16.1

vwfag	Numeric	11	0	Missing Values Imputed	Missing indicator treated as feature, Imputed value: 154
cystatc	Numeric	6	0	Missing Values Imputed	Missing indicator treated as feature, Imputed value: 0.9
hba1c	Numeric	3	0	Missing Values Imputed	Missing indicator treated as feature, Imputed value: 6
tpaant	Numeric	3	0	Missing Values Imputed	Missing indicator treated as feature, Imputed value: 11.7
elpa	Numeric	3	0	Missing Values Imputed	Missing indicator treated as feature, Imputed value: 16
urea	Numeric	2	0	Missing Values Imputed	Missing indicator treated as feature, Imputed value: 36
uricacid	Numeric	1	0	Missing Values Imputed	Missing indicator treated as feature, Imputed value: 4.8
smoclass (Categorical Int)	Categorical	0	0	Ordinal encoding of categorical variables	Imputed value: -2
sex (Categorical Int)	Categorical	0	0	Ordinal encoding of categorical variables	Imputed value: -2
ferritin	Numeric	0	0	Missing Values Imputed	Imputed value: 158
vitd125	Numeric	0	0	Missing Values Imputed	Imputed value: 33.4
age	Numeric	0	0	Missing Values Imputed	Imputed value: 62.8135

Target: EoL-4

Data Partitioning Methodology

Data partitions were selected by means of random sampling.

Model Features and Summary Statistics

Feature Name	Var Type	Unique	Missing	Mean	Std Dev	Median	Min	Max	Target Leakage
sex	Numeric	2	0	1.3	0.46	1.0	1.0	2.0	Low
hba1c	Numeric	85	2	6.29	1.22	6.0	3.9	16.5	Low
vitd25	Numeric	396	12	17.903	9.87	16.3	1.9	170.1	Low
vitd125	Numeric	583	0	35.42	13.87	33.5	3.3	113.0	Low
crp	Numeric	400	6	9.67	19.29	3.9	0.0	282.4	Low
elpa	Numeric	450	3	30.11	35.42	16.0	0.0	236.0	Low
cystatc	Numeric	173	5	0.99	0.39	0.9	0.35	7.44	Low
age	Numeric	2176	0	62.051	10.62	62.83	17.25	92.101	Low
smoclass	Numeric	3	0	0.83	0.73	1.0	0.0	2.0	Low
pbnpl1	Numeric	1110	30	889.89	2193.96	281.0	5.0	35000.0	Low
TnThs	Numeric	793	66	111.207	464.16	10.0	1.5	6640.0	Low
Galectin3	Numeric	279	524	15.49	6.97	14.3	1.7	100.1	Low

Data Quality Handling Report

Feature Name	Var Type	Missing Count	Missing Percentage	Imputation Name	Imputation Description
Galectin3	Numeric	644	22	Missing Values Imputed	Missing indicator treated as feature, Imputed value: 14.2
TnThs	Numeric	86	3	Missing Values Imputed	Missing indicator treated as feature, Imputed value: 10
pbnpl1	Numeric	46	2	Missing Values Imputed	Missing indicator treated as feature, Imputed value: 271
vitd25	Numeric	13	0	Missing Values Imputed	Missing indicator treated as feature, Imputed value: 16.1
crp	Numeric	6	0	Missing Values Imputed	Missing indicator treated as feature, Imputed value: 3.9
cystatc	Numeric	6	0	Missing Values Imputed	Missing indicator treated as feature, Imputed value: 0.9
hba1c	Numeric	3	0	Missing Values Imputed	Missing indicator treated as feature, Imputed value: 6
elpa	Numeric	3	0	Missing Values Imputed	Missing indicator treated as feature, Imputed value: 16
smoclass (Categorical Int)	Categorical	0	0	Ordinal encoding of categorical variables	Imputed value: -2

sex (Categorical Int)	Categorical	0	0	Ordinal encoding of categorical variables	Imputed value: -2
vitd125	Numeric	0	0	Missing Values Imputed	Imputed value: 33.4
age	Numeric	0	0	Missing Values Imputed	Imputed value: 62.8135
