# Supplementary material for: Cardiovascular risk assessment enhanced by automated machine learning in a multi-phase study
Source: Sci Rep. 2025 Oct 20;15:36474. doi: 10.1038/s41598-025-24189-z (PMC12537956; doi:10.1038/s41598-025-24189-z)
Supplement: Supplementary file 9 — Supplementary Material 9 [file 41598_2025_24189_MOESM9_ESM.pdf]

Section/Topic	Item	Checklist Item	Page
**Title and Abstract**			
**Title**	1	Identify the study as developing and/or validating a multivariable prediction model, the target population, and the outcome to be predicted.	p. 1
**Abstract**	2	Provide a summary of objectives, study design, setting, participants, sample size, predictors, outcome, statistical analysis, results, and conclusions.	p. 2 (some items were moved to other sections of the manuscript)
**Introduction**			
**Background and objectives**	3a	Explain the medical context (including whether diagnostic or prognostic) and rationale for developing or validating the multivariable prediction model, including references to existing models.	pp. 3-5
	3b	Specify the objectives, including whether the study describes the development or validation of the model or both.	p. 5
**Methods**			
**Source of data**	4a	Describe the study design or source of data (e.g., randomized trial, cohort, or registry data), separately for the development and validation data sets, if applicable.	p. 6
	4b	Specify the key study dates, including start of accrual; end of accrual; and, if applicable, end of follow-up.	p. 6, references
**Participants**	5a	Specify key elements of the study setting (e.g., primary care, secondary care, general population) including number and location of centres.	p. 6, references
	5b	Describe eligibility criteria for participants.	p. 6, references
	5c	Give details of treatments received, if relevant.	p. 6, references
**Outcome**	6a	Clearly define the outcome that is predicted by the prediction model, including how and when assessed.	p. 7
	6b	Report any actions to blind assessment of the outcome to be predicted.	pp. 8-9
**Predictors**	7a	Clearly define all predictors used in developing or validating the multivariable prediction model, including how and when they were measured.	pp. 7-10, Table S4-S6
	7b	Report any actions to blind assessment of predictors for the outcome and other predictors.	pp. 8-9
**Sample size**	8	Explain how the study size was arrived at.	p. 8
**Missing data**	9	Describe how missing data were handled (e.g., complete-case analysis, single imputation, multiple imputation) with details of any imputation method.	p. 8, Table S7
**Statistical analysis methods**	10a	Describe how predictors were handled in the analyses.	p. 8, Table S7
	10b	Specify type of model, all model-building procedures (including any predictor selection), and method for internal validation.	p. 8, Table S7, Figure S2, Figure 1
	10d	Specify all measures used to assess model performance and, if relevant, to compare multiple models.	p. 8
**Risk groups**	11	Provide details on how risk groups were created, if done.	NaN
**Results**			
**Participants**	13a	Describe the flow of participants through the study, including the number of participants with and without the outcome and, if applicable, a summary of the follow-up time. A diagram may be helpful.	p. 11, Figure 1A, Figure 5, Figure S1, Figure S7
	13b	Describe the characteristics of the participants (basic demographics, clinical features, available predictors), including the number of participants with missing data for predictors and outcome.	pp. 8-9, Figure S7
**Model development**	14a	Specify the number of participants and outcome events in each analysis.	pp. 11-22, see p. 19, Figure 5C
	14b	If done, report the unadjusted association between each candidate predictor and outcome.	Figure 3-5, Figure S4, S5, S7, Table S4-S6
**Model specification**	15a	Present the full prediction model to allow predictions for individuals (i.e., all regression coefficients, and model intercept or baseline survival at a given time point).	pp. 21-22
	15b	Explain how to the use the prediction model.	pp. 18, 21-22
**Model performance**	16	Report performance measures (with CIs) for the prediction model.	Table S3
**Discussion**			
**Limitations**	18	Discuss any limitations of the study (such as nonrepresentative sample, few events per predictor, missing data).	pp. 24-29
**Interpretation**	19b	Give an overall interpretation of the results, considering objectives, limitations, and results from similar studies, and other relevant evidence.	pp. 24-29
**Implications**	20	Discuss the potential clinical use of the model and implications for future research.	pp. 24-29
**Other information**			
**Supplementary information**	21	Provide information about the availability of supplementary resources, such as study protocol, Web calculator, and data sets.	pp. 35-42
**Funding**	22	Give the source of funding and the role of the funders for the present study.	p. 35
