# Supplementary material for: Cardiovascular risk assessment enhanced by automated machine learning in a multi-phase study
Source: Sci Rep. 2025 Oct 20;15:36474. doi: 10.1038/s41598-025-24189-z (PMC12537956; doi:10.1038/s41598-025-24189-z)
Supplement: Supplementary file 10 — Supplementary Material 10 [file 41598_2025_24189_MOESM10_ESM.pdf]

LURIC dataset

Name	Explanation
**ace**	ACE (U/l)
**acsyn (I20.0, I21, I22)**	Acute coronary syndrome; 1=yes; 0=no
**afibyn (I48)**	Atrial fibrillation; 1=yes; 0=no
**age**	Age in years
**anginayn (I20)**	Angina pectoris; 1=yes; 0=no
**aptt**	aPTT (sec)
**at3**	antithrombin III (%)
**bmi**	BMI
**cadyn (I20–I25)**	Coronary artery disease; 1=yes; 0=no
**canceryn (C00–C97)**	Cancer; 1=yes; 0=no
**carosten (I65.2)**	Carotid stenosis; 1=yes; 0=no
**chol**	Cholesterol (serum) (mg/dL)
**ck**	creatine kinase (U/l)
**cmpyn (I42)**	Cardiomyopathy; 1=yes; 0=no
**COPDyn (J44)**	COPD; 1=yes; 0=no
**crea**	Creatinin (mg/dL)
**crp**	CRP (mg/dL)
**cystatc**	Cystatin C (mg/l)
**ddimer**	D-dimer (mg/l)
**CV-EoL**	Patient death by cardiovascular cause in 2010; 1=yes; 0=no
**dm1yn (E10)**	Diabetes mellitus type 1; 1=yes; 0=no
**dm2yn (E11)**	Diabetes mellitus type 2; 1=yes; 0=no
**dyspneyn (R06.0)**	Dyspnea; 1=yes; 0=no
**eapoa1**	Apolipoprotein A1(mg/dL)
**eapoa2**	Apolipoprotein A2 (mg/dL)
**eapob**	Apolipoprotein B (mg/dL)
**eapoe**	Apolipoprotein E (mg/dL)
**earlycad**	Early coronary artery disease; 1=yes; 0=no
**elpa**	Lipoprotein (a) (EDTA) (mg/dL)
**etg**	Triglycerides (EDTA) (mg/dL)
**exercise**	Exercise on 1-11 scale
**ferritin**	Ferritin (ng/ml)
**fibrinog**	Fibrinogen (mg/dL)
**fii**	Factor II (U/dL)
**folicac**	Foliac acid(μg/l)
**Galectin3**	Galectin 3 (ng/ml)
**haptoglo**	Haptoglobin (mg/dL)
**hb**	Hemoglobin (g/dL)
**hba1c**	HbA1c in %
**hdlch**	HDL cholesterol (mg/dL)
**height**	Height in cm
**homocys**	Homocysteine (μmol/l)
**hypten (I10–I15)**	Hypertension; 0=no; 1=yes
**immunyn**	Immune disease; 1=yes; 0=no
**infectyn**	Infection; 1=yes; 0=no
**insuthyn**	Insuline; 1=yes; 0=no
**iron**	Iron (μg/dL)
**ishypert (I10–I15)**	Hypertension; 1=yes; 0=no
**ldh**	LDH (U/l)
**ldlch**	LDL cholesterol (mg/dL)
**ldlchol**	LDL cholesterol (mg/dL)
**ldltg**	LDL triglyceride (mg/dL)
**lvangio (I50.1)**	Left ventricular angiography results (0='?'; 1='normal'; 2='slight'; 3='moderate'; 4='severe')
**miyn (I21–I22)**	Myocardial infarction; 1=yes; 0=no
**parat**	PTH (pg/ml)
**pbnpl1**	NTproBNP (pg/ml)
**pvdyn (I70.2)**	Peripheral artery disease; 1=yes; 0=no
**quick**	Quick %
**rhythyn (I49)**	Arrythmia; 1=yes; 0=no
**sex**	Sex; male=1; female=0
**smoclass (Z72.0)**	Smoking; 0=no; 1=ex; 2=active
**statinyn**	Statin therapy; 1=yes; 0=no
**strokeyn (I63)**	Stroke; 1=yes; 0=no
**supercrp**	High sensitive CRP (mg/l)
**TnThs**	Troponin T (pg/ml)
**tpaant**	t-PA antigen (μg/l)
**tsh**	TSH hormone (mU/l)
**urea**	Urea (mg/dL)
**uricacid**	Uric acid (mg/dL)
**vdyn (I34–I39)**	Valve disease; 1=yes; 0=no
**venthrom (I82)**	Venous thrombosis; 1=yes; 0=no
**vitb12**	Vitamin B12 (ng/l)
**vitd25**	25-hydroxy vitamin D (μg/l)
**vitd125**	1-25-dihydroxy vitamin (ng/l)
**vldlch**	VLDL cholesterol (mg/dL)
**vldltg**	VLDL triglycerides (mg/dL)
**vwfag**	vWillebrand factor antigen (U/dL)
**weight**	weight in kg

UMC/M dataset

Name	Explanation
**acsyn (I20.0, I21, I22)**	Acute coronary syndrome; 1=yes; 0=no
**age**	Age in years
**aht (I10–I15)**	Arterial hypertension; 1=yes; 0=no
**alipo**	A-Lipo (mg/dL)
**alipo2**	A-Lipo2 (mg/dL)
**ApoA1**	Apolipoprotein A1 (mg/dL)
**ApoB**	Apolipoprotein B (mg/dL)
**b_lipo2**	B Lipo2 (mg/dL)
**blipo**	B Lipo (mg/dL)
**BMI**	BMI
**BMI cat**	BMI categorical
**cadyn (I20–I25)**	Coronary artery disease; 1=yes; 0=no
**bypass_surgery (Z95.1)**	Bypass surgery; 1=yes; 0=no
**CHA2DS2_VASc_Score**	CHADS-VASC score
**chol**	Cholesterol (mg/dL), measured by lipid electrophoresis
**chol2**	Cholesterol 2 (mg/dL), measured in plasma
**CK**	CK (U/l)
**CK2**	CK2 (U/l)
**COPD (J44)**	COPD; 1=yes; 0=no
**CRP**	High sensitive CRP (mg/l)
**ddysfct (I50.0)**	Diastolic dysfunction; 1=yes; 0=no
**dm1 (E10)**	Diabetes mellitus 1; 1=yes; 0=no
**dm2 (E11)**	Diabetes mellitus 2; 1=yes; 0=no
**earlycvevent**	Early (cardio-) vascular event; 1=yes; 0=no
**ezetimibe**	Ezetimibe treatment; 1=yes; 0=no
**ezetimibe_at0**	Ezetimibe after enrollment (t0); 1=yes; 0=no
**ezetimibe_pt0**	Ezetimibe prior to enrollment (t0); 1=yes; 0=no
**FHscore**	FH score
**HbA1c**	HbA1c in %
**HbA1c cat**	HbA1c categories
**HbA1c_at0**	HbA1c in % after enrollment (t0)
**HDL**	HDL (mg/dL)
**HDL2**	HDL 2 (mg/dL)
**HDLalphafract**	HDL alpha fraction (mg/dL)
**homocys**	Homocysteine (μmol/l)
**LDL**	LDL (mg/dL), measured by lipid electrophoresis
**LDL_C**	LDL cholesterol (mg/dL)
**LDL_categorical**	LDL categories
**LDL2**	LDL2 (mg/dL), measured in plasma
**LDLbetafract**	LDL beta fraction (mg/dL)
**LDLcatyn**	LDL categorical; 1=yes; 0=no
**LPA**	Lipoprotein (a) (mg/dL)
**LPAov50**	Lipoprotein (a) over 50mg/dL; 1=yes; 0=no
**LVF (I50.1)**	Left ventricular function categories
**LVFhireduced**	Left ventricular function highly reduced; 1=yes; 0=no
**NPPB**	NTproBNP (ng/l)
**NPPB_GFR**	NTproBNP GFR corrected (ng/l)
**otherlipidpharm**	Other lipid reducing pharmaceuticals
**pos_ FA (Z82.49)**	Positive family history; 1=yes; 0=no
**prebfract**	Pre beta fraction (mg/dL)
**PSCK9**	PCSK9 inhibitor use; 1=yes; 0=no
**regCRP**	CRP (mg/l)
**sex**	Sex; 1=male; 0=female
**SGLT2at0**	SGLT2 inhibitor use after enrollment (t0); 1=yes; 0=no
**SGLT2pt0**	SGLT2 inhibitor use prior to enrollment (t0); 1=yes; 0=no
**smoke (Z72.0)**	Smoking status; 1=smoking; 0=not smoking
**statin_at0**	Statin after enrollment (t0); 1=yes; 0=no
**statin_pt0**	Statin prior to enrollment (t0); 1=yes; 0=no
**TG**	Triglycerides (mg/dL)
**TG2**	Triglycerides 2 (mg/dL)
**VLDLprebetafract**	VLDL beta fraction (mg/dL)
**vitD**	Vitamin D (μg/l)
