# Supplementary material for: Cardiovascular risk assessment enhanced by automated machine learning in a multi-phase study
Source: Sci Rep. 2025 Oct 20;15:36474. doi: 10.1038/s41598-025-24189-z (PMC12537956; doi:10.1038/s41598-025-24189-z)
Supplement: Supplementary file 11 — Supplementary Material 11 [file 41598_2025_24189_MOESM11_ESM.pdf]

Supplementary Materials – Figure Legends

Supplementary Figure S1. Study Outline. This graphic illustrates our study design, which has a tripartite structure. First, the "LURIC" and "UMC/M" datasets were prepared for AutoML use by preprocessing the dataset features and generating matching feature lists for the ten targets of the target groups Lp(a) and specific CVDs. In the second phase of the study, we wanted to test new models based on a reduced feature list using only the common features between the two datasets. Models were trained on the LURIC-Common data and separately validated on the UMC/M-Common data set. This approach resulted in the following specific CVDs models: 'CAD Common', 'MI Common', 'Stroke Common', 'PAD Common' and 'ACS Commo'n. Finally, in Phase 3, we used the target cardiovascular End-of-Life (EoL) to train models to predict this specific outcome in the LURIC dataset on four different feature lists (LURIC-Common/EoL-1 and EoL 2-4).

Supplementary Figure S2. Model blueprints for phases 2 and 3 of the study. Individual blueprints for the modelling steps for each target in phase 2 and 3 are shown graphically. Starting from target selection in the dataset, through the modelling steps, to the individual models that were used to make predictions.

Supplementary Figure S3. Model lift charts for ML models selected in Phase 1. Lift charts for all Phase 1 targets 'LPA-L' (A), 'CAD-L' (B), 'early CAD-L' (C), 'MI-L' (D), 'Stroke-L' (E), 'PAD-L' (F), 'LPA-U' (G), 'CAD-U' (H), 'early CV conditions-U' (I) and 'ACS-U' (J) are shown here again, but individually for a better analytical view. In each lift chart, the x-axis represents data grouped into equal-sized bins, while the y-axis represents the average target value or 'lift'. For each bin, the chart shows the average actual and predicted values for the data set. The actual values describe the percentage of rows in a given bin that have the target score for the feature. On the left side of the curve, the bins show where the model predicts a low probability for the target feature, while the right side shows a high probability. The steeper the actual line and the closer it is to the predicted values, the better the model is at segmenting the data and predicting the target feature.

Supplementary Figure S4. Further selection of model Feature Effects for Phase 1. Plots of selected Feature Effects for 'LPA-L' (A), 'CAD-L' (B), 'early CAD-L' (C), 'MI-L' (D), 'Stroke-L' (E), 'PAD-L' (F), 'LPA-U' (G), 'CAD-U' (H), 'early CV conditions-U' (I), 'ACS-U' (J). Feature effects show mean predicted values as a function of different variable inputs for the specific

feature and partial dependence (PD) plots. The PD shows the change in target predictions when a particular feature is changed while all other features are held constant.

Supplementary Figure S5. SHAP-based feature effect plot for selected machine learning models targeting 'CAD Common', 'MI Common', 'Stroke Common', 'PAD Common' and 'ACS Common'. The models were trained and cross-validated on the "LURIC Common" dataset and again externally validated on the "UMC/M Common" dataset. The SHAP-based plots are all generated from the external validation with the secondary dataset "UMC/M Common". (A) SHAP beeswarm plot for the selected models. Each point represents a SHAP value for a feature and a single instance. The x-axis depicts the SHAP values, indicating the impact of the feature on the model output, while the y-axis lists the features in descending order of importance. SHAP feature values are also displayed in colour, with red denoting high feature values, blue denoting low feature values and grey denoting missing data points. (B) SHAP decision plot for the selected models. The x-axis represents the model output value (log odds) and the y-axis lists the features. Each line starts at the base value of the model and shows the contribution of each feature's SHAP value to the final prediction, moving from bottom to top. (C) SHAP heatmaps showing the importance of features across the dataset. Each row corresponds to a feature and each column on the x-axis represents an instance, i.e. a specific data point, in the "UMC/M-Common" dataset. The colour intensity of each cell indicates the SHAP value, with red indicating a positive effect and blue indicating a negative effect on the model's prediction. The black line graph at the top shows the model's prediction output, f(x), for each instance. The mean SHAP values are shown as black bars on the right hand side of the plot. (D) SHAP prediction explanations as waterfall plots for a hypothetical patient. E[f(x)] represents the output of the base model (the expected log odds without any feature contributions) and f(x) is the individual outcome in log odds. Each bar stands for the contribution of one feature, with the bars adding up to the final prediction. The colour of the bars denotes the direction and magnitude of the effect of each feature, with positive contributions in red and negative contributions in blue.

Supplementary Figure S6. Model ROC curves and lift charts for ML models selected in Phase 3. ROC curves (left) and lift charts (right) are shown individually for each EoL model, namely EoL-1 (A), EoL-2 (B), EoL-3 (C) and EoL-4 (D). ROC curves for all models plot false positive rates against true positive rates. The diagonal black line is the line of identity. In the case of lift charts, the colours represent individual models with their average data values for actual and predicted outcomes segmented into equally sized data bins. Each bin can be traced

to its corresponding y-axis value, which represents the percentage of data that falls within the target value.

Supplementary Figure S7. SHAP-based feature effect plot for selected machine learning models targeting EoL. The SHAP-based plots are all generated from training partition of their respective EoL feature lists of the LURIC dataset. SHAP beeswarm plot (A) for the selected models. Each point represents a SHAP value for a feature and a single instance. The x-axis depicts the SHAP values, indicating the impact of the feature on the model output, while the y-axis lists the features in descending order of importance. SHAP feature values are also displayed in colour, with red denoting high feature values, blue denoting low feature values and grey denoting missing data points. SHAP decision plot (B) for the selected models. The x-axis represents the model output value (log odds) and the y-axis lists the features. Each line starts at the base value of the model and shows the contribution of each feature's SHAP value to the final prediction, moving from bottom to top. SHAP heatmaps (C) showing the importance of features across the dataset. Each row corresponds to a feature and each column on the x-axis represents an instance, i.e. a specific data point, in the training partition. The colour intensity of each cell indicates the SHAP value, with red indicating a positive effect and blue indicating a negative effect on the model's prediction. The black line graph at the top shows the model's prediction output, f(x), for each instance. The mean SHAP values are shown as black bars on the right hand side of the plot.
