# Supplementary material for: Cardiovascular risk assessment enhanced by automated machine learning in a multi-phase study
Source: Sci Rep. 2025 Oct 20;15:36474. doi: 10.1038/s41598-025-24189-z (PMC12537956; doi:10.1038/s41598-025-24189-z)
Supplement: Supplementary file 12 — Supplementary Material 12 [file 41598_2025_24189_MOESM12_ESM.pdf]

Supplementary Materials – Table Legends and Method Legends

Supplementary Table S1. Feature transformations and modifications made to the primary datasets. Feature transformations and modifications performed on the UMC/M primary dataset are listed above. The only feature transformation performed for both datasets was the feature "Lipoprotein (a) over 50mg/dL". All other features of the primary datasets were not modified.

Supplementary Table S2. Explanation of feature name abbreviations. Abbreviated features that occur in both datasets are explained with the appropriate nomenclature, categories, scales and ICD-10 codes if applicable.

Supplementary Table S3. Final feature lists for each target used in the second and third phase of the study. For the second phase of the study, we selected five models for the specific CVD targets of ACS, PAD, CAD, MI and stroke. Each of the models was created using the 'LURIC-Common' feature list from the LURIC dataset. After model selection, we uploaded a secondary dataset, the 'UMC/M-Common' dataset, containing only the common features found in 'LURIC-Common'. To ensure that no data leakage occurred, we excluded the features 'cad', 'early cad', 'miyn' for the 'ACS LURIC-Common' model; 'early cad', 'miyn', 'acsyn' for the 'CAD LURIC-Common' model and 'acs', 'early cad', 'cad' for the 'MI LURIC-Common' model. Notably, for the Stroke LURIC-Common model, our AutoML platform automatically reduced the feature set to 16 out of 36 original features for better model performance. For simplicity, in the third phase of the study, the feature lists have the same names as their targets, with the exception of the feature list 'LURIC-Common/EoL-1' to highlight the additional feature called 'CV-EoL'. The features that have been selected as targets are shown in bold.

Supplementary Table S4. Performance metrics for all selected machine learning models. For all three study phases, each target of a specific target group, namely Lp(a), specific CVDs or EoL, is listed with the total number of ML models trained and compared during the AutoML process and the final ML model selected. Note that L1 and L2 in some model names correspond to Lasso and Ridge Regularization, a modelling technique to avoid overfitting. Model selection was based on the best ranked area under the curve (AUC) value for the cross-validation (CV) data partition, which is highlighted in bold. Other metrics shown

are the Logarithmic Loss (LogLoss) and the Maximum Mathew's Correlation Coefficient (Max MCC) for each data partition. To provide confidence measures, we reran the selected models a total of 10 times using 10 different data partitioning 'seeds'. This allowed us to assess the consistency of model performance across the datasets when the datasets were randomly shuffled for partitioning. The lower and upper 95% confidence intervals are given in parentheses, and the performance metrics are given as means. Target names: LPA: Patients with lipoprotein (a) above 50mg/dL; CAD: Patients with coronary artery disease; Early CAD: Patients with early coronary artery disease; MI: Patients with myocardial infarction; Stroke: Patients with stroke; PAD-L: Patients with peripheral artery disease; Early CV conditions-U: Patients with early cardiovascular conditions (early cardiovascular disease, early chronic venous insufficiency and peripheral artery disease); ACS: Patients with acute coronary syndrome.

Supplementary Table S5. Final feature lists for each target used with the LURIC dataset in the first phase of the study. After uploading the LURIC dataset to the AutoML platform, we manually selected features from the dataset that were important for the modelling process. After data leakage was identified in the exploratory data analysis, we manually removed these potential data leakage features. The AutoML platform also further reduced the feature lists to ensure better performance for modelling. The final feature lists for the selected models are shown in this table for the LURIC models in the first phase of the study. The features that have been selected as targets are shown in bold.

Supplementary Table S6. Final feature lists for each target used with the UMC/M dataset in the first phase of the study. After uploading the UMC/M dataset to the AutoML platform, we manually selected important dataset features for the modelling process. After data leakage was identified in the exploratory data analysis, we manually removed these potential data leakage features. The AutoML platform also further reduced the feature lists to ensure better performance for modelling. The final feature lists for the selected models are shown in this table for the UMC/M models in the first phase of the study. Numbers preceding specific feature names refer to individual study visits in the UMC/M dataset. The features that have been selected as targets are shown in bold.

Supplementary Table S7. Information on model development and data quality handling. All the models selected for the study are presented here, along with means of data partitioning during training, descriptive statistics, as well as information on data leakage and a data quality report on the imputation of missing values.

Supplementary Table S8. TRIPOD Checklist for Prediction Model Development. This study adheres to the TRIPOD standards as documented. As predictions were made mainly in Phase 2 and partly in Phase 3, some items do not apply for all parts of this article.

Supplementary Methods. Custom Code for SHAP based plot creation from the DataRobot platform. Since model development was performed entirely using the user interface of the DataRobot AutoML platform, no custom code was written for this purpose. However, we provide the code used to derive SHAP plots from the platform outputs.
